# Potential biomarkers for predicting the overall survival outcome of kidney renal papillary cell carcinoma: an analysis of ferroptosis-related LNCRNAs

**DOI:** 10.1186/s12894-022-01037-0

**Published:** 2022-09-14

**Authors:** Zixuan Wu, Xuyan Huang, Minjie Cai, Peidong Huang

**Affiliations:** 1 Yunnan University of Chinese Medicine, Kunming, 650500 Yunnan Province China; 2 Guangzhou University of Chinese Medicine, Guangzhou, 510006 Guangdong Province China; 3 Shantou Health School, Shantou, 515061 Guangdong Province China; 4 Second Affiliated Hospital of Yunnan University of Chinese Medicine, No. 161 Dongjiao Road, Guandu District, Kunming, 650504 Yunnan Province China

**Keywords:** Kidney renal papillary cell carcinoma (KIRP), Ferroptosis-related LNCRNAs, TCGA datasets, Immune cell infiltration, Bioinformatics analysis

## Abstract

**Background:**

Kidney renal papillary cell carcinoma (KIRP) is a dangerous cancer, which accounts for 15–20% of all kidney malignancies. Ferroptosis is a rare kind of cell death that overcomes medication resistance. Ferroptosis-related long non-coding RNAs (LNCRNAs) in KIRP, remain unknown.

**Method:**

We wanted to express how ferroptosis-related LNCRNAs interact with immune cell infiltration in KIRP. Gene set enrichment analysis in the GO and KEGG databases were used to explore gene expression enrichment. The prognostic model was constructed using Lasso regression. In addition, we also analyzed the modifications in the tumor microenvironment (TME) and immunological association.

**Result:**

The expression of LNCRNA was closely connected to the ferroptosis, according to co-expression analyses. CASC19, AC090197.1, AC099850.3, AL033397.2, LINC00462, and B3GALT1-AS1 were found to be significantly increased in the high-risk group, indicating that all of these markers implicates the malignancy processes for KIRP patients and may be cancer-promoting variables. LNCTAM34A and AC024022.1 were shown to be significantly elevated in the low-risk group; these might represent as the KIRP tumor suppressor genes. According to the TCGA, CCR, and inflammation-promoting genes were considered to be significantly different between the low-risk and high-risk groups. The expression of CD160, TNFSF4, CD80, BTLA, and TNFRSF9 was different in the two risk groups.

**Conclusion:**

LNCRNAs associated with ferroptosis were linked to the occurrence and progression of KIRP. Ferroptosis-related LNCRNAs and immune cell infiltration in the TME may be potential biomarkers in KIRP that should be further investigated.

**Supplementary Information:**

The online version contains supplementary material available at 10.1186/s12894-022-01037-0.

## Introduction

Kidney cancer has become a commonly diagnosed cancer type around the world [1]. It is the second most common genitourinary system tumor in China, and the trend is increasing yearly [2]. Kidney renal papillary cell carcinoma (KIRP), which accounts for 15–20% of all kidney malignancies, arise from the proximal nephron, the same source as clear cell carcinoma [3]. However, in terms of disease development and patient survival outcome, KIRP is found to be a more diverse disease [4]. KIRP could be addressed in a variety of methods, including surgery, radiation, and chemotherapy, but these only provide a minor benefit [5, 6]. KIRP patients are frequently excluded from genetic studies and randomized clinical trials for kidney cancer because of the limited number of cases [7]. Yet, a lack of accurate biomarkers for early tumor detection and limited preclinical models have hampered efficient KIRP treatment therapy [8, 9]. Additional molecular identification is critical for both basic and clinical research of KIRP, in order to avoid the early onset and progression of KIRP as well as the development of novel and effective prognostic biomarkers.

LNCRNAs are a type of RNA molecules that has high levels of expression selectivity. Several studies have found that the LNCRNAs are involved in a wide range of biological activities, including gene regulation, tumor incidence, development, and metastasis regulation [10, 11]. The LNCRNAs might work together to promote the c-Myc expression and activate the Wnt signaling pathway, which is critical in developing colorectal cancer [12]. Meanwhile, the LNCRNA TUG1 was increased in hepatoblastoma, stimulating the downstream signaling pathway of JAK2/STAT3 and promoting angiogenesis in hepatoblastoma cells [13]. In non-small cell lung cancer, the LNCRNA HLA complex group 11 was demonstrated in reducing malignancy by eliminating the expression levels of carcinogenic microRNA875 [14]. However, transfection with LNCRNA short nucleolar RNA host gene eight reduced RASA1 expressions, shielding H9C2 cells against HI/R damage [15]. In recent years, ferroptosis of tumor cells has attracted a lot of attention as a new type of cell death that enables tumor cells in overcoming treatment resistance [16, 17]. Unlike apoptosis and autophagy, ferroptosis is a kind of iron-dependent and reactive oxygen species (ROS)-dependent cell death that is utilized to treat a range of disorders. Because cancer cells are more iron-dependent than normal cells and rely on it too much to proliferate, an imbalance in iron metabolism accelerates tumor growth [18]. Ferroptosis pathway activation surpasses current resistance to chemotherapeutic medications, opening up a further therapeutic frontier for cancer treatment [19]. LNCRNAs have been shown to stimulate ferroptosis regulation, control iron death, and cell apoptosis, whereas silencing the LNCRNAs considerably reduce ferroptosis and regulate inflammation and lipid peroxidation [20, 21]. Despite this, there have been rare sequence-based investigations on aberrant LNCRNA expression and its relationship with overall survival (OS) outcome of KIRP patients with iron addiction.

Immune checkpoint-related gene profiles is helpful in detecting treatment responsiveness, as well as evaluating risks and predicting survival rate of KIRP patients [22]. Despite little study has been done on the association between ferroptosis-related LNCRNAs and immune cell infiltration in KIRP, it is crucial to look into immune cell infiltration in the TME and its relationship with KIRP clinicopathological characteristics of the tumor. The causes and mechanisms of the aberrant LNCRNA expression and ferroptosis in KIRP are currently unknown. To understand the LNCRNA-related pathways that influence KIRP patients' prognosis, it is essential to construct transcriptional maps of LNCRNA expression and ferroptosis change in KIRP patients. To assess the risk and predict the overall survival outcome in KIRP patients, immune checkpoint-related gene profiles can be invoked as a predictor of therapy responsiveness. Figuring out how ferroptosis-related LNCRNAs influence the KIRP progression could lead to the discovery of a biomarker that could be exploited as a therapeutic target.

## Materials and methods

We followed the methods of Yun Tang et al. 2021 [23].

### Datasets and ferroptosis-related genes

The Cancer Genome Atlas was used to collect BLCA gene expression patterns and clinical data (TCGA) [24]. The expression patterns of 298 KIRP and 32 normal tissues were enrolled in the TCGA on December 3, 2021. Table 1 summarizes the clinical features of the patients. In addition, corresponding ferroptosis-related human genes were downloaded from FerrDb [25], a web-based consortium that provides a comprehensive and up-to-date database for ferroptosis markers, their regulatory molecules, and associated diseases. We identified a total of 382 ferroptosis-related genes (driver: 150; suppressor: 109; and marker: 123) (Additional file 1: Table S1a–c).

**Table 1:** The clinical characteristics of patients in the TCGA dataset

Variable	Number of samples
Gender	
Male/female	214/77
Age at diagnosis	
≤ 65/ > 65	179/109
Grade	
G1/G2/G3/G4/NA	Unknow
Stage	
I/II/III/IV/NA	173/21/52/15/30
T	
T1/T2/T3/T4/NA	194/33/60/2/2
M	
M0/M1/NA	95/9/187
N	
N0/N1/N2/NA	50/24/4/213

### Annotation of the LNCRNAs

For annotation of the LNCRNAs in the TCGA dataset, Genome Reference Consortium Human Build 38 (GRCh38) LNCRNA annotation file was obtained from the GENCODE website^4^. Perl matched and sorted transcription data and human configuration files to acquire exact mRNA and LNCRNA data. The gene IDs were converted into gene names using the informations from the database. The R4.1.0 Limma package was used to extract ferroptosis-related gene expression data, which was based on the gene expression matrix of ferroptosis-related LNCRNA gene expression profile data that was previously collected.

### Identification of the ferroptosis-related LNCRNAs

To investigate the relationship between difference ferroptosis-related LNCRNAs and KIRP, the PPI network of the target was obtained by using the String online tool [26]. Limma package's correlation test was performed to evaluate the expression of ferroptosis-related LNCRNA. Co-expression analysis was utilized to look at the relationship between ferroptosis-related gene expression and LNCRNAs. The clinical-pathology information acquired from the KIRP patients included gender, age, stage, grade, TMN, survival status, and survival time. To determine whether there was a significant difference in expression of ferroptosis-related LNCRNAs, FDR < 0.05 and |log2FC|≥ 1 were utilized by the limma package. First, we investigated into the function of ferroptosis-related differentially expressed genes that were both upregulated and downregulated (DEGs). The biological pathways connected with the DEGs were then analyzed using the Gene Ontology (GO). Biological processes (BP), molecular functions (MF), and cellular components (CC) regulated by the differently expressed ferroptosis-related LNCRNAs were further analyzed using a clusterProfiler, org.Hs.eg.db, enrichplot, and ggplot2 package. Based on data from the Kyoto Encyclopedia of Genes and Genomes (KEGG). In the same approach, we did a KEGG analysis on DEGs.

### Development of the ferroptosis-related LNCRNAs prognostic signature

To construct a Prognostic Model, first, we grouped and merged the survival data with the LNCRNAs produced from the different analysis using the Limma package and then using the Survival package, a ferroptosis-related LNCRNA signature was built using a Univariate Cox regression analysis. Finally, using Lasso-penalized Cox regression and Cox regression analysis stratified by risk score, a signature of ferroptosis-related LNCRNAs was created utilizing the Library Glmnet, Survival, and SurvMiner Packages (Coefficient LNCRNA_1_ × expression of LNCRNA_1_) + (Coefficient LNCRNA_2_ × expression of LNCRNA_2_) + ^…^ + (Coefficient LNCRNA_n_ × expression LNCRNA_n_). Each KIRP patient's associated risk score was further evaluated. Based on the median score, the RNAs were divided into two subgroups: low- and high-risk. In Lasso regression, the low-risk (50%) and high-risk (50%) groups were identified, and the corresponding plots were obtained. The confidence interval and risk ratio were calculated after visualization, and the forest diagram was constructed. The high-risk and low-risk groups' survival curves were constructed and compared. We used the timeROC program to create a similar receiver-operating characteristics (ROC) curve to test our model's accuracy in predicting the survival outcome of KIRP patients. An independent prognosis study was carried out ensuring that our model was unaffected by other clinical prognostic variables that influences the patients' outcome. Determining the association between clinical characteristics and our prediction risk model, as well as distinguishing between the high-risk and low-risk ferroptosis-related cases. Risk and clinical correlation analyses were completed. Heatmap and limma packages were used to construct the Heatmap. To further demonstrate the correctness of our model, Decision Curve Analysis (DCA) was constructed.

### Gene set enrichment analysis and the predictive nomogram

The GSEA was used to find the differences in linked functions and pathways in several samples, and data was imported using the PERL programming language. The associated score and graphs were used to determine whether or not the functions and routes in the various risk categories were dynamic. (c2.cp.kegg.v.7.2.symbols.gmt, Risk.cls#h versus l). Depending on it was a high-risk cluster of prognosis-related LNCRNAs, each sample was labeled as “H” or “L”. The number of permutations, no collapses, and phenotype were set to 1000. The gene list was sorted in “real” mode, with the order of the genes in “descending” mode. The “Signal2Noise” measure was utilized in ranking the genes. The normalization method was “meandiv,” and the difference was statistically significant with a FDR < 0.05. A nomogram was constructed integrating the prognostic signatures, for predictive of 1, 2, and 3 year OS of KIRP patients.

### Immunity analysis and gene expression

Simultaneously, the CIBERSORT [27, 28], ESTIMATE [29], MCPcounter [30], single-sample gene set enrichment analysis (ssGSEA) [31], and TIMER [32] algorithms were compared to evaluate cellular components or cell immune responses between the high and low risk groups based on ferroptosis-related LNCRNA signatures. First, we extracted gene expression data from the normal samples for ssGSEA analysis and adjusted the ssGSEA score. Then combined it with the risk data derived from the previous model construction. Finally, the immune function score was computed and shown using a boxplot. A heatmap was utilized to discover changes in immune response under different algorithms. In addition, ssGSEA was utilized to compare and quantify the tumor-infiltrating immune cell subgroups in both groups, as well as their immunological functions.

### Statistical analysis

The data was analyzed using Bioconductor programs in R software version 4.1.0. To investigate the normally and non-normally distributed variables, the Wilcoxon test and the unpaired student's t-test were utilized. The Benjamini–Hochberg technique was used to determine the variable expressed LNCRNAs based on FDR. Utilizing "GSVA" and ssGSEA-normalized KIRP DEGs, the KIRP DEGs were compared to a genome (R-package). The sensitivity and specificity of the KIRP generates a prognostic signal in comparison with other clinicopathological factors were evaluated using the operating characteristic curve (ROC) and decision curve analysis (DCA). The connection between the ferroptosis-related LNCRNAs and clinicopathological symptoms was investigated using a logistic regression analysis and a heatmap graph. Based on the ferroptosis-related LNCRNAs signature, the Kaplan–Meier survival analysis was used to estimate the survival outcome of KIRP patients. For each analysis, statistical significance was identified as *P* < 0.05.

## Results

### Enrichment analysis of ferroptosis-related genes

**Fig. 1:**
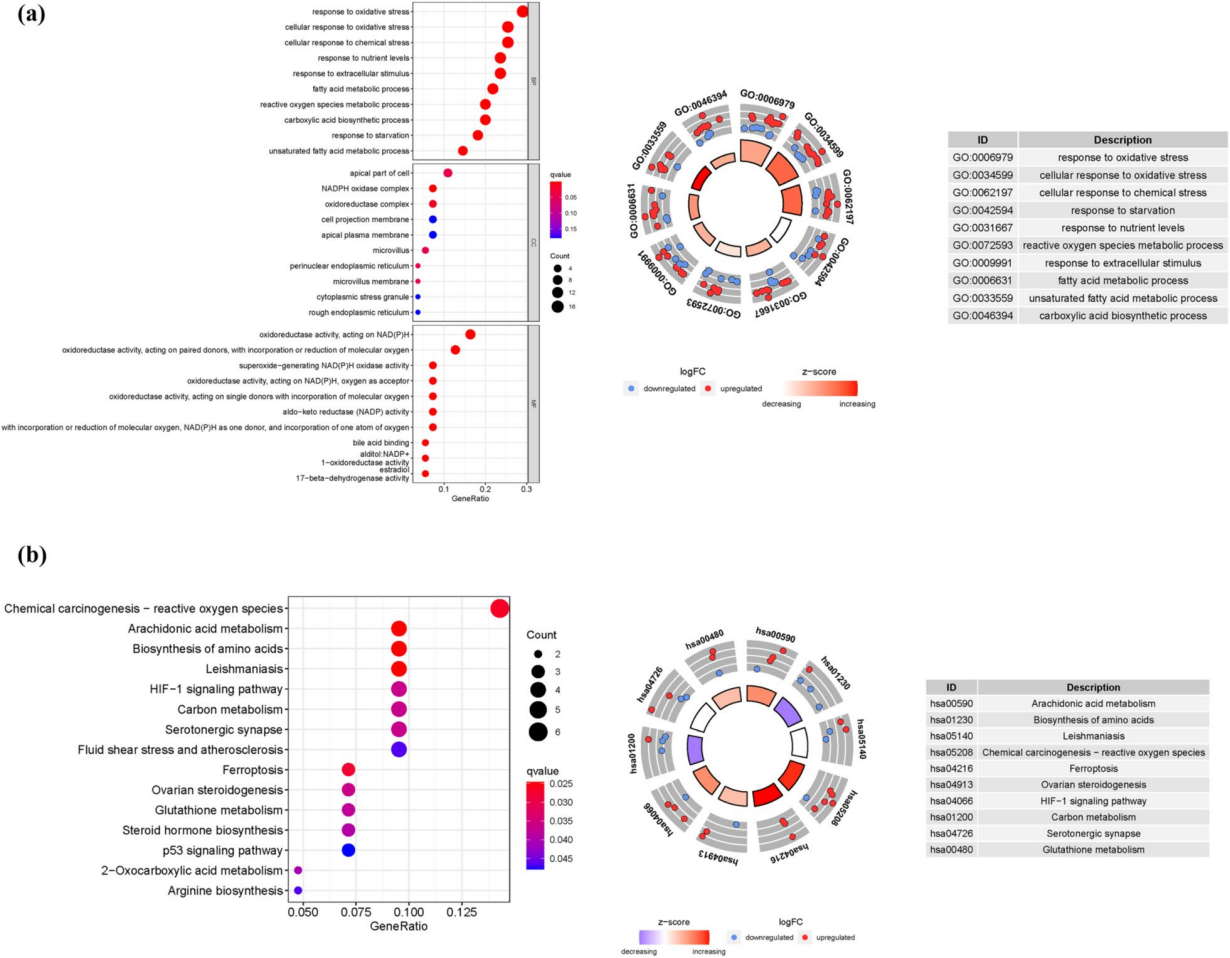
GO and KEGG analysis. **a** GO **b** KEGG. (The bigger the bubble means the more genes enriched, and the increasing depth of red means the differences were more obvious; Circle plot shows the top ten enriched GO terms for the DEGs identified between high- and low-risk groups, red means upregulated, blue means downregulated)

We discovered 56 DEGs linked to ferroptosis (24 downregulated and 32 upregulated; Additional file 1: Table S2). We obtained 427 core targets for GO analysis using the pvalueFilter = 0.05 and qvalueFilter = 0.05, including MF, CC, and BP. The MF involves oxidoreductase activity, acting on NAD(P)H (GO:0016651), oxidoreductase activity, acting on paired donors, with incorporation or reduction of molecular oxygen (GO:0016705), oxidoreductase activity, acting on the CH-OH group of donors, NAD or NADP as acceptor (GO:0016616), oxidoreductase activity, acting on CH-OH group of donors (GO:0016614). The CC mainly involves apical part of cell (GO:0045177), NADPH oxidase complex (GO:0043020), oxidoreductase complex (GO:1990204), microvillus (GO:0005902). The BP mainly involves response to oxidative stress (GO:0006979), cellular response to oxidative stress (GO:0034599), cellular response to chemical stress (GO:0062197), response to nutrient levels (GO:0031667). In addition, the main signaling pathways were identified by KEGG enrichment analysis, revealed that the over-expressed genes were mainly involved in Chemical carcinogenesis-reactive oxygen species (hsa05208), Arachidonic acid metabolism (hsa00590), HIF-1 signaling pathway (hsa04066), Ferroptosis (hsa04216), p53 signaling pathway (hsa04115) (Fig. 1a, b and Additional file 1: Table S3a–b).

### Development of a prognostic gene model and analysis of the survival outcome

**Fig. 2:**
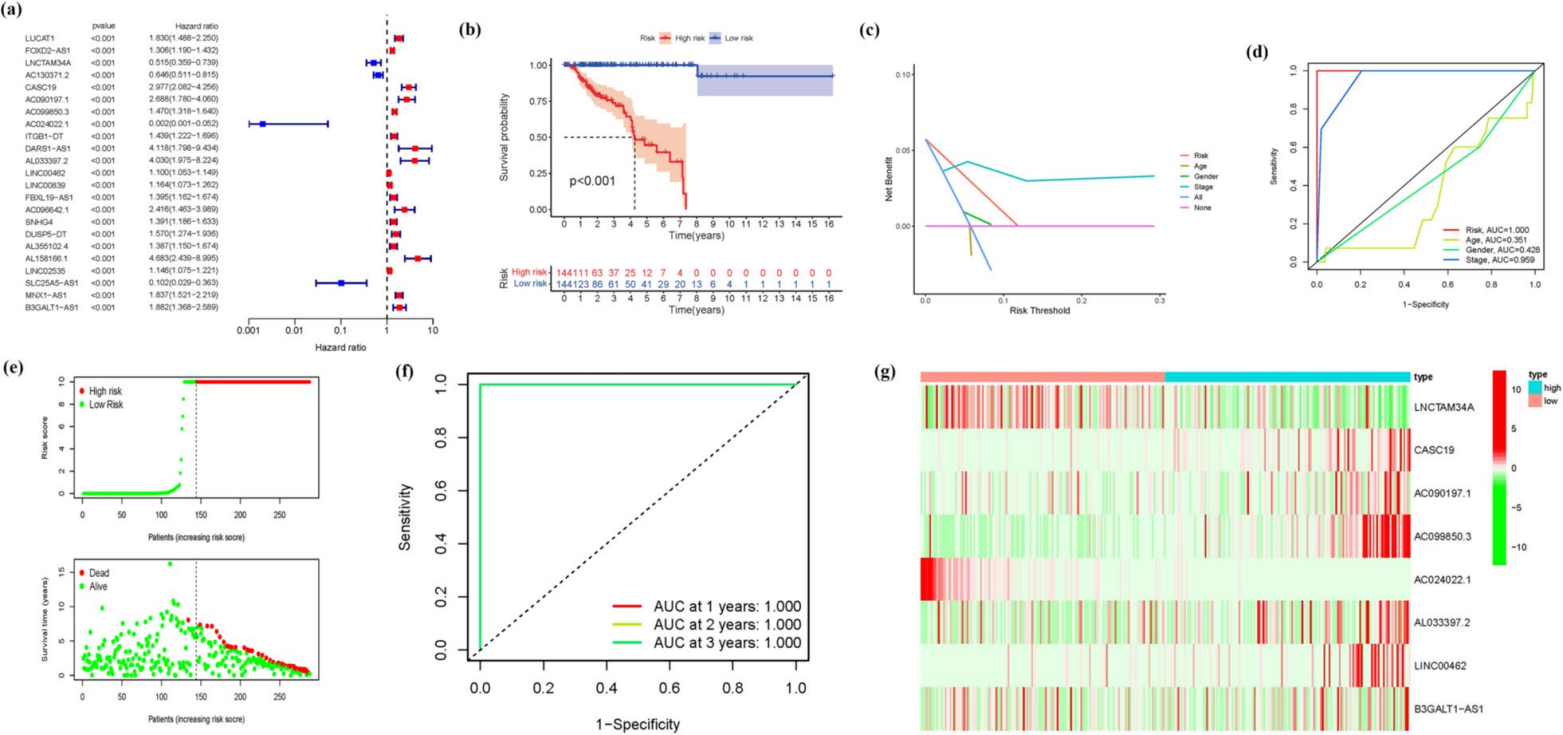
Ferroptosis-related LNCRNAs signature. **a** Univariate Cox regression illustrated 23 ferroptosis-related LNCRNAs associated with prognosis. **b** Kaplan–Meier plot for overall survival of KIRP patients in high- and low-risk groups. **c** The DCA of the risk factors. **d** The AUC values of the risk factors. **e** Distribution of risk scores based on the FRG prognostic signature. Survival status of KIRP patients with high or low risk scores. **f** The AUC of the prediction of 1, 2, 3 year survival rate of KIRP patients. **g** Heatmap (green: low expression level; red: high expression level) of the ferroptosis-related LNCRNAs

**Fig. 3:**
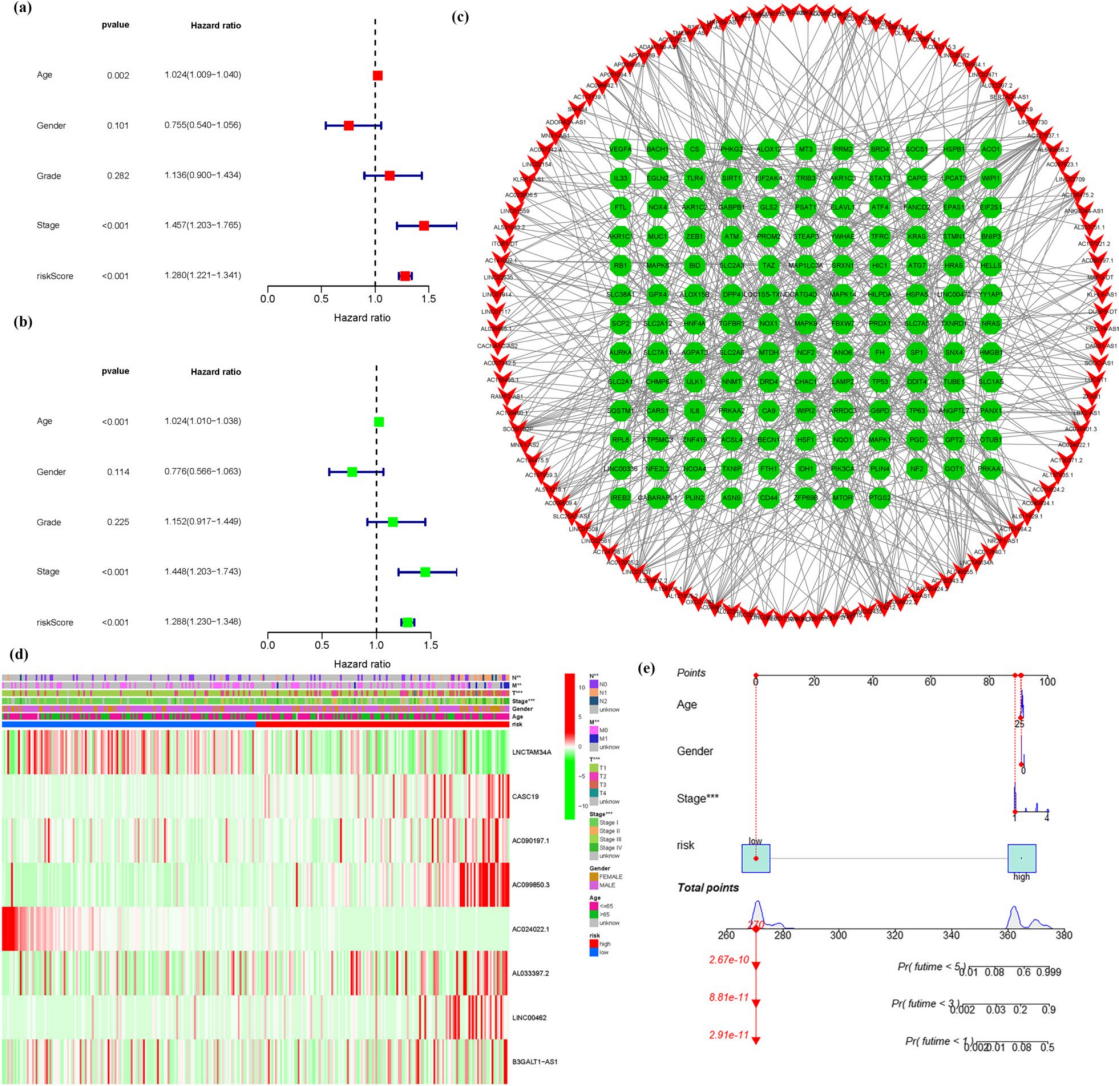
The COX analysis for the expression. **a** Univariate, **b** multivariate, (**a** + **b**) revealed IncRNA signature (HR 1.280, 95CI 1.221–1.341), age (HR 1.024, 95CI 1.009–1.040), and tumor stage (HR 1.457, 95CI 1.203–1.765) were mostly independent prognostic variables for the KIRP patients' OS. **c** The relationship between the novel LNCRNA and the mRNA expression. **d** Prognostic hallmark and clinicopathological symptoms. (N stage, M stage, T stage, and tumor stage are Prognostic hallmark related to clinicopathological symptoms.) **e** A nomogram for prognostic ferroptosis-related LNCRNAs as well as clinic-pathological variables

945 Ferroptosis-related LNCRNAs from our Prognostic Model were discovered (Additional file 1: Table S4). 23 important LNCRNAs (LUCAT1, FOXD2-AS1, LNCTAM34A, AC130371.2, CASC19, AC090197.1, AC099850.3, AC024022.1, ITGB1-DT, DARS1-AS1, AL033397.2, LINC00462, LINC00839, FBXL19-AS1, AC096642.1, SNHG4, DUSP5-DT, AL355102.4, AL158166.1, LINC02535, SLC25A5-AS1, MNX1-AS1, B3GALT1-AS1) were discovered to be independent KIRP prognostic indicators (Fig. 2a) (Additional file 1: Table S5a). As a consequence, we calculated the risk scores for the LNCRNAs and constructed a prognostic signature. Depending on the Kaplan–Meier analyses, the expression of high-risk LNCRNA signatures were associated with poor survival outcome (*P* < 0.001, Fig. 2b). Meanwhile, the signature LNCRNAs' AUC was 1, indicating that they outperformed standard clinicopathological characteristics in predicting KIRP prognosis (Fig. 2c, d). Using a patient's risk survival status plot, we observed that the patient's risk score was inversely related to the survival of KIRP patients. Surprisingly, some of the unique LNCRNAs discovered in this work had a negative relationship with our risk model, indicated that they might be tumor suppressor genes KIRP patients. There have been few studies on them, indicating that further research is needed (Fig. 2e).The timeROC package was used to construct a comparable ROC curve to assess the accuracy of our model in predicting the survival outcomes of patients with the disease. The AUC prediction value of the unique IncRNAs signature was 1, 1, and 1, for 1, 2, and 3-year survival rates, respectively, where both areas under the curve (AUCs) were > 0.5, attesting to our model's accuracy in predicting the disease survival outcome (Fig. 2f). The CASC19, AC090197.1, AC099850.3, AL033397.2, LINC00462, and B3GALT1-AS1 were all found to be overexpressed in the high-risk group, suggesting that they could all be harmful to the prognosis of KIRP patients. The LNCTAM34A and AC024022.1, on the other hand, were found to be highly expressed in the low-risk group, implying that all of them could be tumor suppressor genes in KIRP patients. Because there have been few studies on these two LNCRNAs, this may be the direction of future research (Figs. 2g and Additional file 1: Table S5b). The COX analysis revealed that IncRNA signature (HR 1.280, 95CI 1.221–1.341), age (HR 1.024, 95CI 1.009–1.040), and tumor stage (HR 1.457, 95CI 1.203–1.765) were mostly independent prognostic variables for KIRP patients' OS (Fig. 3a, b). Figure 3c demonstrates the link between the LNCRNA and mRNA. Many genes were discovered to be linked to numerous LNCRNAs, and LNCRNAs were found to be associated with many genes, showing that gene and ferroptosis related LNCRNAs play diverse roles through multi-gene crossover, which might be synergistic or antagonistic. This presents possibilities for future research. The heatmap for the prognosis signature of ferroptosis-related LNCRNAs and clinicopathological manifestations were evaluated. These eight risks LNCRNAs are not significantly associated with age or gender but are strongly connected to T, N, M, and tumor stages, with the T stage being the most significant one. This implies that the clinical professionals might use the expression of these biomarkers in KIRP patients' tumor stages as early as feasible to anticipate patients' prognoses and change of treatment regimens as needed (Fig. 3d). The hybrid nomogram (Fig. 3e), which incorporated clinicopathological features as well as the novel ferroptosis-related LNCRNAs prognostic signature, were stable and accurate, and may be used in KIRP patient care.

### Gene set enrichment analysis

**Fig. 4:**
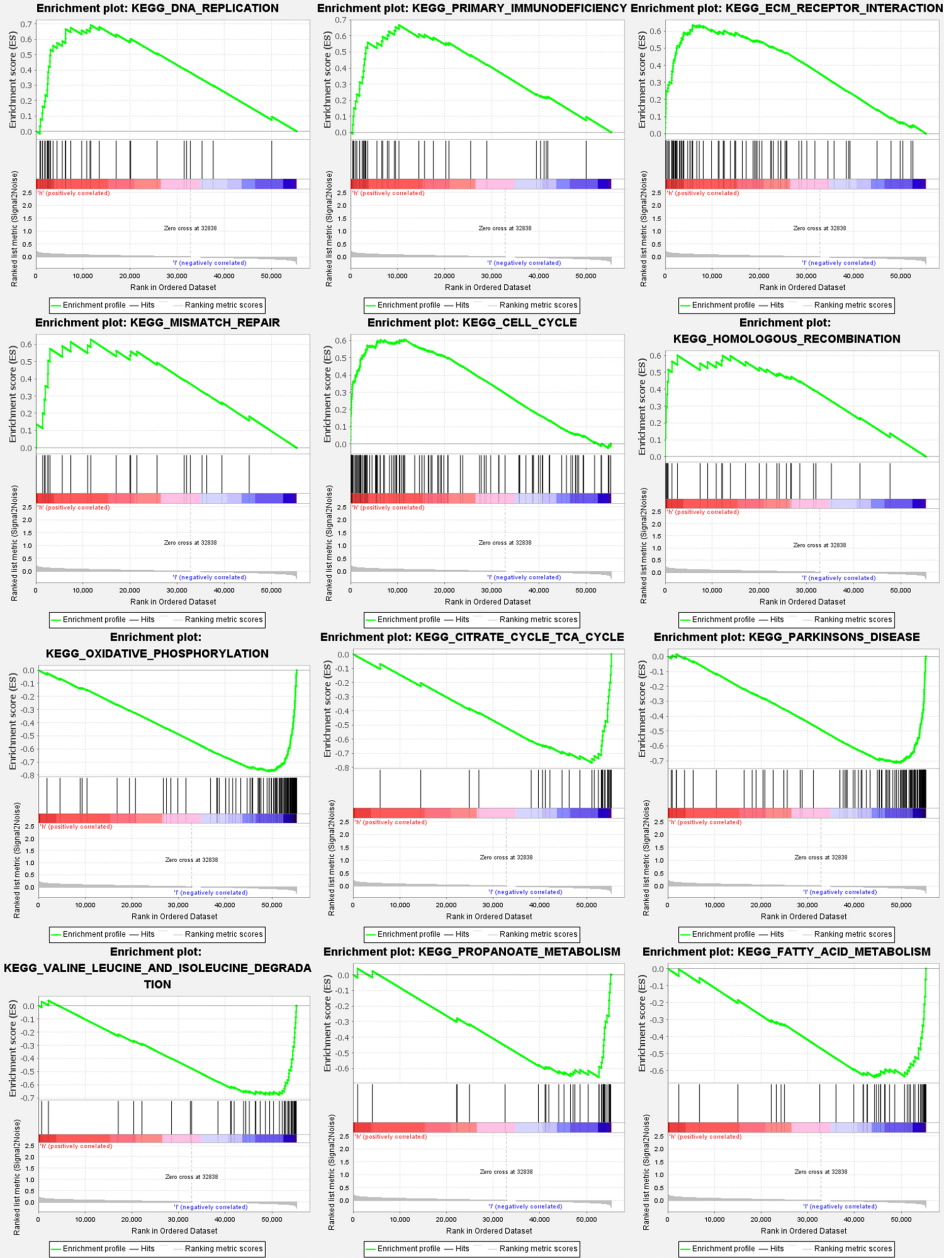
Gene set enrichment analyses. To clarify the difference of related functions or pathways in different samples, the top 6 enriched functions or pathways of each cluster were listed. The most enriched pathway was the P53 signaling pathway. Both FDR q-value and FWER *p* value were < 0.05

According to the GSEA, the majority of the new ferroptosis-related LNCRNAs prognostic signature controlled the immunological and tumor-related pathways such as DNA replication, primary immunodeficiency, ecm receptor interaction, mismatch repair, p53 signaling pathway, nod like receptor signaling pathway, etc. The top 6 enriched functions or pathways for each cluster are shown, (Fig. 4) and (Additional file 1: Table S6a–b). FDR q-value and FWER *p* value were both < 0.05. As a consequence, the “P53 SIGNALING PATHWAY” was the most enriched, and some of the genes were positively correlated with “H” or “L”.

### Immunity and gene expression

**Fig. 5:**
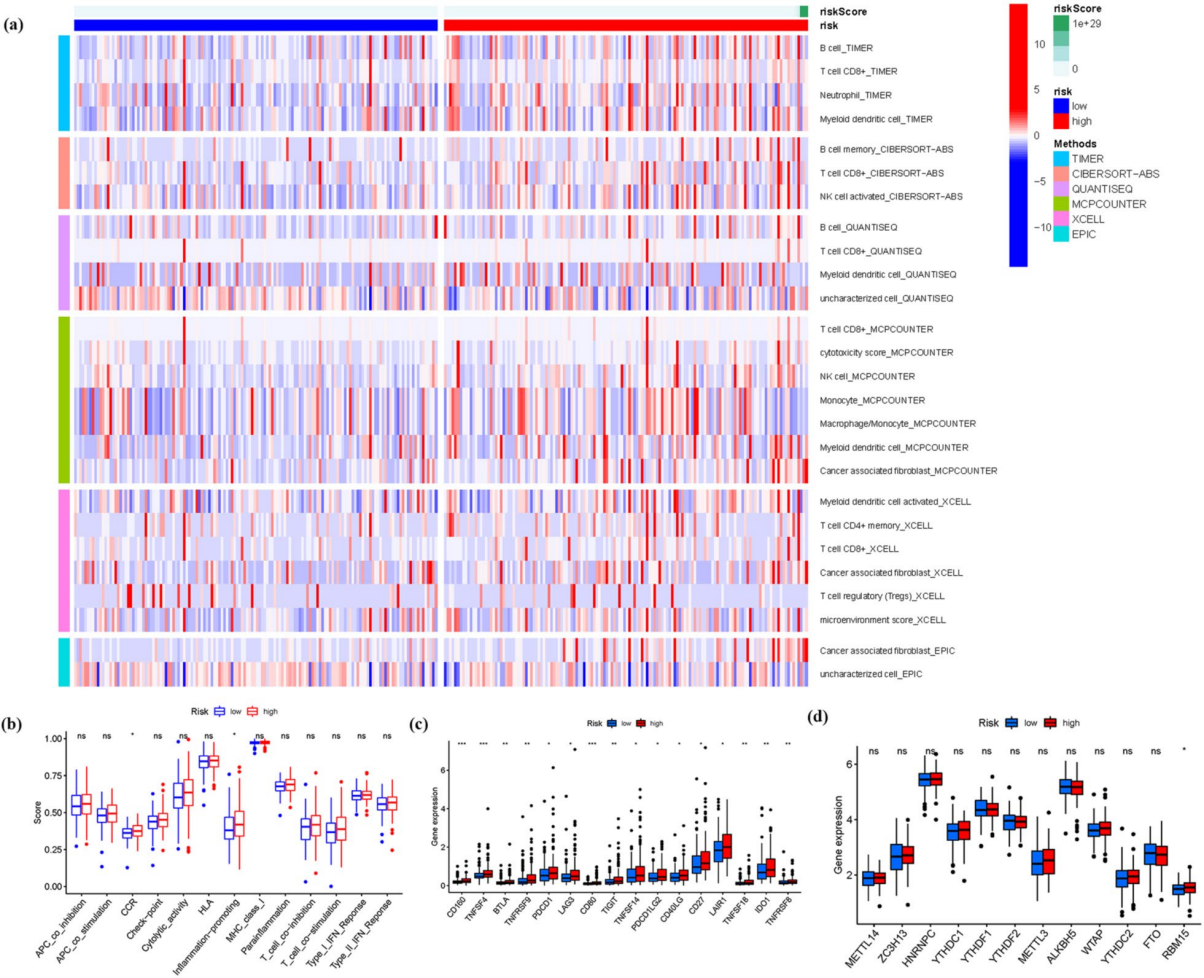
**a** A heatmap for immune responses. **b** ssGSEA for the association between immune cell subpopulations and related functions. **c** Immune checkpoint expression. **d** The expression of ferroptosis-related genes in KIRP risk groups

Based on CIBERSORT, ESTIMATE, MCPcounter, ssGSEA, and TIMER algorithms among high and low risk groups. Figure 5a demonstrates a heatmap of immunological responses generated using the CIBERSORT, ESTIMATE, MCP counter, single-sample gene set enrichment analysis (ssGSEA), and TIMER algorithms. In addition, considering the importance of the immune system in tumor pathogenesis, the ssGSEA enrichment analysis was used in investigating the relationship between the prognostic signature risk score and immune-related function type. Figure 5b shows that the score of immune function types, such as CCR and inflammation-promoting, were more significant in the high-risk group. However, the two immune-related functions only had one asterisk and a substantial p-value. Our findings provided essential information for tailoring treatment for KIRP patients with various risk ratings. Given the importance of checkpoint inhibitor-based immunotherapies, we looked at the changes in the immune checkpoint expression between the two groups. We identified a substantial difference in the expressions of CD160, TNFSF4, CD80, BTLA, TNFRSF9, and other genes between the two groups of patients (Fig. 5c). The expression of RBM15 was meaningful when ferroptosis-related mRNA expression was compared between the high and low risk groups (Fig. 5d). These findings suggested that RBM15 was not only involved in ferroptosis-related mRNA expression but also in the regulation of m6a, paving the way for future single-cell pan-cancer research.

## Discussions

Because of its advanced stage and dismal prognosis, treating KIRP is a serious clinical challenge [33]. The molecular determination of diagnostic biomarkers especially with KIRP, should always be highlighted. According to earlier studies, ferroptosis is involved in the pathological cell death associated with degenerative disorders, and overcoming malignant cell resistance to chemotherapy and increasing the removal of the defective cells [34, 35]. Ferroptosis offers the opportunity to act as a tumor suppressor, offering it as an alternative cancer therapy [36]. However, it is unclear how it impacts the KIRP formation through modulating the LNCRNA. This researcher investigated the role of immune infiltrating cells in the TME and immune checkpoint inhibitors in the KIRP prognosis. The findings of this study indicated a potential biomarker and therapeutic target.

In this study, the links between the ferroptosis-related gene expression and the LNCRNAs was explored utilizing the co-expression analysis. Using the co-expression network plot, we observed a phenomenon in which numerous LNCRNAs were connected to ferroptosis-related genes in the KIRP. Following that, we discovered 56 DEGs associated with ferroptosis. The KEGG analysis further revealed that the genes participated in chemical carcinogenesis-reactive oxygen species, HIF-1 signaling pathway, ferroptosis, and p53 signaling pathway. TAZ, a Hippo pathway effector, regulates ferroptosis in the renal cell carcinoma, influencing renal cancer cell development [37]. By generating ferroptosis, ART can prevent the progression of drug-resistant kidney cancer cells [38]. In tumor organoids and patient-derived xenografts with p53 mutations or deficiencies, ferroptosis inducers (FINs) that inhibit the SLC7A11 have a strong radio sensitizing effects [39]. Ferroptosis enhanced diabetic renal tubular injury in db mice through the HIF-1 pathway visibility [40]. Ferroptosis plays a key role in KIRP.

According to the risk score, the ferroptosis-related LNCRNAs were divided into high-risk and low-risk to investigate their probable functions in KIRP. The confidence interval and hazard ratio were identified using a data on prognosis-related LNCRNAs. Ferroptosis-related LNCRNAs appeared to be correlated with the prognosis of the KIRP in a university Cox regression study. This research discovered 8 ferroptosis-related LNCRNAs that have been connected to prognosis and have varied expression in high- and low-risk patients. Some LNCRNAs were overexpressed in high-risk individuals, while others were overexpressed in low-risk individuals (*P* < 0.05). We further investigated and examined the involvement of ferroptosis-related LNCRNAs in KIRP. Survival analysis based on the LNCRNA subtypes was used to evaluate the predictive importance of ferroptosis-related LNCRNAs. Patients with low-risk LNCRNAs had a better prognosis than those with high-risk LNCRNAs.

The CASC19, AC090197.1, AC099850.3, AL033397.2, LINC00462, and B3GALT1-AS1were found to be upregulated in the high-risk group, indicating that all these markers were involved in the malignancy processes for KIRP sufferers and were cancer-promoting factors. The findings of the above-mentioned biomarker suggest for future work, but the concrete evidence that will be responsible for the synthesis of important transcription factors associated with ferroptosis regulation, such as Fin56, NRF2, and SFRS9 is insufficient [41–43], and is lacking, necessitating further exploration. The LNCTAM34A and AC024022.1 were found to be upregulated in the low-risk group, these genes were KIRP tumor suppressor genes.

The LncRNA AC099850.3 promotes hepatocellular carcinoma proliferation and invasion through the PRR11/PI3K/AKT axis and is associated with patients’ prognosis [44]. Long non-coding RNA CASC19 facilitates non-small cell lung cancer cell proliferation and metastasis by targeting the miR-301b-3p/LDLR axis [45]. The LINC00462 promotes pancreatic cancer invasiveness through the miR-665/TGFBR1-TGFBR2/SMAD2/3 pathway [46]. According to Yujia Chen, through bioinformatics and vitro experiments, demonstrated that the LNCTAM34A promotes the proliferation, migration, and epithelial-mesenchymal transition of glioma cells [47]. According to Feilong Yang, AC024022.1 is found in the cytoplasm and is a predictive biomarker in papillary renal cell cancer [48]. Since these LNCRNAs were related with the malignancy processes in KIRP sufferers, these investigations demonstrated the validity and plausibility of our results. However, little study has been conducted on LNCRNA changes associated with ferroptosis. To understand the mechanism of ferroptosis-related LNCRNA alteration and identification, more study is needed to validate our findings.

We investigated and calculated the infiltration of various immune cells in the samples to assess the role of the immune cell infiltration and the TME plays in the KIRP. Depending on a study of immune cell infiltration disparities, the CCR and inflammation-promoting factors significantly infiltrated tumor tissues in high-risk patients. As a result, these cells’ invasion of the TME have a deleterious effect on the prognosis of KIRP patients. In ICI-resistant tumors, ferroptosis and immune checkpoint inhibitors (ICIs) work synergistically in boosting anticancer efficacy [49]. Only a little amount of study has been done on the link between the ICI and ferroptosis. In recent years, new ferroptosis-regulating factors have been discovered, including P53, ATF3/4, SLC7A11, ACSL4, and the BECN1 pathways. The LNCRNA is connected to the expression regulation of these factors [50], despite having little research on ferroptosis-related lnRNA and KIRP. Based on the evidences presented above, we concluded that a change in ferroptosis-related LNCRNAs is linked to the onset and progression of KIRP.

In GSEA, the p53-signaling pathway was found to be the most enriched pathway. Several investigations have indicated that p53 has a complex role in regulating ferroptosis caused by various inducers (FINs), with a promoter and anti-ferroptosis actions depending on the setting [51–53]. Guang Lei [39] discovered that the RT-induced ferroptosis is linked to p53 activation and improved clinical outcomes in cancer patients. It is hypothesized that the ferroptosis plays a crucial role in p53-mediated radio sensitization, and that FINs should be used in conjunction with RT to treat p53 mutant malignancy. Eszter Lajkó [54] provided evidences that target the GnRH receptor serves as a successful therapeutic approach in KIRP. Depending on the GnRH isoform and the presence of 4Lys(Bu), it regulated the expressions of several apoptosis-related genes, especially the TNF, TP53, and the members of growth-factor signaling. It has a strong inhibitory effect on the expression of growth-factor signaling elements, in which the upregulation of TP53 plays an important role. Taking the aforementioned characteristics into account, ferroptosis-related LNCRNAs influence KIRP cell migration and proliferation through influencing the P53 signaling pathway. In terms of survival, the low-risk subtype outperformed the high-risk subtype. The low-risk subtype exhibited a greater survival rate than the high-risk subtype, according to the ferroptosis-related LNCRNA prognostic model. In addition, our model has a high level of accuracy in predicting KIRP patient survival rate. A rise in the risk score is associated with an increase in death rates and the high-risk ratio. Our model had no effect on other clinical prognostic factors that influence patient survival outcomes. The principle is applied to a variety of clinical situations. Based on our findings and data from the literature, ferroptosis-related LNCRNAs were viable biomarkers in predicting KIRP patient survival outcomes.

There are always constraints despite the fact that our research gives theoretical foundations and research recommendations. Our analysis has limitations, we will be unable to obtain sufficient data sources from other publicly available sites to validate the model's trustworthiness and we also limited our initial expression to the signatures of eight risk ferroptosis-related LNCRNAs, with no additional functionality or fundamental analysis.

## Conclusions

In conclusion, we evaluated the expression patterns and clinical data of KIRP samples from the TCGA database to look for prognosis-related ferroptosis-related LNCRNAs. As part of the ferroptosis regulation, eight ferroptosis-related predictive LNCRNAs were identified in 298 KIRP patients. It has significant predictive value for KIRP. Our findings added to our understanding of ferroptosis-related LNCRNAs and immune cell infiltration in the TME, opening the path for future biomarkers and prognostic indicators. It is hoped that our findings will aid in the identification of ferroptosis-related LNCRNA that drives KIRP growth, allowing us to learn more about their role in the genesis and progression of KIRP cancers.

## Supplementary Information

**Additional file 1.** Ferroptosis-KIRC data.

## Data Availability

Patients who have provided informed consent for the use of their data have been included in the TCGA database, which is a public database. [TCGA] repository (https://portal.gdc.cancer.gov/). Users can freely obtain and publish appropriate articles based on the relevant data. Our study has no ethical difficulties or conflicts of interest because it is built on open-source data.

## Abbreviations

KIRP: Kidney renal papillary cell carcinoma
GO: Gene ontology
AUC: Areas under the curve
MF: Molecular functions
KEGG: Kyoto Encyclopedia of Genes and Genomes
ROC: Receiver-operating characteristics
GSEA: Gene set enrichment analyses
ICIs: Immune checkpoint inhibitors
RT: Radiotherapy
TME: Tumor microenvironment
TCGA: The Cancer Genome Atlas
LNCRNAs: Long non-coding RNAs
BP: Biological processes
CC: Cellular components
ROS: Reactive oxygen species
DCA: Decision curve analysis
FINs: Ferroptosis inducers
GnRH: Gonadotropin-releasing hormone
DEGs: Differentially expressed genes
OS: Overall survival
