# Supplementary material for: Potential biomarkers for predicting the overall survival outcome of kidney renal papillary cell carcinoma: an analysis of ferroptosis-related LNCRNAs
Source: BMC Urol. 2022 Sep 14;22:152. doi: 10.1186/s12894-022-01037-0 (PMC9476343; doi:10.1186/s12894-022-01037-0)
Supplement: Supplementary file 1 — Additional file 1. Ferroptosis-KIRC data. [file 12894_2022_1037_MOESM1_ESM.doc]

Additional file 1

Potential Biomarkers for Predicting the Overall Survival Outcome of Kidney Renal Papillary Cell Carcinoma: An analysis of Ferroptosis-Related LNCRNAs

Zixuan Wu^1,2^, Xuyan Huang^2^, Minjie Cai^2,3^, Peidong Huang^1^

**Supplementary appendix to the manuscript**

Contents of supplementary appendix

[Appendix 1 3](#__RefHeading___Toc400)

[382 ferroptosis-related genes 3](#__RefHeading___Toc22364)

[Table 1a. 150 ferroptosis-related genes of Driver. 3](#__RefHeading___Toc31473)

[Table 1b. 109 ferroptosis-related genes of suppressor. 28](#__RefHeading___Toc13046)

[Table 1c. 123 ferroptosis-related genes of marker. 51](#__RefHeading___Toc11653)

[Appendix 2 66](#__RefHeading___Toc29999)

[56 DEGs linked to ferroptosis 66](#__RefHeading___Toc23200)

[Table 2. 56 DEGs linked to ferroptosis. 66](#__RefHeading___Toc3504)

[Appendix 3 69](#__RefHeading___Toc16451)

[GO and KEGG enrichment analysis 69](#__RefHeading___Toc9076)

[Table 3a. GO enrichment analysis. 69](#__RefHeading___Toc11921)

[Table 3b. KEGG enrichment analysis. 102](#__RefHeading___Toc16091)

[Appendix 4 104](#__RefHeading___Toc18959)

[945 ferroptosis-related lncRNAs 104](#__RefHeading___Toc16750)

[Table 4. 945 ferroptosis-related lncRNAs 104](#__RefHeading___Toc26830)

[Appendix 5 126](#__RefHeading___Toc230)

[111 different lncRNAs 126](#__RefHeading___Toc10730)

[Table 5a. 111 different lncRNAs. 126](#__RefHeading___Toc18214)

[Table 5b. 8 lncRNAs with risk scores. 131](#__RefHeading___Toc8712)

[Appendix 6 151](#__RefHeading___Toc18251)

[gene set enrichment analyses (GSEA) 151](#__RefHeading___Toc3256)

[Table 6a. GSEA of high rish. 151](#__RefHeading___Toc22531)

[Table 6b. GSEA of low rish. 161](#__RefHeading___Toc12767)

# Appendix 1

**382 ferroptosis-related genes**

**Table 1a. 150 ferroptosis-related genes** of Driver.

| Symbol | Name | HGNC_ID | Evidence |
| --- | --- | --- | --- |
| RPL8 | Ribosomal protein L8 | HGNC:10368 | Required in erastin-induced ferroptosis. Silencing of it conferred against erastin-induced ferroptosis. |
| IREB2 | Iron response element binding protein 2 | HGNC:6115 | Required in erastin-induced ferroptosis. Silencing of it conferred against erastin-induced ferroptosis. |
| ATP5MC3 | ATP synthase membrane subunit c locus 3 | HGNC:843 | Required in erastin-induced ferroptosis. Silencing of it conferred against erastin-induced ferroptosis. |
| CS | Citrate synthase | HGNC:2422 | Required in erastin-induced ferroptosis. Silencing of it conferred against erastin-induced ferroptosis. |
| EMC2 | ER membrane protein complex subunit 2 | HGNC:28963 | Required in erastin-induced ferroptosis. Silencing of it conferred against erastin-induced ferroptosis. |
| ACSF2 | Acyl-CoA synthetase family member 2 | HGNC:26101 | Required in erastin-induced ferroptosis. Silencing of it conferred against erastin-induced ferroptosis. |
| NOX1 | Nicotinamide adenine dinucleotide phosphate (NADPH) oxidase (NOX) 1 | HGNC:7889 | Suppresses erastin-induced ferroptosis when inhibited by inhibitor. |
| CYBB | Cytochrome b-245 beta chain | HGNC:2578 | Suppresses erastin-induced ferroptosis when inhibited by inhibitor. |
| NOX3 | Nicotinamide adenine dinucleotide phosphate (NADPH) oxidase (NOX) 3 | HGNC:7890 | Suppresses erastin-induced ferroptosis when inhibited by inhibitor. |
| NOX4 | Nicotinamide adenine dinucleotide phosphate (NADPH) oxidase (NOX) 4 | HGNC:7891 | Suppresses erastin-induced ferroptosis when inhibited by inhibitor. |
| NOX5 | Nicotinamide adenine dinucleotide phosphate (NADPH) oxidase (NOX) 5 | HGNC:14874 | Suppresses erastin-induced ferroptosis when inhibited by inhibitor. |
| DUOX1 | Dual oxidase 1 | HGNC:3062 | Suppresses erastin-induced ferroptosis when inhibited by inhibitor. |
| DUOX2 | Dual oxidase 2 | HGNC:13273 | Suppresses erastin-induced ferroptosis when inhibited by inhibitor. |
| G6PD | Glucose-6-phosphate dehydrogenase | HGNC:4057 | Required in erastin-induced ferroptosis. |
| PGD | Phosphoglycerate dehydrogenase | HGNC:8891 | Required in erastin-induced ferroptosis. |
| VDAC2 | Valtage-dependent anion channels 2 | HGNC:12672 | Required in erastin-induced ferroptosis. |
| PIK3CA | Phosphatidylinositol-4,5-bisphosphate 3-kinase catalytic subunit alpha | HGNC:8975 | Inhibitor blocked ferroptotic cell death. |
| FLT3 | Fms related tyrosine kinase 3 | HGNC:3765 | Inhibitor blocked ferroptotic cell death. |
| SCP2 | Sterol carrier protein 2 | HGNC:10606 | SCP-2 inhibitors showed anti-ferroptotic activity, which was substantiated by knocking down SCP-2 in Gpx4−/− cells. |
| TP53 | Tumor protein p53 | HGNC:11998 | Inhibits cystine uptake and sensitizes cells to ferroptosis. Erastin induced high levels of cell death in p53+/+ MEFs. |
| ACSL4 | Acyl-CoA synthetase long chain family member 4 | HGNC:3571 | Deletion of this gene likely suppress ferroptosis by limiting the membrane-resident pool of oxidation-sensitive fatty acids. |
| LPCAT3 | Lysophosphatidylcholine acyltransferase 3 | HGNC:30244 | Deletion of this gene likely suppress ferroptosis by limiting the membrane-resident pool of oxidation-sensitive fatty acids. |
| NRAS | NRAS proto-oncogene, GTPase | HGNC:7989 | NRAS12V mutant protects RMS13 cells from ferroptotic cell death. |
| KRAS | KRAS proto-oncogene, GTPase | HGNC:6407 | KRAS12V mutant protects RMS13 cells from ferroptotic cell death. |
| HRAS | HRas proto-oncogene, GTPase | HGNC:5173 | HRAS12V mutant protects RMS13 cells from ferroptotic cell death. |
| TF | Transferrin | HGNC:11740 | Essential for the induction of ferroptotic cell death. Transferrin can only interact with transferrin receptor and be transported into the cell when it is loaded with iron. |
| TFRC | Transferrin receptor | HGNC:11763 | RNAi of transferrin receptor (TfR) inhibited ferroptosis. |
| TFR2 | Transferrin receptor 2 | HGNC:11762 | RNAi of transferrin receptor (TfR) inhibited ferroptosis. |
| SLC38A1 | Solute carrier family 38 member 1 | HGNC:13447 | RNAi knockdown markedly blocked ferroptosis. |
| SLC1A5 | Solute carrier family 1 member 5 | HGNC:10943 | Pharmacological inhibition by L-g-glutamyl-p-nitroanilide or RNAi knockdown markedly blocked ferroptosis. |
| GLS2 | Glutaminase 2 | HGNC:29570 | Both inhibitor inhibition and gene knockdown inhibit ferroptotic cell death. |
| GOT1 | Glutamic-oxaloacetic transaminase 1 | HGNC:4432 | RNAi reduced ferroptosis. |
| CARS1 | Cysteinyl-tRNA synthetase 1 | HGNC:1493 | Required for ferroptosis in diverse cell contexts. Knockdown of CARS inhibited erastin-induced death by preventing the induction of lipid reactive oxygen species, without altering iron homeostasis. |
| TP53 | Tumor protein p53 | HGNC:11998 | Wild type p53 can induce ferroptosis upon reactive oxygen species (ROS)-induced stress. |
| ALOX5 | Arachidonate 5-lipoxygenase | HGNC:435 | The 5-Lipoxygenase inhibitor zileuton protected HT22 neuronal cells from erastin-induced ferroptosis. |
| KEAP1 | Kelch like ECH associated protein 1 | HGNC:23177 | Knockdown of Keap1 reversed loss of p62-increased degradation of NRF2 in ferroptosis. Keap1 knockdown led to resistance to erastin-induced and sorafenib-induced growth inhibition with decreased ferroptotic events in the absence or presence of p62 knockdown. |
| HMOX1 | Heme oxygenase 1 | HGNC:5013 | Zinc protoporphyrin IX, a HO-1 inhibitor, prevented Erastin-triggered ferroptotic cancer cell death. Overexpression accelerates erastin-induced cell death. |
| TP53 | Tumor protein p53 | HGNC:11998 | p53^3KR/3KR Xrcc4^-/- MEF cells are very sensitive to ferroptosis. Stabilized in the spleens of p53^3KR/3KR Xrcc4^-/- mice which enables to prevent the development of pro-B-cell lymphomas. |
| TP53 | Tumor protein p53 | HGNC:11998 | Incubation with erastin led to profound cell death in wild-type MEFs. |
| GLS2 | Glutaminase 2 | HGNC:29570 | Upregulated in erastin-treated wild-type MEFs, and silencing Gls2 exihibits cell death defect in wild-type MEFs treated with erastin. |
| ATG5 | Autophagy related 5 | HGNC:589 | Knockout or knockdown limited erastin-induced ferroptosis. |
| ATG7 | Autophagy related 7 | HGNC:16935 | Knockout or knockdown limited erastin-induced ferroptosis. |
| NCOA4 | Nuclear receptor coactivator 4 | HGNC:7671 | Inhibition suppressed ferroptosis, and overexpression promoted ferroptosis. |
| TF | Transferrin | HGNC:11740 | Involved in siramesine and lapatinib-induced ferroptotic cell death. Increased following treatment with lapatinib alone or in combination with siramesine. Knocking down of transferrin resulted in decreased cell death and ROS after treatment. |
| ALOX5 | Arachidonate 5-lipoxygenase | HGNC:435 | Silencing ALOX genes made cells resistant to ferroptosis. |
| ALOX12 | Arachidonate 12-lipoxygenase, 12S type | HGNC:429 | Silencing ALOX genes made cells resistant to ferroptosis. |
| ALOX12B | Arachidonate 12-lipoxygenase, 12R type | HGNC:430 | Silencing ALOX genes made cells resistant to ferroptosis. |
| ALOX15 | Arachidonate 15-lipoxygenase | HGNC:433 | Silencing ALOX genes made cells resistant to ferroptosis. |
| ALOX15B | Arachidonate 15-lipoxygenase type B | HGNC:434 | Silencing ALOX genes made cells resistant to ferroptosis. Erastin-induced cell death was rescued by silencing either ALOX15B or ALOXE3, which supported the hypothesis that lipoxygenases are required for ferroptosis. |
| ALOXE3 | Arachidonate lipoxygenase 3 | HGNC:13743 | Silencing ALOX genes made cells resistant to ferroptosis. Erastin-induced cell death was rescued by silencing either ALOX15B or ALOXE3, which supported the hypothesis that lipoxygenases are required for ferroptosis. |
| PHKG2 | Phosphorylase kinase catalytic subunit gamma 2 | HGNC:8931 | U-2-OS cells became resistant to erastin upon PHKG2 silencing. Erastin-treated HT-1080 cells were rescued by shPHKG2. |
| TFRC | Transferrin receptor | HGNC:11763 | The gene targets of the enriched shRNAs are potential genes that positively regulate ferroptosis. |
| ACO1 | Aconitase 1 | HGNC:117 | The gene targets of the enriched shRNAs are potential genes that positively regulate ferroptosis. |
| IREB2 | iron responsive element binding protein 2 | HGNC:6115 | The gene targets of the enriched shRNAs are potential genes that positively regulate ferroptosis. |
| SLC38A1 | Solute carrier family 38 member 1 | HGNC:13447 | The gene targets of the enriched shRNAs are potential genes that positively regulate ferroptosis. |
| GLS2 | Glutaminase 2 | HGNC:29570 | The gene targets of the enriched shRNAs are potential genes that positively regulate ferroptosis. |
| G6PDX | _NA_ | _NA_ | The gene targets of the enriched shRNAs are potential genes that positively regulate ferroptosis. |
| ULK1 | Unc-51 like autophagy activating kinase 1 | HGNC:12558 | Potential positive regulators of ferroptosis. Knockout of ULK1 led to significantly lower levels of erastin-induced ferroptosis in a dose- and time-dependent manner. |
| ATG3 | Autophagy related 3 | HGNC:20962 | Potential positive regulators of ferroptosis. Knockout of ATG3 greatly reduced the sensitivity of MEFs to ferropotosis, and reconstituting ATG3 back to these cells restored the ferroptosis sensitivity. |
| ATG4D | Autophagy related 4D cysteine peptidase | HGNC:20789 | Potential positive regulators of ferroptosis. |
| ATG5 | Autophagy related 5 | HGNC:589 | Potential positive regulators of ferroptosis. Knockout of ATG5 led to significantly lower levels of erastin-induced ferroptosis in a dose- and time-dependent manner. |
| BECN1 | Beclin 1 | HGNC:1034 | Potential positive regulators of ferroptosis. |
| MAP1LC3A | Microtubule associated protein 1 light chain 3 alpha | HGNC:6838 | Potential positive regulators of ferroptosis. |
| GABARAPL2 | GABA type A receptor associated protein like 2 | HGNC:13291 | Potential positive regulators of ferroptosis. |
| GABARAPL1 | GABA type A receptor associated protein like 1 | HGNC:4068 | Potential positive regulators of ferroptosis. |
| ATG16L1 | Autophagy related 16 like 1 | HGNC:21498 | Potential positive regulators of ferroptosis. |
| WIPI1 | WD repeat domain, phosphoinositide interacting 1 | HGNC:25471 | Potential positive regulators of ferroptosis. |
| WIPI2 | WD repeat domain, phosphoinositide interacting 2 | HGNC:32225 | Potential positive regulators of ferroptosis. |
| SNX4 | Sorting nexin 4 | HGNC:11175 | Potential positive regulators of ferroptosis. |
| ATG13 | Autophagy related 13 | HGNC:29091 | Knockout of ATG13 greatly reduced the sensitivity of MEFs to ferropotosis, and reconstituting ATG13 back to these cells restored the ferroptosis sensitivity. |
| ULK2 | Unc-51 like autophagy activating kinase 2 | HGNC:13480 | Knockout of ULK2 led to significantly lower levels of erastin-induced ferroptosis in a dose- and time-dependent manner. |
| NCOA4 | Nuclear receptor coactivator 4 | HGNC:7671 | Elimination of NCOA4 expression by RNAi knockdown significantly block ferroptosis |
| ACSL4 | Acyl-CoA synthetase long chain family member 4 | HGNC:3571 | Knockdown inhibited erastin-induced ferroptosis, whereas overexpression restored ferroptosis sensitization. |
| TP53 | Tumor protein p53 | HGNC:11998 | p53 acetylation has a critical role in ferroptotic responses. Simultaneous loss of K98/117/161/162 acetylations is unable to induce ferroptosis, and its ability to thwart cancer growth is also abrogated. |
| SAT1 | Spermidine/spermine N1-acetyltransferase 1 | HGNC:10540 | p53-mediated activation of SAT1 contributes to ferroptotic cell death in the presence of ROS stress. Knockdown of Sat1 partially rescued ROS-induced ferroptosis. |
| ALOX15 | Arachidonate 15-lipoxygenase | HGNC:433 | SAT1- and ROS-induced ferroptosis was completely abrogated by PD146176, an ALOX15-specific inhibitor. |
| ACSL4 | Acyl-CoA synthetase long chain family member 4 | HGNC:3571 | Inhibition of ACSL4 was effective in protecting against RSL3-induced cell death. Acsl4 KO cells are resistant to ferroptosis. |
| LPCAT3 | Lysophosphatidylcholine acyltransferase 3 | HGNC:30244 | Knockdown of Lpcat3 increased resistance to ferroptosis triggered by RSL3. |
| ALOX15 | Arachidonate 15-lipoxygenase | HGNC:433 | Liproxstatin-1 inhibited the 15-LOX enzymatic activity and suppressed ferroptosis. |
| ACSL4 | Acyl-CoA synthetase long chain family member 4 | HGNC:3571 | An essential proferroptotic gene. Re-expression of Flag-tagged human wild-type (WT) ACSL4 (ACSL4-Flag) in Acsl4 KO Pfa1 cells restored full sensitivity to ferroptosis induction. Inhibition showed significantly prolonged survival compared to vehicle-treated mice. |
| KEAP1 | Kelch like ECH associated protein 1 | HGNC:23177 | Keap 1 silencing decreased ferroptosis. |
| EGFR | Epidermal growth factor receptor | HGNC:3236 | Cell death in activated EGFR mutant cells occurs by ferroptosis. Inhibiting EGFR and MAPK signaling rescued cell viability following cystine withdrawal. |
| NOX4 | NADPH oxidase 4 | HGNC:7891 | Inhibition of NADPH oxidase 4 (NOX4) blocked ferroptosis. |
| MAPK3 | Mitogen-activated protein kinase 3 | HGNC:6877 | Inhibiting EGFR and MAPK signaling rescued cell viability following cystine withdrawal. |
| MAPK1 | Mitogen-activated protein kinase 1 | HGNC:6871 | Inhibiting EGFR and MAPK signaling rescued cell viability following cystine withdrawal. |
| BID | BH3 interacting domain death agonist | HGNC:1050 | BID deletion prevents erastin- and glutamate-induced cell death. BID inhibition inhibited erastin-induced ferroptosis. |
| ACSL4 | Acyl-CoA synthetase long chain family member 4 | HGNC:3571 | Knockout of Acsl4 in ferroptosis-sensitive cells conferred protection from erastin- and RSL3-induced cell death. |
| ZEB1 | Zinc finger E-box binding homeobox 1 | HGNC:11642 | Knockout of ZEB1 prevents cell death induced by GPX4 inhibition. |
| KEAP1 | Kelch like ECH associated protein 1 | HGNC:23177 | Keap1 inhibition promotes resistance to ferroptosis. |
| DPP4 | Dipeptidyl peptidase 4 | HGNC:3009 | Required for ferroptosis in TP53-deficient CRC cells. |
| ALOX15 | Arachidonate 15-lipoxygenase | HGNC:433 | Suppression of ferroptosis following ALOX15 silencing was detected in cancer cells. Cells with exogenous expression of ALOX15 had an increased cell death rate following RSL3 treatment. |
| ALOX12 | Arachidonate 12-lipoxygenase, 12S type | HGNC:429 | 12‐LOX inhibitors prevented cell death, whereas ALOX12 overexpression significantly enhanced cell death. ALOX12 expression was gradually elevated during the erastin or RSL3 treatments, and was stable in the late stage of ferroptosis. |
| CDKN2A | Cyclin dependent kinase inhibitor 2A | HGNC:1787 | Combination of ARF induction and ROS treatment induced ferroptotic cell death. Knockdown of endogenous ARF protected cells from ROS-induced cell death. |
| PEBP1 | Phosphatidylethanolamine binding protein 1 | HGNC:8630 | Elevated levels of PEBP1 resulted in increased sensitivity of HK2 cells to RSL3 whereas lowered contents of PEBP1 in HAEC and HT22 cells were associated with decreased sensitivity to ferroptosis. |
| SOCS1 | Suppressor of cytokine signaling 1 | HGNC:19383 | Expression of SOCS1 sensitized cells to ferroptosis inducer. This effect of SOCS1 was efficiently blocked by ferroptosis inhibitor. Expression of SOCS1 reduced the levels of GSH, explaining in part its ability to sensitize cells to ferroptosis. |
| CDO1 | Cysteine dioxygenase type 1 | HGNC:1795 | CDO1 suppression contributes to ferroptosis resistance. |
| MYB | MYB proto-oncogene, transcription factor | HGNC:7545 | Erastin-induced ferroptosis was restrained when c-Myb was suppressed. |
| HMOX1 | Heme oxygenase 1 | HGNC:5013 | Inhibiting HO-1 effectively attenuated BAY-induced ferroptotic cell death. Defective HO-1 expression significantly rescued cell survival suppressed by BAY. |
| MAPK8 | Mitogen-activated protein kinase 8 | HGNC:6881 | JNK1/2 inhibitors inhibited t-BHP-induced ferroptosis. t-BHP treatment significantly increased the protein expression of p-JNK. |
| MAPK9 | Mitogen-activated protein kinase 9 | HGNC:6886 | JNK1/2 inhibitors inhibited t-BHP-induced ferroptosis. t-BHP treatment significantly increased the protein expression of p-JNK. |
| MAPK1 | Mitogen-activated protein kinase 1 | HGNC:6871 | ERK1/2 inhibitors inhibited t-BHP-induced ferroptosis. t-BHP treatment significantly increased the protein expression of p-ERK. |
| MAPK3 | Mitogen-activated protein kinase 3 | HGNC:6877 | ERK1/2 inhibitors inhibited t-BHP-induced ferroptosis. t-BHP treatment significantly increased the protein expression of p-ERK. |
| SLC1A5 | Solute carrier family 1 member 5 | HGNC:10943 | Overexpression of SLC1A5 restored miR-137-mediated ferroptosis suppression. |
| CHAC1 | ChaC glutathione specific gamma-glutamylcyclotransferase 1 | HGNC:28680 | CHAC1 degradation of GSH might enhance cystine-starvation-induced cell death. |
| MAPK14 | Mitogen-activated protein kinase 14 | HGNC:6876 | Ferroptosis was blocked by inhibiting p38 MAPK activation. |
| LINC00472 | Long intergenic non-protein coding RNA 472 | HGNC:21380 | Increases erastin-induced growth inhibition, whereas depletion of P53RRA decreased erastin-induced growth inhibition. |
| NOX4 | NADPH oxidase 4 | HGNC:7891 | Activated Nox4 contributes to PAB-induced ferroptotic cell death. knockdown made cells resistant to PAB-induced death. |
| GOT1 | Glutamic-oxaloacetic transaminase 1 | HGNC:4432 | Overexpression of miR‐9 suppressed GOT1, which subsequently reduced ferroptosis. Overexpression of GOT1 restored miR‐9 mediated ferroptosis suppression. |
| BECN1 | Beclin 1 | HGNC:1034 | Knockdown inhibits ferroptosis. Overexpression increases ferroptotic cancer cell death. |
| PRKAA2 | Protein kinase AMP-activated catalytic subunit alpha 2 | HGNC:9377 | Inhibition of PRKAA/AMPKalpha diminishes ferroptosis. |
| PRKAA1 | Protein kinase AMP-activated catalytic subunit alpha 1 | HGNC:9376 | Inhibition of PRKAA/AMPKalpha diminishes ferroptosis. |
| ELAVL1 | ELAV like RNA binding protein 1 | HGNC:3312 | ELAVL1 siRNA led to ferroptosis resistance, whereas ELAVL1 plasmid contributed to classical ferroptotic events. |
| BAP1 | BRCA1 associated protein 1 | HGNC:950 | Suppresses SLC7A11-mediated cystine uptake and promotes ferroptosis. BAP1 mutants lose their abilities to repress SLC7A11 and to promote ferroptosis. |
| TP53 | Tumor protein p53 | HGNC:11998 | Facilitates ART-induced ferroptosis. Conversely, knockdown of P53 blocked ART-induced ferroptosis. |
| ABCC1 | ATP binding cassette subfamily C member 1 | HGNC:51 | Accelerates ferroptosis. Disruption of MRP1 inhibited ferroptosis potently. |
| ACSL4 | Acyl-CoA synthetase long chain family member 4 | HGNC:3571 | Inhibition suppresses ferroptosis. |
| MIR6852 | microRNA 6852 | HGNC:49993 | Promotes ferroptosis. Binds to LINC0033 and serves as a negative upstream regulator of CBS-mediated ferroptosis inhibition. |
| ACVR1B | Activin A receptor type 1B | HGNC:172 | Inhibition attenuated erastin-induced ferroptosis. |
| TGFBR1 | Transforming growth factor beta receptor 1 | HGNC:11772 | Inhibition attenuated erastin-induced ferroptosis. |
| BAP1 | BRCA1 associated protein 1 | HGNC:950 | Promotes ferroptosis induced by class I ferroptosis inducer. |
| EPAS1 | Endothelial PAS domain protein 1 | HGNC:3374 | A driver of ferroptosis susceptibility. Ablation reduced susceptibility to ferroptosis. |
| HILPDA | Hypoxia inducible lipid droplet associated | HGNC:28859 | Promotes ferroptosis sensitivity downstream of HIF-2alpha. |
| HIF1A | Hypoxia inducible factor 1 subunit alpha | HGNC:4910 | Re-sensitized HIF-2alpha-null cells to ferroptosis. Induce ferroptosis sensitivity in cancer cells. |
| ALOX12 | Arachidonate 12-lipoxygenase, 12S type | HGNC:429 | An essential factor of p53-dependent ferroptosis. Loss of one Alox12 allele is sufficient to abrogate p53-mediated ferroptosis. |
| ACSL4 | Acyl-CoA synthetase long chain family member 4 | HGNC:3571 | Required for ferroptosis induced by erastin. ACSL4-null cells are resistant to ferroptosis induced by either erastin. |
| HMOX1 | Heme oxygenase 1 | HGNC:5013 | Enhances the ferroptotic process in PRDX6-silenced cells by promoting cellular accumulation of ferrous ions. Overexpression increases both erastin and RSL-3-induced lipid ROS. |
| IFNG | Interferon gamma | HGNC:5438 | Interferon gamma released from CD8+ T cells downregulates the expression of SLC3A2 and SLC7A11, and as a consequence, promotes tumour cell lipid peroxidation and ferroptosis. |
| ANO6 | Anoctamin 6 | HGNC:25240 | Essential for ferroptosis. Inhibition blocked ferroptotic cell death induced by RSL3/erastin. |
| LPIN1 | Lipin 1 | HGNC:13345 | Overexpression of adipose lipin‐1 in mice facilitated the onset of hepatic ferroptosis. |
| HMGB1 | High mobility group box 1 | HGNC:4983 | Required for erastin-induced ferroptosis. Knockdown of HMGB1 decreased erastin-induced cell death. |
| TNFAIP3 | TNF alpha induced protein 3 | HGNC:11896 | Overexpression increased ROS generation and enhanced erastin-induced ferroptosis, whereas knockdown inhibited erastin-induced ferroptosis. |
| TLR4 | Toll like receptor 4 | HGNC:11850 | Knockdown inhibited ferroptosis. |
| NOX4 | NADPH oxidase 4 | HGNC:7891 | Knockdown inhibited ferroptosis. |
| ATF3 | Activating transcription factor 3 | HGNC:785 | Promotes ferroptosis induced by erastin. |
| ATM | ATM serine/threonine kinase | HGNC:795 | Essential for ferroptosis. Genetic knockdown and chemical inhibition of ATM both suppress ferroptotic cell death. |
| YY1AP1 | YY1 associated protein 1 | HGNC:30935 | Makes cells more sensitive to ferroptosis. Cells lacking YAP were no longer sensitised to ferroptosis. |
| EGLN2 | Egl-9 family hypoxia inducible factor 2 | HGNC:14660 | Inhibiting EGLN2 activation diminished ferroptotic tumor cell death. |
| MIOX | Myo-inositol oxygenase | HGNC:14522 | Overexpression exacerbates cell death, knockdown inhibits ferroptosis. |
| TAZ | Tafazzin | HGNC:11577 | TAZ removal confers ferroptosis resistance, whereas overexpression of TAZS89A sensitizes cells to ferroptosis. |
| MTDH | Metadherin | HGNC:29608 | Can enhance sensitivity to inducers of ferroptosis. Enhances the vulnerability of cancer cells to ferroptosis. |
| IDH1 | Isocitrate dehydrogenase (NADP(+)) 1 | HGNC:5382 | Deletion of the mutant IDH1 allele or pharmacological inhibition of mutant IDH1 confers resistance to erastin-induced ferroptosis. Ectopic expression of mutant IDH1 promotes ferroptosis. |
| SIRT1 | Sirtuin 1 | HGNC:14929 | Knockout partially mitigates ferroptosis. |
| TAZ | Tafazzin | HGNC:11577 | TAZ removal confers ferroptosis resistance, while TAZS89A overexpression sensitizes cells to ferroptosis. |
| BECN1 | Beclin 1 | HGNC:1034 | Overexpression aggravated isoflurane-induced cell damage by upregulating ferroptosis. This phenomenon was significantly attenuated by silencing of Beclin1. |
| FBXW7 | F-box and WD repeat domain containing 7 | HGNC:16712 | FBXW7 plasmid induces ferroptosis. |
| PANX1 | Pannexin 1 | HGNC:8599 | Deletion protects against ferroptotic cell death. Silenced Panx1 expression significantly attenuated ferroptotic lipid peroxidation and iron accumulation induced by the ferroptosis inducer erastin. |
| DNAJB6 | DnaJ heat shock protein family (Hsp40) member B6 | HGNC:14888 | Promotes ferroptosis in esophageal squamous cell carcinoma. |
| BACH1 | BTB domain and CNC homolog 1 | HGNC:935 | Promotes ferroptosis by repressing the transcription of a subset of the erastin-induced protective genes. |
| ACSL4 | Acyl-CoA synthetase long chain family member 4 | HGNC:3571 | Overexpression induced ferroptosis. The opposite results were observed when ACSL4 was silenced. |
| LONP1 | Lon peptidase 1, mitochondrial | HGNC:9479 | Inhibition of LONP1 negatively regulates erastin-induced cell death. |

**Table 1b. 109 ferroptosis-related genes of suppressor.**

| Symbol | Name | HGNC_ID | Evidence |
| --- | --- | --- | --- |
| SLC7A11 | Solute carrier family 7 member 11 | HGNC:11059 | Silencing of SLC7A11 sensitized HT-1080 cells to erastin-induced death, whereas transfection of HT-1080 cells with a plasmid encoding SLC7A11 conferred protection from erastin- and sulfasalazine-induced death. |
| GPX4 | Glutathione peroxidase 4 | HGNC:4556 | RNAi-mediated GPX4 knockdown induces ferroptosis. |
| AKR1C1 | Aldo-keto reductase family 1 member C1 | HGNC:384 | Up-regulated in DU-145 erastin-resistant clones. Participate in the detoxification of toxic lipid metabolites. May confer partial resistance to erastin by enhancing the detoxification of reactive aldehydes generated downstream of the oxidative destruction of the plasma membrane during ferroptosis. |
| AKR1C2 | Aldo-keto reductase family 1 member C2 | HGNC:385 | Up-regulated in DU-145 erastin-resistant clones. Participate in the detoxification of toxic lipid metabolites. May confer partial resistance to erastin by enhancing the detoxification of reactive aldehydes generated downstream of the oxidative destruction of the plasma membrane during ferroptosis. |
| AKR1C3 | Aldo-keto reductase family 1 member C3 | HGNC:386 | Up-regulated in DU-145 erastin-resistant clones. Participate in the detoxification of toxic lipid metabolites. May confer partial resistance to erastin by enhancing the detoxification of reactive aldehydes generated downstream of the oxidative destruction of the plasma membrane during ferroptosis. |
| GPX4 | Glutathione peroxidase 4 | HGNC:4556 | Knockout of glutathione peroxidase 4 (Gpx4) causes cell death in a pathologically relevant form of ferroptosis. Knockdown renders cells more sensitive to ferroptosis-inducing agents. |
| RB1 | RB transcriptional corepressor 1 | HGNC:9884 | Rb knock-down cells exposed to sorafenib encounter ferroptosis. Lack of Rb sensitized HCC cells to the induction of ferroptosis. |
| HSPB1 | Heat shock protein family B (small) member 1 | HGNC:5246 | Knockdown of HSF1 and HSPB1 enhances erastin-induced ferroptosis, whereas heat shock pretreatment and overexpression of HSPB1 inhibits erastin-induced ferroptosis. |
| HSF1 | Heat shock transcription factor 1 | HGNC:5224 | Knockdown of HSF1 and HSPB1 enhances erastin-induced ferroptosis, whereas heat shock pretreatment and overexpression of HSPB1 inhibits erastin-induced ferroptosis. |
| SLC7A11 | Solute carrier family 7 member 11 | HGNC:11059 | Overexpressed in human cancer specimens. Overexpression inhibits ROS-induced ferroptosis. |
| GPX4 | Glutathione peroxidase 4 | HGNC:4556 | Ex vivo, Gpx4-deficient T cells rapidly accumulated membrane lipid peroxides and concomitantly underwent cell death driven by ferroptosis. |
| GCLC | Glutamate-cysteine ligase catalytic subunit | HGNC:4311 | RNAi knockdown sensitized cell death induced by cystine starvation. |
| SLC7A11 | Solute carrier family 7 member 11 | HGNC:11059 | Overexpression of SLC7A11 considerably abrogated ferroptosis. |
| NFE2L2 | Nuclear factor, erythroid 2 like 2 | HGNC:7782 | NRF2 plays a central role in protecting hepatocellular carcinoma (HCC) cells against ferroptosis |
| SQSTM1 | Sequestosome 1 | HGNC:11280 | The interaction between p62 and Keap1 increased following erastin and sorafenib treatment. Knockdown of p62 suppressed NRF2 expression and promoted growth inhibition with increased ferroptotic events including GSH depletion, lipid ROS production, and an increase of iron levels. |
| NQO1 | NAD(P)H quinone dehydrogenase 1 | HGNC:2874 | Knockdown of p62, quinone oxidoreductase‐1, heme oxygenase‐1, and ferritin heavy chain‐1 by RNA interference in HCC cells promoted ferroptosis in response to erastin and sorafenib. |
| HMOX1 | Heme oxygenase 1 | HGNC:5013 | Knockdown of p62, quinone oxidoreductase‐1, heme oxygenase‐1, and ferritin heavy chain‐1 by RNA interference in HCC cells promoted ferroptosis in response to erastin and sorafenib. |
| FTH1 | Ferritin heavy chain 1 | HGNC:3976 | Knockdown of p62, quinone oxidoreductase‐1, heme oxygenase‐1, and ferritin heavy chain‐1 by RNA interference in HCC cells promoted ferroptosis in response to erastin and sorafenib. |
| MUC1 | Mucin 1, cell surface associated | HGNC:7508 | MUC1-C (C-terminal subunit) blocks erastin-induced ferroptosis and induces increases in GSH. |
| SLC3A2 | Solute carrier family 3 member 2 | HGNC:11026 | Required for in vitro cell survival because of its role in protecting cells from ferroptosis. |
| MT1G | Metallothionein 1G | HGNC:7399 | A negative regulator of ferroptosis in HCC cells. Knockdown of MT‐1G by RNA interference increases glutathione depletion and lipid peroxidation, which contributes to sorafenib‐induced ferroptosis. |
| NFE2L2 | Nuclear factor, erythroid 2 like 2 | HGNC:7782 | Required for sorafenib‐induced expression of MT‐1G which is a ferroptosis suppressor. |
| SLC40A1 | Solute carrier family 40 member 1 | HGNC:10909 | Involved in siramesine and lapatinib-induced ferroptotic cell death. Its expression is decreased after treatment with siramesine alone or in combination with lapatinib. Overexpression FPN resulted in decreased ROS and cell death whereas knockdown of FPN increased cell death after siramesine and lapatinib treatment. |
| SLC7A11 | Solute carrier family 7 member 11 | HGNC:11059 | Knockdown increased cell death. |
| GPX4 | Glutathione peroxidase 4 | HGNC:4556 | Knockdown increased cell death. |
| SLC7A11 | Solute carrier family 7 member 11 | HGNC:11059 | Inhibition induces ferroptosis. Silencing of the SLC7A11 gene increases the cisplatin sensitivity of resistant HNC cells. |
| CISD1 | CDGSH iron sulfur domain 1 | HGNC:30880 | Genetic inhibition of CISD1 contributes to erastin-induced ferroptosis. Stabilization of the iron sulfur cluster of CISD1 inhibits ferroptosis. |
| SLC7A11 | Solute carrier family 7 member 11 | HGNC:11059 | Elevated levels of expression are resistant to erastin-induced ferroptosis. Repression of SLC7A11 expression by p53 sensitized cells to undergo erastin-induced ferroptosis. |
| FANCD2 | FA complementation group D2 | HGNC:3585 | Inhibits erastin-induced ferroptosis. Plays a novel role in the negative regulation of ferroptosis. |
| GPX4 | Glutathione peroxidase 4 | HGNC:4556 | Protects lipid peroxidation. Cell damage induced by GPx4 ablation is involved in ferroptosis. |
| NFE2L2 | Nuclear factor, erythroid 2 like 2 | HGNC:7782 | Nrf2 activation contributes to the resistance of HNCs to artesunate-induced ferroptosis. Nrf2 inhibition sensitizes head and neck cancer cells to artesunate-induced ferroptosis. |
| FTMT | Ferritin mitochondrial | HGNC:17345 | Overexpression significantly inhibited erastin-induced ferroptosis. |
| HSPA5 | Heat shock protein family A (Hsp70) member 5 | HGNC:5238 | Negatively regulates ferroptosis. Suppression of HSPA5 expression increased erastin-induced death. Overexpressed HSPA5 inhibited erastin-induced ferroptotic cell death. |
| ATF4 | Activating transcription factor 4 | HGNC:786 | Inhibition of ATF4 expression increased erastin-induced cell death. ATF4 results in the induction of HSPA5, which in turn protects against GPX4 protein degradation and subsequent ferroptosis. |
| SLC7A11 | Solute carrier family 7 member 11 | HGNC:11059 | Slc7a11 deletion increases susceptibility to iron overload-induced ferroptosis. |
| GPX4 | Glutathione peroxidase 4 | HGNC:4556 | Ferroptosis drives neurodegeneration in Gpx4BIKO mice. |
| GPX4 | Glutathione peroxidase 4 | HGNC:4556 | Downregulation conferred increased sensitivity to ferroptosis following cystine deprivation. |
| HMOX1 | Heme oxygenase 1 | HGNC:5013 | Demonsrates antiferroptotic role. HO-1 deficiency promotes erastin-induced ferroptosis. |
| ATF4 | Activating transcription factor 4 | HGNC:786 | ATF4 expression induces acquired cell death resistance. ATF4 knockdown renders cells susceptible for ferroptosis. |
| NFE2L2 | Nuclear factor, erythroid 2 like 2 | HGNC:7782 | Nrf2 over expression promotes resistance to ferroptosis. |
| TP53 | Tumor protein p53 | HGNC:11998 | Inhibits ferroptosis in human colorectal cancer (CRC) cells. Loss of TP53 restored erastin sensitivity. Inhibits cell death induction by erastin in human CRC cells. |
| SLC7A11 | Solute carrier family 7 member 11 | HGNC:11059 | Knockdown sensitized cells to erastin. |
| HELLS | Helicase, lymphoid specific | HGNC:4861 | LSH inhibits ferroptosis by decreasing the intracellular levels of iron and lipid ROS. |
| SCD | Stearoyl-CoA desaturase | HGNC:10571 | Depletion of the SCD1 and FADS2 metabolic genes induces ferroptosis. |
| FADS2 | Fatty acid desaturase 2 | HGNC:3575 | Depletion of the SCD1 and FADS2 metabolic genes induces ferroptosis. |
| SRC | SRC proto-oncogene, non-receptor tyrosine kinase | HGNC:11283 | Src-STAT3 activation renders the cell unable to undergo to ferroptosis. Src inhibition decreased cell viability significantly, and that loss of viability was rescued by ferroptosis inhibitors. |
| STAT3 | Signal transducer and activator of transcription 3 | HGNC:11364 | Src-STAT3 activation renders the cell unable to undergo to ferroptosis. |
| NFE2L2 | Nuclear factor, erythroid 2 like 2 | HGNC:7782 | ARF-mediated ferroptosis was largely abrogated by co-expression of NRF2. |
| PML | Promyelocytic leukemia | HGNC:9113 | PML expression turned cells highly resistant to ferroptosis. |
| MTOR | Mechanistic target of rapamycin kinase | HGNC:3942 | Necessary and sufficient to protect cardiomyocyte cells against ferroptotic cell death. mTOR overexpression suppressed ferroptotic cell death, whereas mTOR deletion exaggerated cell death. |
| NFS1 | NFS1 cysteine desulfurase | HGNC:15910 | Suppression of NFS1 cooperates with inhibition of cysteine transport to trigger ferroptosis in vitro and slow tumour growth. Suppression of NFS1 predisposes cancer cells to ferroptosis. |
| TP63 | Tumor protein p63 | HGNC:15979 | Delta Np63 alpha can inhibit ferroptosis independent of p53. Overexpression protects cells from ferroptosis-inducing agents. |
| SLC7A11 | Solute carrier family 7 member 11 | HGNC:11059 | Overexperession of SLC7A11 attenuated BAY-inhibited cell viability by ferroptosis. |
| TP53 | Tumor protein p53 | HGNC:11998 | p53 stabilization suppresses ferroptosis. p53 suppresses metabolic stress-induced ferroptosis. |
| CDKN1A | Cyclin dependent kinase inhibitor 1A | HGNC:1784 | Required to to suppress ferroptosis. |
| MIR137 | microRNA 137 | HGNC:31523 | Suppresses ferroptosis both in vitro and in vivo. |
| SLC40A1 | Solute carrier family 40 member 1 | HGNC:10909 | Overexpression of Fpn inhibited ferroptosis. |
| GPX4 | Glutathione peroxidase 4 | HGNC:4556 | Activation blocked ferroptosis. |
| GPX4 | Glutathione peroxidase 4 | HGNC:4556 | GPX4-overexpressing cells were resistant to reactive oxygen species-induced cell death. Conversely, GPX4-knockdown cells were sensitive to reactive oxygen species-induced cell death. |
| ENPP2 | Ectonucleotide pyrophosphatase/phosphodiesterase 2 | HGNC:3357 | Overexpression modestly promotes migration and proliferation and significantly inhibits erastin-induced ferroptosis. |
| VDAC2 | Voltage dependent anion channel 2 | HGNC:12672 | Overexpression could partially protect cells from ferroptosis. |
| FH | Fumarate hydratase | HGNC:3700 | FH inactivation (FH-/- ) proves synthetic lethal with inducers of ferroptosis. FH-/- sensitizes cells to multiple ferroptosis inducers. |
| CISD2 | CDGSH iron sulfur domain 2 | HGNC:24212 | Overexpression conferred resistance to ferroptosis. Inhibition blocked resistance to ferroptotic cell death. |
| SLC40A1 | Solute carrier family 40 member 1 | HGNC:10909 | A negative regulator of ferroptosis by reducing intracellular iron concentration. Knockdown accelerates erastin-induced ferroptosis. |
| MIR9-1 | microRNA 9-1 | HGNC:31641 | Overexpression of miR‐9 suppressed GOT1, which subsequently reduced ferroptosis. Suppression of miR‐9 increased the sensitivity of melanoma cells to ferroptosis inducers. |
| MIR9-2 | microRNA 9-2 | HGNC:31642 | Overexpression of miR‐9 suppressed GOT1, which subsequently reduced ferroptosis. Suppression of miR‐9 increased the sensitivity of melanoma cells to ferroptosis inducers. |
| MIR9-3 | microRNA 9-3 | HGNC:31646 | Overexpression of miR‐9 suppressed GOT1, which subsequently reduced ferroptosis. Suppression of miR‐9 increased the sensitivity of melanoma cells to ferroptosis inducers. |
| CBS | Cystathionine beta-synthase | HGNC:1550 | Inhibition triggers ferroptosis in hepatocellular carcinoma. |
| NFE2L2 | Nuclear factor, erythroid 2 like 2 | HGNC:7782 | Associated with resistance to ferroptosis. Inhibition of Nrf2 sensitized cells to ferroptosis. |
| SQSTM1 | Sequestosome 1 | HGNC:11280 | Inhibition of the p62 gene significantly reduced cell viability and increased cellular lipid ROS levels in HN3R cells; this was reversed by treatment with ferrostatin-1, a ferroptosis inducer. |
| GPX4 | Glutathione peroxidase 4 | HGNC:4556 | Overexpression GPX4 resulted in decreased cell death after RSL3 treatment. Moreover, this effect was able to be reversed by overexpression of GPX4. |
| ISCU | Iron-sulfur cluster assembly enzyme | HGNC:29882 | Over expression significantly attenuated DHA induced ferroptosis. |
| FTH1 | Ferritin heavy chain 1 | HGNC:3976 | FTH reconstituted cells exhibited the reduced lipid peroxides content and restored the DHA-induced ferroptosis. |
| ACSL3 | Acyl-CoA synthetase long chain family member 3 | HGNC:3570 | Required for exogenous monounsaturated fatty acids to protect cells against ferroptosis. Negatively correlates with ferroptosis sensitivity. |
| OTUB1 | OTU deubiquitinase, ubiquitin aldehyde binding 1 | HGNC:23077 | Inactivation promotes ferroptosis by down-regulating SLC7A11 levels. Overexpression is critical for tumor growth. |
| CD44 | CD44 molecule (Indian blood group) | HGNC:1681 | Knockdown sensitizes cells to ferroptosis. |
| LINC00336 | Long intergenic non-protein coding RNA 336 | HGNC:33813 | Overexpression inhibits ferroptosis. Knockdown promotes ferroptosis. |
| STAT3 | Signal transducer and activator of transcription 3 | HGNC:11364 | Upregulated in ferroptosis resistant cells. Inhibition increases ferroptosis. |
| BRD4 | Bromodomain containing 4 | HGNC:13575 | Inhibition induces ferroptosis. |
| PRDX6 | Peroxiredoxin 6 | HGNC:16753 | A negative regulator of ferroptotic cell death. |
| MIR17 | microRNA 17 | HGNC:31547 | Protects endothelial HUVEC cells from erastin-induced ferroptosis. Overexpression significantly reduced erastin-induced growth inhibition and ROS generation of HUVEC cells. |
| SCD | Stearoyl-CoA desaturase | HGNC:10571 | Inhibition of SCD1 induces ferroptotic cell death. Expression of SCD1 protects cells from ferroptosis. |
| SESN2 | Sestrin 2 | HGNC:20746 | Has cytoprotective effect against ferroptosis. In cells expressing Sesn2, erastin-induced cell death, ROS formation, and glutathione depletion were almost completely inhibited compared to that in control cells. |
| NF2 | Neurofibromin 2 | HGNC:7773 | Genetic inactivation of NF2 rendered cancer cells more sensitive to ferroptosis. Mediates cell density-dependent inhibition of ferroptosis. |
| ARNTL | Aryl hydrocarbon receptor nuclear translocator like | HGNC:701 | Degration of the protein is critical for ferroptosis. Blocking ARNTL degradation diminished ferroptotic tumor cell death. |
| HIF1A | Hypoxia inducible factor 1 subunit alpha | HGNC:4910 | Destabilizing HIF1A facilitated ferroptotic tumor cell death. |
| JUN | Jun proto-oncogene, AP-1 transcription factor subunit | HGNC:6204 | O-GlcNAcylated c-Jun represents an obstructive factor to ferroptosis. |
| CA9 | Carbonic anhydrase 9 | HGNC:1383 | Inhibition induces ferroptosis. |
| HSPA5 | Heat shock protein family A (Hsp70) member 5 | HGNC:5238 | Knockdown of GRP78 enhanced artesunate-induced ferroptosis of pancreatic cancer cells. |
| TMBIM4 | Transmembrane BAX inhibitor motif containing 4 | HGNC:24257 | Protects against ferroptosis in HCC cells. Inhibition increased ferroptotic cel death. |
| HSPA5 | Heat shock protein family A (Hsp70) member 5 | HGNC:5238 | Serves as a negative regulator of DHA-induced ferroptosis. |
| PLIN2 | Perilipin 2 | HGNC:248 | An indispensable gene and protein in the suppression of ferroptosis caused by abnormal lipometabolism in gastric carcinoma. |
| MIR212 | microRNA 212 | HGNC:31589 | Overexpression of miR-212-5p attenuated ferroptosis while downregulation of miR-212-5p promoted ferroptotic cell death. |
| Fer1HCH | Ferritin 1 Heavy Chain Homolog | _NA_ | Reduced heavy chain levels caused severe mitochondrial defects and ferroptosis. |
| AIFM2 | Apoptosis inducing factor mitochondria associated 2 | HGNC:21411 | A glutathione-independent ferroptosis suppressor. Pharmacological targeting of FSP1 strongly synergizes with GPX4 inhibitors to trigger ferroptosis. |
| AIFM2 | Apoptosis inducing factor mitochondria associated 2 | HGNC:21411 | A potent ferroptosis-resistance factor. Positively correlates with ferroptosis resistance. |
| LAMP2 | Lysosomal associated membrane protein 2 | HGNC:6501 | Knockdown promoted ferroptosis. |
| ZFP36 | ZFP36 ring finger protein | HGNC:12862 | ZFP36 plasmid impaired FBXW7 plasmid-induced HSC ferroptosis. Overexpression of Zfp36 impaired erastin- or sorafenib-induced ferroptosis. |
| GPX4 | Glutathione peroxidase 4 | HGNC:4556 | Depletion or inhibition resulted in cell death by ferroptosis. |
| PROM2 | Prominin 2 | HGNC:20685 | Induced by ferroptotic stress and promotes resistance to ferroptotic cell death. Facilitates ferroptosis resistance in mammary epithelial and breast carcinoma cells. |
| CHMP5 | Charged multivesicular body protein 5 | HGNC:26942 | Ferroptosis activators increase ESCRT-III subunits (e.g., CHMP5 and CHMP6). Knockdown of CHMP5 or CHMP6 sensitizes human cancer cells to ferroptosis. |
| CHMP6 | Charged multivesicular body protein 6 | HGNC:25675 | Ferroptosis activators increase ESCRT-III subunits (e.g., CHMP5 and CHMP6). Knockdown of CHMP5 or CHMP6 sensitizes human cancer cells to ferroptosis. |
| AKR1C1 | Aldo-keto reductase family 1 member C1 | HGNC:384 | Inhibition completely resensitizes resistant melanoma cells to ferroptosis execution. |
| AKR1C2 | Aldo-keto reductase family 1 member C2 | HGNC:385 | Inhibition completely resensitizes resistant melanoma cells to ferroptosis execution. |
| AKR1C3 | Aldo-keto reductase family 1 member C3 | HGNC:386 | Inhibition completely resensitizes resistant melanoma cells to ferroptosis execution. |
| CBS | Cystathionine beta-synthase | HGNC:1550 | Knockdown in erastin-resistant cells caused ferroptotic cell death, while overexpression conferred ferroptosis resistance. |
| NFE2L2 | Nuclear factor, erythroid 2 like 2 | HGNC:7782 | Genetically repression of NRF2 enhanced ferroptosis susceptibility. |
| CAV1 | Caveolin 1 | HGNC:1527 | Cav-1 deficiency aggravated ferroptosis. Short hairpin RNA of Cav-1 promoted ferroptosis, which was ameliorated by Cav-1 overexpression. |
| GCH1 | GTP cyclohydrolase 1 | HGNC:4193 | Gch1 overexpression and its downstream metabolites BH4/BH2 rescue from ferroptosis. Inhibition of GCH1 activity can sensitize resistant cancer cells to ferroptosis induction. |

**Table 1c. 123 ferroptosis-related genes** of marker.

| Symbol | Name | HGNC_ID | Evidence |
| --- | --- | --- | --- |
| PTGS2 | Prostaglandin-endoperoxide synthase 2 | HGNC:9605 | Simply a downstream marker of ferroptosis. The most upregulated gene in BJeLR cells upon treatment with either erastin or (1S, 3R)-RSL3, but ferroptotic cell death was not affected by inhibition of the enzyme. |
| DUSP1 | Dual specificity phosphatase 1 | HGNC:3064 | Expression was upregulated during ferroptosis induced by erastin or RSL3. |
| NOS2 | Nitric oxide synthase 2 | HGNC:7873 | Expression was upregulated during ferroptosis induced by erastin or RSL3. |
| NCF2 | Neutrophil cytosolic factor 2 | HGNC:7661 | Expression was upregulated during ferroptosis induced by erastin or RSL3. |
| MT3 | Metallothionein 3 | HGNC:7408 | Expression was upregulated during ferroptosis induced by erastin or RSL3. |
| UBC | Ubiquitin C | HGNC:12468 | Expression was upregulated during ferroptosis induced by erastin or RSL3. |
| ALB | Albumin | HGNC:399 | Expression was upregulated during ferroptosis induced by erastin or RSL3. |
| TXNRD1 | Thioredoxin reductase 1 | HGNC:12437 | Expression was upregulated during ferroptosis induced by erastin or RSL3. |
| SRXN1 | Sulfiredoxin 1 | HGNC:16132 | Expression was upregulated during ferroptosis induced by erastin or RSL3. |
| GPX2 | Glutathione peroxidase 2 | HGNC:4554 | Expression was upregulated during ferroptosis induced by erastin or RSL3. |
| BNIP3 | BCL2 interacting protein 3 | HGNC:1084 | Expression was upregulated during ferroptosis induced by erastin or RSL3. |
| OXSR1 | Oxidative stress responsive kinase 1 | HGNC:8508 | Expression was upregulated during ferroptosis induced by erastin or RSL3. |
| SELENOS | Selenoprotein S | HGNC:30396 | Expression was upregulated during ferroptosis induced by erastin or RSL3. |
| ANGPTL7 | Angiopoietin like 7 | HGNC:24078 | Expression was downregulated during ferroptosis induced by erastin or RSL3. |
| CHAC1 | ChaC glutathione specific gamma-glutamylcyclotransferase 1 | HGNC:28680 | Up-regulated in erastin-treated samples. A useful pharmacodynamic marker of system Xc- inhibition. |
| SLC7A11 | Solute carrier family 7 member 11 | HGNC:11059 | Similar to erastin treatment, silencing of this gene inhibits glutamate release. Erastin specifically inhibits SLC7A11-dependent system Xc- function. |
| DDIT4 | DNA damage inducible transcript 4 | HGNC:24944 | Up-regulated (>= 2 fold) in erastin-treated samples. |
| LOC284561 | _NA_ | _NA_ | Up-regulated (>= 2 fold) in erastin-treated samples. |
| ASNS | Asparagine synthetase (glutamine-hydrolyzing) | HGNC:753 | Up-regulated (>= 2 fold) in erastin-treated samples. |
| TSC22D3 | TSC22 domain family member 3 | HGNC:3051 | Up-regulated (>= 2 fold) in erastin-treated samples. |
| DDIT3 | DNA damage inducible transcript 3 | HGNC:2726 | Up-regulated (>= 2 fold) in erastin-treated samples. |
| JDP2 | Jun dimerization protein 2 | HGNC:17546 | Up-regulated (>= 2 fold) in erastin-treated samples. |
| SESN2 | Sestrin 2 | HGNC:20746 | Up-regulated (>= 2 fold) in erastin-treated samples. |
| SLC1A4 | Solute carrier family 1 member 4 | HGNC:10942 | Up-regulated (>= 2 fold) in erastin-treated samples. |
| PCK2 | Phosphoenolpyruvate carboxykinase 2, mitochondrial | HGNC:8725 | Up-regulated (>= 2 fold) in erastin-treated samples. |
| TXNIP | Thioredoxin interacting protein | HGNC:16952 | Up-regulated (>= 2 fold) in erastin-treated samples. |
| VLDLR | Very low density lipoprotein receptor | HGNC:12698 | Up-regulated (>= 2 fold) in erastin-treated samples. |
| GPT2 | Glutamic--pyruvic transaminase 2 | HGNC:18062 | Up-regulated (>= 2 fold) in erastin-treated samples. |
| PSAT1 | Phosphoserine aminotransferase 1 | HGNC:19129 | Up-regulated (>= 2 fold) in erastin-treated samples. |
| LURAP1L | Leucine rich adaptor protein 1 like | HGNC:31452 | Up-regulated (>= 2 fold) in erastin-treated samples. |
| SLC7A5 | Solute carrier family 7 member 5 | HGNC:11063 | Up-regulated (>= 2 fold) in erastin-treated samples. |
| HERPUD1 | Homocysteine inducible ER protein with ubiquitin like domain 1 | HGNC:13744 | Up-regulated (>= 2 fold) in erastin-treated samples. |
| XBP1 | X-box binding protein 1 | HGNC:12801 | Up-regulated (>= 2 fold) in erastin-treated samples. |
| ATF3 | Activating transcription factor 3 | HGNC:785 | Up-regulated (>= 2 fold) in erastin-treated samples. |
| SLC3A2 | Solute carrier family 3 member 2 | HGNC:11026 | Up-regulated (>= 2 fold) in erastin-treated samples. |
| CBS | Cystathionine beta-synthase | HGNC:1550 | Up-regulated (>= 2 fold) in erastin-treated samples. |
| ATF4 | Activating transcription factor 4 | HGNC:786 | Up-regulated (>= 2 fold) in erastin-treated samples. |
| ZNF419 | Zinc finger protein 419 | HGNC:20648 | Up-regulated (>= 2 fold) in erastin-treated samples. |
| KLHL24 | Kelch like family member 24 | HGNC:25947 | Up-regulated (>= 2 fold) in erastin-treated samples. |
| TRIB3 | Tribbles pseudokinase 3 | HGNC:16228 | Up-regulated (>= 2 fold) in erastin-treated samples. |
| ZFP69B | ZFP69 zinc finger protein B | HGNC:28053 | Up-regulated (>= 2 fold) in erastin-treated samples. |
| ATP6V1G2 | ATPase H+ transporting V1 subunit G2 | HGNC:862 | Up-regulated (>= 2 fold) in erastin-treated samples. |
| VEGFA | Vascular endothelial growth factor A | HGNC:12680 | Up-regulated (>= 2 fold) in erastin-treated samples. |
| GDF15 | Growth differentiation factor 15 | HGNC:30142 | Up-regulated (>= 2 fold) in erastin-treated samples. |
| TUBE1 | Tubulin epsilon 1 | HGNC:20775 | Up-regulated (>= 2 fold) in erastin-treated samples. |
| ARRDC3 | Arrestin domain containing 3 | HGNC:29263 | Up-regulated (>= 2 fold) in erastin-treated samples. |
| CEBPG | CCAAT enhancer binding protein gamma | HGNC:1837 | Up-regulated (>= 2 fold) in erastin-treated samples. |
| SNORA16A | Small nucleolar RNA, H/ACA box 16A | HGNC:32605 | Down-regulated (>= 2 fold) in erastin-treated samples. |
| RGS4 | Regulator of G protein signaling 4 | HGNC:10000 | Down-regulated (>= 2 fold) in erastin-treated samples. |
| BLOC1S5-TXNDC5 | BLOC1S5-TXNDC5 readthrough (NMD candidate) | HGNC:42001 | Down-regulated (>= 2 fold) in erastin-treated samples. |
| LOC390705 | _NA_ | _NA_ | Down-regulated (>= 2 fold) in erastin-treated samples. |
| EIF2S1 | Eukaryotic translation initiation factor 2 subunit 1 | HGNC:3265 | Phosphorylated in erastin-treated sample. |
| KIM-1 | Kidney injury molecule-1 | _NA_ | Down-regulated upon Fer-1 appearance |
| IL6 | Interleukin 6 | HGNC:6018 | Down-regulated upon Fer-1 appearance |
| CXCL2 | C-X-C motif chemokine ligand 2 | HGNC:4603 | Down-regulated upon Fer-1 appearance |
| RELA | RELA proto-oncogene, NF-kB subunit | HGNC:9955 | Down-regulated upon Fer-1 appearance |
| HSD17B11 | Hydroxysteroid 17-beta dehydrogenase 11 | HGNC:22960 | Enriched in RSL3-resistant cells. |
| AGPAT3 | 1-acylglycerol-3-phosphate O-acyltransferase 3 | HGNC:326 | Enriched in RSL3-resistant cells. |
| SETD1B | SET domain containing 1B, histone lysine methyltransferase | HGNC:29187 | Enriched in GPX4 inhibitor ML162-resistant cells. |
| HMOX1 | Heme oxygenase 1 | HGNC:5013 | Its expression increased in response to artesunate-induced ferroptosis, indicating activation of ROS-mediated signaling pathways. |
| TF | Transferrin | HGNC:11740 | Its expression is decreased in patients. |
| FTL | Ferritin light chain | HGNC:3999 | Its expression is decreased in patients. |
| RPL8 | Ribosomal protein L8 | HGNC:10368 | Significantly reduced in tumor tissues |
| ATP5MC3 | ATP synthase membrane subunit c locus 3 | HGNC:843 | Significantly reduced in tumor tissues |
| TFRC | Transferrin receptor | HGNC:11763 | Expression of this gene is increased in patients. |
| MAFG | MAF bZIP transcription factor G | HGNC:6781 | The interaction between NRF2 and MafG was increased in response to erastin and sorafenib. |
| IL33 | Interleukin 33 | HGNC:16028 | IL-33 upregulation is a feature of ferroptosis. Ferrostatin-1, an inhibitor of ferroptosis, prevented the upregulation of IL-33. |
| FTH1 | Ferritin heavy chain 1 | HGNC:3976 | An increase of endogenous FTH1 level during ferroptosis. Degradation of FTH1 protein upon ferroptosis induction. |
| SLC40A1 | Solute carrier family 40 member 1 | HGNC:10909 | Erastin-induced mRNA expression is upregulated in FANCD2-deficient cells. |
| TF | Transferrin | HGNC:11740 | Erastin-induced mRNA expression is upregulated in FANCD2-deficient cells. |
| TFRC | Transferrin receptor | HGNC:11763 | Erastin-induced mRNA expression is upregulated in FANCD2-deficient cells. |
| FTH1 | Ferritin heavy chain 1 | HGNC:3976 | Erastin-induced mRNA expression is downregulated in FANCD2-deficient cells. |
| GPX4 | Glutathione peroxidase 4 | HGNC:4556 | Erastin-induced mRNA expression is downregulated in FANCD2-deficient cells. |
| HAMP | Hepcidin antimicrobial peptide | HGNC:15598 | Erastin-induced mRNA expression is downregulated in FANCD2-deficient cells. |
| HSPB1 | Heat shock protein family B (small) member 1 | HGNC:5246 | Erastin-induced mRNA expression is downregulated in FANCD2-deficient cells. |
| NFE2L2 | Nuclear factor, erythroid 2 like 2 | HGNC:7782 | Erastin-induced mRNA expression is downregulated in FANCD2-deficient cells. |
| STEAP3 | STEAP3 metalloreductase | HGNC:24592 | Erastin-induced mRNA expression is downregulated in FANCD2-deficient cells. |
| DRD5 | Dopamine receptor D5 | HGNC:3026 | Ferroptotic erastin induces DRD5 gene expression in ferroptosis. |
| GPX4 | Glutathione peroxidase 4 | HGNC:4556 | Erastin promoted GPX4 degradation. Antiferroptotic dopamine increased the protein stability of glutathione peroxidase 4. |
| DRD4 | Dopamine receptor D4 | HGNC:3025 | Antiferroptotic dopamine suppressed dopamine receptor D4 protein degradation. Ferroptotic erastin promotes DRD4 protein degradation. |
| MAP3K5 | Mitogen-activated protein kinase kinase kinase 5 | HGNC:6857 | Cold stress evokes ferroptosis, and the ASK1‐p38 pathway is activated downstream of lipid peroxide, leading to the cell death. ASK1‐p38 axis is also activated in the erastin‐induced ferroptosis model. |
| MAPK14 | Mitogen-activated protein kinase 14 | HGNC:6876 | Cold stress evokes ferroptosis, and the ASK1‐p38 pathway is activated downstream of lipid peroxide, leading to the cell death. ASK1‐p38 axis is also activated in the erastin‐induced ferroptosis model. |
| SLC2A1 | Solute carrier family 2 member 1 | HGNC:11005 | Increased at LSH overexpression. Decreased at LSH knockdown. LSH can inhibit ferroptosis. |
| SLC2A3 | Solute carrier family 2 member 3 | HGNC:11007 | Decreased at LSH knockdown. LSH can inhibit ferroptosis. |
| SLC2A6 | Solute carrier family 2 member 6 | HGNC:11011 | Increased at LSH overexpression. Decreased at LSH knockdown. LSH can inhibit ferroptosis. |
| SLC2A8 | Solute carrier family 2 member 8 | HGNC:13812 | Decreased at LSH knockdown. LSH can inhibit ferroptosis. |
| SLC2A12 | Solute carrier family 2 member 12 | HGNC:18067 | Increased at LSH overexpression. Decreased at LSH knockdown. LSH can inhibit ferroptosis. |
| GLUT13 | _NA_ | _NA_ | Increased at LSH overexpression. Decreased at LSH knockdown. LSH can inhibit ferroptosis. |
| SLC2A14 | Solute carrier family 2 member 14 | HGNC:18301 | Decreased at LSH knockdown. LSH can inhibit ferroptosis. |
| EIF2AK4 | Eukaryotic translation initiation factor 2 alpha kinase 4 | HGNC:19687 | CHAC1 degradation of GSH enhances cystine-starvation-induced ferroptosis through the activated GCN2-eIF2 alpha-ATF4 pathway. |
| EIF2S1 | Eukaryotic translation initiation factor 2 subunit alpha | HGNC:3265 | CHAC1 degradation of GSH enhances cystine-starvation-induced ferroptosis through the activated GCN2-eIF2 alpha-ATF4 pathway. |
| ATF4 | Activating transcription factor 4 | HGNC:786 | CHAC1 degradation of GSH enhances cystine-starvation-induced ferroptosis through the activated GCN2-eIF2 alpha-ATF4 pathway. |
| ALOX5 | Arachidonate 5-lipoxygenase | HGNC:435 | Overexpression sensitizes cells to ferroptosis. |
| ALOX12 | Arachidonate 12-lipoxygenase, 12S type | HGNC:429 | Overexpression sensitizes cells to ferroptosis. |
| ALOX15 | Arachidonate 15-lipoxygenase | HGNC:433 | Overexpression sensitizes cells to ferroptosis. |
| ALOX5 | Arachidonate 5-lipoxygenase | HGNC:435 | Necessary for hemin‐induced ferroptosis in vitro. |
| ACSF2 | Acyl-CoA synthetase family member 2 | HGNC:26101 | Increased in ferroptotic events. Decreased in DFO-induced resistance to ferroptosis. |
| IREB2 | Iron responsive element binding protein 2 | HGNC:6115 | Increased in ferroptotic events. Decreased in DFO-induced resistance to ferroptosis. |
| GPX4 | Glutathione peroxidase 4 | HGNC:4556 | Upregulated in DFO-induced resistance to ferroptosis. |
| HMGB1 | High mobility group box 1 | HGNC:4983 | Associated with ferroptotic cell death. Ferroptosis activators induce HMGB1 release. |
| HMOX1 | Heme oxygenase 1 | HGNC:5013 | Required for DOX-induced ferroptosis. |
| NFE2L2 | Nuclear factor, erythroid 2 like 2 | HGNC:7782 | DOX treatment induced ferroptosis. Protein and mRNA levels of Nrf2 were increased after DOX treatment. |
| ELAVL1 | ELAV like RNA binding protein 1 | HGNC:3312 | Binds to and increases the expression of the negative ferroptosis regulator LINC00336. |
| SLC3A2 | Solute carrier family 3 member 2 | HGNC:11026 | Strongly correlated with resistance to ferroptosis inducers. |
| SLC7A11 | Solute carrier family 7 member 11 | HGNC:11059 | Strongly correlated with resistance to ferroptosis inducers. |
| TFAP2C | Transcription factor AP-2 gamma | HGNC:11744 | Activated by ferroptosis inhibitor selenium. |
| SP1 | Sp1 transcription factor | HGNC:11205 | Activated by ferroptosis inhibitor selenium. |
| HBA1 | Hemoglobin subunit alpha 1 | HGNC:4823 | Upregulated in cells treated with ferroptosis inducer erastin. Stimulates ferroptosis possibly in a GSH-dependent manner. |
| NNMT | Nicotinamide N-methyltransferase | HGNC:7861 | Upregulated in cells treated with ferroptosis inducer erastin. Stimulates ferroptosis possibly in a GSH-dependent manner. |
| PLIN4 | Perilipin 4 | HGNC:29393 | Upregulated in cells treated with ferroptosis inducer erastin. Stimulates ferroptosis possibly in a GSH-dependent manner. |
| HIC1 | HIC ZBTB transcriptional repressor 1 | HGNC:4909 | Essential for stimulation of pro-ferroptotic gene transcription upon ferroptosis induction. |
| STMN1 | Stathmin 1 | HGNC:6510 | Downregulated in cells treated with ferroptosis inducer erastin. Suppresses ferroptosis possibly in a GSH-dependent manner. |
| RRM2 | Ribonucleotide reductase regulatory subunit M2 | HGNC:10452 | Downregulated in cells treated with ferroptosis inducer erastin. Suppresses ferroptosis possibly in a GSH-dependent manner. |
| CAPG | Capping actin protein, gelsolin like | HGNC:1474 | Downregulated in cells treated with ferroptosis inducer erastin. Suppresses ferroptosis possibly in a GSH-dependent manner. |
| HNF4A | Hepatocyte nuclear factor 4 alpha | HGNC:5024 | Essential for stimulation of anti-ferroptotic gene transcription. |
| NGB | Neuroglobin | HGNC:14077 | Human neuroglobin (hNgb)-EGFP-expressing SH-SY5Y cells to be significantly more resistant to ferroptosis induction. |
| YWHAE | Tyrosine 3-monooxygenase/tryptophan 5-monooxygenase activation protein epsilon | HGNC:12851 | Required by RSL3 (a ferroptosis inducer) to inactivate GPX4 (a ferroptosis inhibitor). |
| GABPB1 | GA binding protein transcription factor subunit beta 1 | HGNC:4074 | Downregulated by lncRNA GABPB1-AS1 upon erastin treatment. |
| AURKA | Aurora kinase A | HGNC:11393 | Inhibition of AURKA or reconstitution of miR-4715-3p inhibited GPX4 and induced cell death, suggesting a link between AURKA and ferroptosis. |
| MIR4715 | microRNA 4715 | HGNC:41666 | Inhibition of AURKA or reconstitution of miR-4715-3p inhibited GPX4 and induced cell death, suggesting a link between AURKA and ferroptosis. |
| RIPK1 | Receptor interacting serine/threonine kinase 1 | HGNC:10019 | Reduced expression results in resistance to ferroptosis. |
| PRDX1 | Peroxiredoxin 1 | HGNC:9352 | Necessary to ferroptosis-related lipid peroxidation. |
| MIR30B | microRNA 30b | HGNC:31625 | Upregulation of miR-30b-5p in preeclampsia models plays a pivotal role in ferroptosis. |

# Appendix 2

## 56 DEGs linked to ferroptosis

**Table 2. 56 DEGs linked to ferroptosis**.

| gene | conMean | treatMean | logFC | pValue |
| --- | --- | --- | --- | --- |
| ACSF2 | 35.16399163 | 6.462490528 | -2.443936681 | 2.12E-11 |
| CYBB | 4.305802781 | 9.487467947 | 1.139740865 | 9.72E-06 |
| NOX3 | 0.004767266 | 0.001883607 | -1.339663883 | 1.68E-08 |
| NOX4 | 17.91882553 | 6.343711328 | -1.498077053 | 0.0003678 |
| G6PD | 10.02010028 | 29.39037246 | 1.552446695 | 3.22E-14 |
| TFR2 | 0.037508656 | 0.252733051 | 2.752318882 | 4.63E-12 |
| ALOX5 | 3.297104906 | 20.22767473 | 2.617058789 | 3.43E-15 |
| HMOX1 | 28.314257 | 89.81349263 | 1.665403524 | 0.000312963 |
| ALOX15B | 0.067268553 | 3.502896639 | 5.70247238 | 1.19E-15 |
| ALOXE3 | 0.026752103 | 0.087775835 | 1.714171502 | 0.002986142 |
| ACO1 | 28.69987347 | 12.31479867 | -1.220651335 | 8.59E-10 |
| WIPI1 | 8.213122469 | 17.69784393 | 1.107570896 | 8.71E-13 |
| CDKN2A | 0.056548925 | 1.306742867 | 4.530331873 | 4.06E-18 |
| MYB | 0.031253107 | 0.116455912 | 1.897712349 | 3.21E-10 |
| CHAC1 | 5.431018175 | 0.998558122 | -2.443304382 | 8.93E-16 |
| LINC00472 | 4.69328925 | 1.302543148 | -1.849268213 | 4.86E-16 |
| PRKAA2 | 8.686692063 | 3.995489382 | -1.120434675 | 7.44E-17 |
| EPAS1 | 71.17760156 | 19.44846308 | -1.871767169 | 7.51E-18 |
| HILPDA | 3.696774966 | 12.53747298 | 1.761907461 | 2.52E-07 |
| ATF3 | 42.76978938 | 14.96984336 | -1.514532977 | 5.78E-11 |
| MIOX | 358.8263333 | 91.64314854 | -1.969186839 | 1.18E-06 |
| PTGS2 | 2.529006653 | 0.508222151 | -2.315039671 | 1.78E-14 |
| DUSP1 | 220.2211306 | 99.25798709 | -1.149697802 | 1.36E-10 |
| NOS2 | 0.622319069 | 0.242911103 | -1.35722602 | 8.36E-12 |
| NCF2 | 1.606364247 | 5.906505587 | 1.87850579 | 2.54E-10 |
| ALB | 72.17686352 | 0.760357493 | -6.56871476 | 1.64E-16 |
| TXNRD1 | 11.72726997 | 34.38000574 | 1.551702583 | 0.0003678 |
| SLC7A11 | 0.049419119 | 0.899788112 | 4.186444122 | 2.93E-08 |
| ASNS | 2.723180563 | 8.129323484 | 1.577842653 | 7.46E-16 |
| JDP2 | 3.773935781 | 1.844202985 | -1.033072417 | 9.36E-15 |
| SLC1A4 | 1.201782666 | 2.501959843 | 1.057882616 | 1.88E-12 |
| PCK2 | 79.56581491 | 13.84305064 | -2.522986806 | 4.92E-07 |
| GPT2 | 9.5360575 | 4.466968723 | -1.094096782 | 4.61E-09 |
| PSAT1 | 51.00619425 | 15.29694123 | -1.737429259 | 3.20E-09 |
| TRIB3 | 1.843433441 | 7.543768591 | 2.032890094 | 3.40E-07 |
| HAMP | 0.052427504 | 1.730404986 | 5.044642064 | 6.23E-19 |
| HSPB1 | 246.4513563 | 693.805819 | 1.49322902 | 3.65E-14 |
| DRD4 | 0.361113284 | 1.168152005 | 1.693704617 | 4.62E-07 |
| SLC2A12 | 3.045033441 | 0.225806482 | -3.753299267 | 1.04E-19 |
| SLC2A14 | 0.027761324 | 0.142654695 | 2.361378966 | 1.12E-06 |
| NNMT | 14.92983003 | 130.0039071 | 3.122285335 | 9.36E-11 |
| RRM2 | 0.291312819 | 2.00959965 | 2.786267027 | 2.70E-15 |
| CAPG | 62.80647875 | 147.1676348 | 1.228475136 | 3.29E-10 |
| AURKA | 0.964694984 | 2.482437567 | 1.363612664 | 3.54E-15 |
| AKR1C1 | 6.627962928 | 35.0763257 | 2.403860193 | 4.21E-10 |
| AKR1C2 | 1.285675444 | 13.20046466 | 3.359990314 | 2.60E-11 |
| AKR1C3 | 17.29169688 | 40.06542492 | 1.212278327 | 0.001004863 |
| MUC1 | 75.70675594 | 35.42383427 | -1.095701672 | 1.89E-09 |
| MT1G | 732.6799774 | 82.02987844 | -3.158961795 | 3.68E-19 |
| SCD | 7.835669781 | 26.24465093 | 1.743894904 | 8.60E-09 |
| CDKN1A | 46.60617172 | 115.9357632 | 1.314732751 | 8.48E-12 |
| CD44 | 5.724324313 | 15.56384444 | 1.44302115 | 0.000114664 |
| CA9 | 0.183741693 | 10.44999034 | 5.829678773 | 5.88E-12 |
| ZFP36 | 177.7933997 | 88.05983713 | -1.013645686 | 2.42E-06 |
| PROM2 | 36.1590025 | 5.51514651 | -2.712883763 | 8.79E-18 |
| GCH1 | 8.268610219 | 2.438450645 | -1.761680091 | 7.70E-16 |

# Appendix 3

## GO and KEGG enrichment analysis

**Table 3a. GO enrichment analysis**.

| ONTOLOGY | ID | Description | GeneRatio | pvalue | qvalue |
| --- | --- | --- | --- | --- | --- |
| BP | GO:0006979 | response to oxidative stress | 16/55 | 8.72E-14 | 7.48E-11 |
| BP | GO:0034599 | cellular response to oxidative stress | 14/55 | 1.13E-13 | 7.48E-11 |
| BP | GO:0062197 | cellular response to chemical stress | 14/55 | 8.63E-13 | 3.79E-10 |
| BP | GO:0042594 | response to starvation | 10/55 | 2.27E-10 | 7.49E-08 |
| BP | GO:0031667 | response to nutrient levels | 13/55 | 4.09E-10 | 9.70E-08 |
| BP | GO:0072593 | reactive oxygen species metabolic process | 11/55 | 4.42E-10 | 9.70E-08 |
| BP | GO:0009991 | response to extracellular stimulus | 13/55 | 8.11E-10 | 1.53E-07 |
| BP | GO:0006631 | fatty acid metabolic process | 12/55 | 1.08E-09 | 1.77E-07 |
| BP | GO:0033559 | unsaturated fatty acid metabolic process | 8/55 | 1.43E-09 | 2.10E-07 |
| BP | GO:0046394 | carboxylic acid biosynthetic process | 11/55 | 2.18E-09 | 2.87E-07 |
| BP | GO:0016053 | organic acid biosynthetic process | 11/55 | 2.81E-09 | 3.36E-07 |
| BP | GO:0010039 | response to iron ion | 5/55 | 1.63E-08 | 1.79E-06 |
| BP | GO:0006633 | fatty acid biosynthetic process | 8/55 | 2.86E-08 | 2.90E-06 |
| BP | GO:1901617 | organic hydroxy compound biosynthetic process | 9/55 | 4.23E-08 | 3.98E-06 |
| BP | GO:0120254 | olefinic compound metabolic process | 7/55 | 5.26E-08 | 4.34E-06 |
| BP | GO:0006801 | superoxide metabolic process | 6/55 | 5.27E-08 | 4.34E-06 |
| BP | GO:0010038 | response to metal ion | 10/55 | 6.26E-08 | 4.84E-06 |
| BP | GO:0006690 | icosanoid metabolic process | 7/55 | 6.61E-08 | 4.84E-06 |
| BP | GO:1901654 | response to ketone | 8/55 | 8.39E-08 | 5.82E-06 |
| BP | GO:0019372 | lipoxygenase pathway | 4/55 | 1.15E-07 | 7.56E-06 |
| BP | GO:0070482 | response to oxygen levels | 10/55 | 1.44E-07 | 9.05E-06 |
| BP | GO:0031669 | cellular response to nutrient levels | 8/55 | 1.61E-07 | 9.62E-06 |
| BP | GO:0072330 | monocarboxylic acid biosynthetic process | 8/55 | 2.63E-07 | 1.51E-05 |
| BP | GO:0009267 | cellular response to starvation | 7/55 | 3.51E-07 | 1.87E-05 |
| BP | GO:0097305 | response to alcohol | 8/55 | 3.55E-07 | 1.87E-05 |
| BP | GO:0031668 | cellular response to extracellular stimulus | 8/55 | 3.79E-07 | 1.92E-05 |
| BP | GO:0009636 | response to toxic substance | 8/55 | 4.31E-07 | 2.10E-05 |
| BP | GO:0001666 | response to hypoxia | 9/55 | 6.73E-07 | 3.17E-05 |
| BP | GO:0019369 | arachidonic acid metabolic process | 5/55 | 7.77E-07 | 3.53E-05 |
| BP | GO:0036293 | response to decreased oxygen levels | 9/55 | 8.92E-07 | 3.92E-05 |
| BP | GO:0006066 | alcohol metabolic process | 9/55 | 1.20E-06 | 5.09E-05 |
| BP | GO:2000377 | regulation of reactive oxygen species metabolic process | 7/55 | 1.36E-06 | 5.61E-05 |
| BP | GO:0031100 | animal organ regeneration | 5/55 | 2.26E-06 | 9.03E-05 |
| BP | GO:0042759 | long-chain fatty acid biosynthetic process | 4/55 | 2.49E-06 | 9.62E-05 |
| BP | GO:0071496 | cellular response to external stimulus | 8/55 | 2.56E-06 | 9.62E-05 |
| BP | GO:0030647 | aminoglycoside antibiotic metabolic process | 3/55 | 2.77E-06 | 0.00010158 |
| BP | GO:0055076 | transition metal ion homeostasis | 6/55 | 3.10E-06 | 0.000109845 |
| BP | GO:0042554 | superoxide anion generation | 4/55 | 3.17E-06 | 0.000109845 |
| BP | GO:0002262 | myeloid cell homeostasis | 6/55 | 3.96E-06 | 0.000133654 |
| BP | GO:0071276 | cellular response to cadmium ion | 4/55 | 4.44E-06 | 0.000146161 |
| BP | GO:0055072 | iron ion homeostasis | 5/55 | 5.09E-06 | 0.000162928 |
| BP | GO:0031960 | response to corticosteroid | 6/55 | 5.19E-06 | 0.000162928 |
| BP | GO:0045454 | cell redox homeostasis | 4/55 | 7.34E-06 | 0.000224871 |
| BP | GO:0042180 | cellular ketone metabolic process | 7/55 | 8.65E-06 | 0.000258967 |
| BP | GO:0042448 | progesterone metabolic process | 3/55 | 1.04E-05 | 0.000304954 |
| BP | GO:0006692 | prostanoid metabolic process | 4/55 | 1.14E-05 | 0.000320891 |
| BP | GO:0006693 | prostaglandin metabolic process | 4/55 | 1.14E-05 | 0.000320891 |
| BP | GO:0006636 | unsaturated fatty acid biosynthetic process | 4/55 | 1.46E-05 | 0.000400928 |
| BP | GO:0043618 | regulation of transcription from RNA polymerase II promoter in response to stress | 5/55 | 1.62E-05 | 0.000436835 |
| BP | GO:0097193 | intrinsic apoptotic signaling pathway | 7/55 | 1.74E-05 | 0.00045918 |
| BP | GO:0071248 | cellular response to metal ion | 6/55 | 1.80E-05 | 0.000465739 |
| BP | GO:1901605 | alpha-amino acid metabolic process | 6/55 | 1.91E-05 | 0.000484809 |
| BP | GO:0043620 | regulation of DNA-templated transcription in response to stress | 5/55 | 2.11E-05 | 0.000518161 |
| BP | GO:0019748 | secondary metabolic process | 4/55 | 2.12E-05 | 0.000518161 |
| BP | GO:0046916 | cellular transition metal ion homeostasis | 5/55 | 2.38E-05 | 0.000571377 |
| BP | GO:0071379 | cellular response to prostaglandin stimulus | 3/55 | 2.58E-05 | 0.000587579 |
| BP | GO:1902644 | tertiary alcohol metabolic process | 3/55 | 2.58E-05 | 0.000587579 |
| BP | GO:0001676 | long-chain fatty acid metabolic process | 5/55 | 2.59E-05 | 0.000587579 |
| BP | GO:1903409 | reactive oxygen species biosynthetic process | 5/55 | 2.91E-05 | 0.000619043 |
| BP | GO:0071456 | cellular response to hypoxia | 6/55 | 2.93E-05 | 0.000619043 |
| BP | GO:0046686 | response to cadmium ion | 4/55 | 2.98E-05 | 0.000619043 |
| BP | GO:0070542 | response to fatty acid | 4/55 | 2.98E-05 | 0.000619043 |
| BP | GO:0016137 | glycoside metabolic process | 3/55 | 3.01E-05 | 0.000619043 |
| BP | GO:0043651 | linoleic acid metabolic process | 3/55 | 3.01E-05 | 0.000619043 |
| BP | GO:0036294 | cellular response to decreased oxygen levels | 6/55 | 3.63E-05 | 0.000735216 |
| BP | GO:0071241 | cellular response to inorganic substance | 6/55 | 3.82E-05 | 0.000762738 |
| BP | GO:0045444 | fat cell differentiation | 6/55 | 4.34E-05 | 0.00085375 |
| BP | GO:0045598 | regulation of fat cell differentiation | 5/55 | 4.71E-05 | 0.000913695 |
| BP | GO:0001889 | liver development | 5/55 | 5.05E-05 | 0.000951634 |
| BP | GO:0098754 | detoxification | 5/55 | 5.05E-05 | 0.000951634 |
| BP | GO:0006879 | cellular iron ion homeostasis | 4/55 | 5.14E-05 | 0.000954507 |
| BP | GO:0061008 | hepaticobiliary system development | 5/55 | 5.41E-05 | 0.000990886 |
| BP | GO:0071453 | cellular response to oxygen levels | 6/55 | 5.55E-05 | 0.001002353 |
| BP | GO:0007050 | cell cycle arrest | 6/55 | 6.11E-05 | 0.001087587 |
| BP | GO:0042493 | response to drug | 7/55 | 7.89E-05 | 0.001387271 |
| BP | GO:0048872 | homeostasis of number of cells | 6/55 | 8.41E-05 | 0.001457828 |
| BP | GO:0034694 | response to prostaglandin | 3/55 | 9.01E-05 | 0.001508809 |
| BP | GO:0046685 | response to arsenic-containing substance | 3/55 | 9.01E-05 | 0.001508809 |
| BP | GO:0046165 | alcohol biosynthetic process | 5/55 | 9.04E-05 | 0.001508809 |
| BP | GO:0097421 | liver regeneration | 3/55 | 9.95E-05 | 0.001639526 |
| BP | GO:1900407 | regulation of cellular response to oxidative stress | 4/55 | 0.000104993 | 0.001693216 |
| BP | GO:0015711 | organic anion transport | 7/55 | 0.000105353 | 0.001693216 |
| BP | GO:0097306 | cellular response to alcohol | 4/55 | 0.000131434 | 0.002065862 |
| BP | GO:1901661 | quinone metabolic process | 3/55 | 0.000131674 | 0.002065862 |
| BP | GO:0072525 | pyridine-containing compound biosynthetic process | 3/55 | 0.000143721 | 0.00222835 |
| BP | GO:1901655 | cellular response to ketone | 4/55 | 0.000149441 | 0.002290091 |
| BP | GO:1902882 | regulation of response to oxidative stress | 4/55 | 0.000155821 | 0.002360414 |
| BP | GO:0015718 | monocarboxylic acid transport | 5/55 | 0.000167996 | 0.00251592 |
| BP | GO:0071398 | cellular response to fatty acid | 3/55 | 0.000184103 | 0.002726157 |
| BP | GO:0050892 | intestinal absorption | 3/55 | 0.000199034 | 0.002879981 |
| BP | GO:0006986 | response to unfolded protein | 5/55 | 0.000201047 | 0.002879981 |
| BP | GO:0010565 | regulation of cellular ketone metabolic process | 5/55 | 0.000201047 | 0.002879981 |
| BP | GO:0008630 | intrinsic apoptotic signaling pathway in response to DNA damage | 4/55 | 0.000206134 | 0.002921111 |
| BP | GO:2000379 | positive regulation of reactive oxygen species metabolic process | 4/55 | 0.000214173 | 0.002931191 |
| BP | GO:0008207 | C21-steroid hormone metabolic process | 3/55 | 0.000214728 | 0.002931191 |
| BP | GO:0034976 | response to endoplasmic reticulum stress | 6/55 | 0.000215742 | 0.002931191 |
| BP | GO:0051348 | negative regulation of transferase activity | 6/55 | 0.000215742 | 0.002931191 |
| BP | GO:0098869 | cellular oxidant detoxification | 4/55 | 0.000222434 | 0.002991275 |
| BP | GO:0072524 | pyridine-containing compound metabolic process | 3/55 | 0.000231203 | 0.003077787 |
| BP | GO:0031099 | regeneration | 5/55 | 0.000238845 | 0.003147722 |
| BP | GO:0018958 | phenol-containing compound metabolic process | 4/55 | 0.00025779 | 0.003330789 |
| BP | GO:0030330 | DNA damage response, signal transduction by p53 class mediator | 4/55 | 0.00025779 | 0.003330789 |
| BP | GO:0062012 | regulation of small molecule metabolic process | 7/55 | 0.000265377 | 0.003395529 |
| BP | GO:0042771 | intrinsic apoptotic signaling pathway in response to DNA damage by p53 class mediator | 3/55 | 0.000285475 | 0.003617558 |
| BP | GO:0046890 | regulation of lipid biosynthetic process | 5/55 | 0.000308895 | 0.003877058 |
| BP | GO:0006953 | acute-phase response | 3/55 | 0.000325859 | 0.004051397 |
| BP | GO:0035966 | response to topologically incorrect protein | 5/55 | 0.000330456 | 0.004070151 |
| BP | GO:1990748 | cellular detoxification | 4/55 | 0.000351867 | 0.004293739 |
| BP | GO:0006869 | lipid transport | 7/55 | 0.000366589 | 0.004429977 |
| BP | GO:0042398 | cellular modified amino acid biosynthetic process | 3/55 | 0.000369754 | 0.004429977 |
| BP | GO:0008202 | steroid metabolic process | 6/55 | 0.000379909 | 0.004510629 |
| BP | GO:0048545 | response to steroid hormone | 6/55 | 0.000386084 | 0.004543014 |
| BP | GO:0006520 | cellular amino acid metabolic process | 6/55 | 0.000392337 | 0.00457574 |
| BP | GO:2000134 | negative regulation of G1/S transition of mitotic cell cycle | 4/55 | 0.000400692 | 0.004632193 |
| BP | GO:0034101 | erythrocyte homeostasis | 4/55 | 0.000426838 | 0.004891543 |
| BP | GO:0097237 | cellular response to toxic substance | 4/55 | 0.000440358 | 0.005000076 |
| BP | GO:0019371 | cyclooxygenase pathway | 2/55 | 0.000451485 | 0.005000076 |
| BP | GO:0035404 | histone-serine phosphorylation | 2/55 | 0.000451485 | 0.005000076 |
| BP | GO:0043619 | regulation of transcription from RNA polymerase II promoter in response to oxidative stress | 2/55 | 0.000451485 | 0.005000076 |
| BP | GO:1902807 | negative regulation of cell cycle G1/S phase transition | 4/55 | 0.000468311 | 0.005061755 |
| BP | GO:0006584 | catecholamine metabolic process | 3/55 | 0.000468576 | 0.005061755 |
| BP | GO:0009712 | catechol-containing compound metabolic process | 3/55 | 0.000468576 | 0.005061755 |
| BP | GO:0000302 | response to reactive oxygen species | 5/55 | 0.000484756 | 0.005193965 |
| BP | GO:0045471 | response to ethanol | 4/55 | 0.000497507 | 0.005287599 |
| BP | GO:0042304 | regulation of fatty acid biosynthetic process | 3/55 | 0.000523746 | 0.005521932 |
| BP | GO:0042770 | signal transduction in response to DNA damage | 4/55 | 0.000576146 | 0.006001691 |
| BP | GO:0006977 | DNA damage response, signal transduction by p53 class mediator resulting in cell cycle arrest | 3/55 | 0.000582912 | 0.006001691 |
| BP | GO:0046456 | icosanoid biosynthetic process | 3/55 | 0.000582912 | 0.006001691 |
| BP | GO:0010948 | negative regulation of cell cycle process | 6/55 | 0.000602197 | 0.006152189 |
| BP | GO:0072431 | signal transduction involved in mitotic G1 DNA damage checkpoint | 3/55 | 0.00061403 | 0.006177304 |
| BP | GO:1902400 | intracellular signal transduction involved in G1 DNA damage checkpoint | 3/55 | 0.00061403 | 0.006177304 |
| BP | GO:0051384 | response to glucocorticoid | 4/55 | 0.000645125 | 0.006440961 |
| BP | GO:1902402 | signal transduction involved in mitotic DNA damage checkpoint | 3/55 | 0.000679408 | 0.006681998 |
| BP | GO:1902403 | signal transduction involved in mitotic DNA integrity checkpoint | 3/55 | 0.000679408 | 0.006681998 |
| BP | GO:0007586 | digestion | 4/55 | 0.000700566 | 0.006792551 |
| BP | GO:0006469 | negative regulation of protein kinase activity | 5/55 | 0.000700957 | 0.006792551 |
| BP | GO:0071384 | cellular response to corticosteroid stimulus | 3/55 | 0.000713695 | 0.006865511 |
| BP | GO:0045926 | negative regulation of growth | 5/55 | 0.000727322 | 0.0069459 |
| BP | GO:0034356 | NAD biosynthesis via nicotinamide riboside salvage pathway | 2/55 | 0.000742819 | 0.007042861 |
| BP | GO:0072413 | signal transduction involved in mitotic cell cycle checkpoint | 3/55 | 0.000749067 | 0.007051366 |
| BP | GO:0031571 | mitotic G1 DNA damage checkpoint | 3/55 | 0.000785536 | 0.007264804 |
| BP | GO:1901570 | fatty acid derivative biosynthetic process | 3/55 | 0.000785536 | 0.007264804 |
| BP | GO:0006790 | sulfur compound metabolic process | 6/55 | 0.000788278 | 0.007264804 |
| BP | GO:1901991 | negative regulation of mitotic cell cycle phase transition | 5/55 | 0.000796487 | 0.007289482 |
| BP | GO:0044783 | G1 DNA damage checkpoint | 3/55 | 0.000823117 | 0.007430015 |
| BP | GO:0044819 | mitotic G1/S transition checkpoint | 3/55 | 0.000823117 | 0.007430015 |
| BP | GO:0071901 | negative regulation of protein serine/threonine kinase activity | 4/55 | 0.000842932 | 0.007516368 |
| BP | GO:0031331 | positive regulation of cellular catabolic process | 6/55 | 0.000855439 | 0.007516368 |
| BP | GO:0042574 | retinal metabolic process | 2/55 | 0.000855497 | 0.007516368 |
| BP | GO:1902166 | negative regulation of intrinsic apoptotic signaling pathway in response to DNA damage by p53 class mediator | 2/55 | 0.000855497 | 0.007516368 |
| BP | GO:0006749 | glutathione metabolic process | 3/55 | 0.000861823 | 0.007521804 |
| BP | GO:0071479 | cellular response to ionizing radiation | 3/55 | 0.000901667 | 0.007817779 |
| BP | GO:0046883 | regulation of hormone secretion | 5/55 | 0.000949444 | 0.008178219 |
| BP | GO:0090594 | inflammatory response to wounding | 2/55 | 0.000975884 | 0.00835138 |
| BP | GO:0072331 | signal transduction by p53 class mediator | 5/55 | 0.000999331 | 0.008496855 |
| BP | GO:0009165 | nucleotide biosynthetic process | 5/55 | 0.001016385 | 0.008586468 |
| BP | GO:0033673 | negative regulation of kinase activity | 5/55 | 0.001068851 | 0.008817399 |
| BP | GO:1901293 | nucleoside phosphate biosynthetic process | 5/55 | 0.001068851 | 0.008817399 |
| BP | GO:0019216 | regulation of lipid metabolic process | 6/55 | 0.001083756 | 0.008817399 |
| BP | GO:1901988 | negative regulation of cell cycle phase transition | 5/55 | 0.00108678 | 0.008817399 |
| BP | GO:0006750 | glutathione biosynthetic process | 2/55 | 0.001103936 | 0.008817399 |
| BP | GO:0006978 | DNA damage response, signal transduction by p53 class mediator resulting in transcription of p21 class mediator | 2/55 | 0.001103936 | 0.008817399 |
| BP | GO:0071850 | mitotic cell cycle arrest | 2/55 | 0.001103936 | 0.008817399 |
| BP | GO:0090399 | replicative senescence | 2/55 | 0.001103936 | 0.008817399 |
| BP | GO:1902165 | regulation of intrinsic apoptotic signaling pathway in response to DNA damage by p53 class mediator | 2/55 | 0.001103936 | 0.008817399 |
| BP | GO:0044106 | cellular amine metabolic process | 4/55 | 0.001159614 | 0.009206318 |
| BP | GO:0007093 | mitotic cell cycle checkpoint | 4/55 | 0.001186986 | 0.009367199 |
| BP | GO:0006695 | cholesterol biosynthetic process | 3/55 | 0.001213524 | 0.009447065 |
| BP | GO:1902653 | secondary alcohol biosynthetic process | 3/55 | 0.001213524 | 0.009447065 |
| BP | GO:0000082 | G1/S transition of mitotic cell cycle | 5/55 | 0.001218611 | 0.009447065 |
| BP | GO:0042772 | DNA damage response, signal transduction resulting in transcription | 2/55 | 0.001239608 | 0.00945689 |
| BP | GO:0071243 | cellular response to arsenic-containing substance | 2/55 | 0.001239608 | 0.00945689 |
| BP | GO:0048732 | gland development | 6/55 | 0.001244754 | 0.00945689 |
| BP | GO:0006809 | nitric oxide biosynthetic process | 3/55 | 0.001262933 | 0.00945689 |
| BP | GO:0072401 | signal transduction involved in DNA integrity checkpoint | 3/55 | 0.001262933 | 0.00945689 |
| BP | GO:0072422 | signal transduction involved in DNA damage checkpoint | 3/55 | 0.001262933 | 0.00945689 |
| BP | GO:0009308 | amine metabolic process | 4/55 | 0.001301017 | 0.009687025 |
| BP | GO:0007584 | response to nutrient | 4/55 | 0.001360815 | 0.009942578 |
| BP | GO:0009064 | glutamine family amino acid metabolic process | 3/55 | 0.001365516 | 0.009942578 |
| BP | GO:0043536 | positive regulation of blood vessel endothelial cell migration | 3/55 | 0.001365516 | 0.009942578 |
| BP | GO:0072332 | intrinsic apoptotic signaling pathway by p53 class mediator | 3/55 | 0.001365516 | 0.009942578 |
| BP | GO:0019184 | nonribosomal peptide biosynthetic process | 2/55 | 0.001382856 | 0.010013512 |
| BP | GO:0048469 | cell maturation | 4/55 | 0.001391423 | 0.010020485 |
| BP | GO:0046942 | carboxylic acid transport | 5/55 | 0.001405105 | 0.010064023 |
| BP | GO:0072395 | signal transduction involved in cell cycle checkpoint | 3/55 | 0.001418714 | 0.010106574 |
| BP | GO:0008652 | cellular amino acid biosynthetic process | 3/55 | 0.001473199 | 0.010382467 |
| BP | GO:0046209 | nitric oxide metabolic process | 3/55 | 0.001473199 | 0.010382467 |
| BP | GO:0016126 | sterol biosynthetic process | 3/55 | 0.001528982 | 0.010637756 |
| BP | GO:2001057 | reactive nitrogen species metabolic process | 3/55 | 0.001528982 | 0.010637756 |
| BP | GO:0006925 | inflammatory cell apoptotic process | 2/55 | 0.001533638 | 0.010637756 |
| BP | GO:0001659 | temperature homeostasis | 4/55 | 0.001551719 | 0.010706815 |
| BP | GO:2000045 | regulation of G1/S transition of mitotic cell cycle | 4/55 | 0.001653843 | 0.011331487 |
| BP | GO:0001819 | positive regulation of cytokine production | 6/55 | 0.001659447 | 0.011331487 |
| BP | GO:0035357 | peroxisome proliferator activated receptor signaling pathway | 2/55 | 0.001691909 | 0.011376318 |
| BP | GO:0071636 | positive regulation of transforming growth factor beta production | 2/55 | 0.001691909 | 0.011376318 |
| BP | GO:1902254 | negative regulation of intrinsic apoptotic signaling pathway by p53 class mediator | 2/55 | 0.001691909 | 0.011376318 |
| BP | GO:0044843 | cell cycle G1/S phase transition | 5/55 | 0.001736064 | 0.011574656 |
| BP | GO:0008217 | regulation of blood pressure | 4/55 | 0.001760558 | 0.011574656 |
| BP | GO:1901796 | regulation of signal transduction by p53 class mediator | 4/55 | 0.001760558 | 0.011574656 |
| BP | GO:0071158 | positive regulation of cell cycle arrest | 3/55 | 0.00176532 | 0.011574656 |
| BP | GO:1901568 | fatty acid derivative metabolic process | 3/55 | 0.00176532 | 0.011574656 |
| BP | GO:0046879 | hormone secretion | 5/55 | 0.001840342 | 0.012006818 |
| BP | GO:0009314 | response to radiation | 6/55 | 0.00186037 | 0.012077692 |
| BP | GO:0009896 | positive regulation of catabolic process | 6/55 | 0.001924112 | 0.012430279 |
| BP | GO:0002791 | regulation of peptide secretion | 5/55 | 0.002005293 | 0.012805313 |
| BP | GO:0031058 | positive regulation of histone modification | 3/55 | 0.002023317 | 0.012805313 |
| BP | GO:0033032 | regulation of myeloid cell apoptotic process | 2/55 | 0.002030747 | 0.012805313 |
| BP | GO:0036499 | PERK-mediated unfolded protein response | 2/55 | 0.002030747 | 0.012805313 |
| BP | GO:0061050 | regulation of cell growth involved in cardiac muscle cell development | 2/55 | 0.002030747 | 0.012805313 |
| BP | GO:0009914 | hormone transport | 5/55 | 0.00212112 | 0.01331149 |
| BP | GO:0036003 | positive regulation of transcription from RNA polymerase II promoter in response to stress | 2/55 | 0.002211227 | 0.013811204 |
| BP | GO:0006694 | steroid biosynthetic process | 4/55 | 0.002278646 | 0.014165167 |
| BP | GO:0045930 | negative regulation of mitotic cell cycle | 5/55 | 0.002399555 | 0.014846762 |
| BP | GO:0015849 | organic acid transport | 5/55 | 0.002498028 | 0.015344115 |
| BP | GO:1902806 | regulation of cell cycle G1/S phase transition | 4/55 | 0.002503223 | 0.015344115 |
| BP | GO:0032496 | response to lipopolysaccharide | 5/55 | 0.002565292 | 0.015651783 |
| BP | GO:0034308 | primary alcohol metabolic process | 3/55 | 0.002606967 | 0.015760128 |
| BP | GO:1904035 | regulation of epithelial cell apoptotic process | 3/55 | 0.002606967 | 0.015760128 |
| BP | GO:0006575 | cellular modified amino acid metabolic process | 4/55 | 0.002645045 | 0.015917311 |
| BP | GO:2001243 | negative regulation of intrinsic apoptotic signaling pathway | 3/55 | 0.00268646 | 0.016093054 |
| BP | GO:0043648 | dicarboxylic acid metabolic process | 3/55 | 0.002767438 | 0.016428794 |
| BP | GO:0048010 | vascular endothelial growth factor receptor signaling pathway | 3/55 | 0.002767438 | 0.016428794 |
| BP | GO:0015949 | nucleobase-containing small molecule interconversion | 2/55 | 0.002796399 | 0.016452499 |
| BP | GO:0140467 | integrated stress response signaling | 2/55 | 0.002796399 | 0.016452499 |
| BP | GO:0046364 | monosaccharide biosynthetic process | 3/55 | 0.00284991 | 0.016692805 |
| BP | GO:0019217 | regulation of fatty acid metabolic process | 3/55 | 0.002933884 | 0.017108632 |
| BP | GO:0071900 | regulation of protein serine/threonine kinase activity | 6/55 | 0.003002261 | 0.017223696 |
| BP | GO:0033028 | myeloid cell apoptotic process | 2/55 | 0.003005893 | 0.017223696 |
| BP | GO:0071480 | cellular response to gamma radiation | 2/55 | 0.003005893 | 0.017223696 |
| BP | GO:1902253 | regulation of intrinsic apoptotic signaling pathway by p53 class mediator | 2/55 | 0.003005893 | 0.017223696 |
| BP | GO:0022600 | digestive system process | 3/55 | 0.003019371 | 0.017226031 |
| BP | GO:1905269 | positive regulation of chromatin organization | 3/55 | 0.003106378 | 0.017646033 |
| BP | GO:0000075 | cell cycle checkpoint | 4/55 | 0.00321232 | 0.017995594 |
| BP | GO:0090276 | regulation of peptide hormone secretion | 4/55 | 0.00321232 | 0.017995594 |
| BP | GO:0009435 | NAD biosynthetic process | 2/55 | 0.003222534 | 0.017995594 |
| BP | GO:0072337 | modified amino acid transport | 2/55 | 0.003222534 | 0.017995594 |
| BP | GO:0044773 | mitotic DNA damage checkpoint | 3/55 | 0.00328499 | 0.018266969 |
| BP | GO:0002237 | response to molecule of bacterial origin | 5/55 | 0.003312453 | 0.018342289 |
| BP | GO:0006576 | cellular biogenic amine metabolic process | 3/55 | 0.003376612 | 0.018619329 |
| BP | GO:2001233 | regulation of apoptotic signaling pathway | 5/55 | 0.003394949 | 0.01864244 |
| BP | GO:1902230 | negative regulation of intrinsic apoptotic signaling pathway in response to DNA damage | 2/55 | 0.003446281 | 0.018845788 |
| BP | GO:0044774 | mitotic DNA integrity checkpoint | 3/55 | 0.003660843 | 0.019779671 |
| BP | GO:0001516 | prostaglandin biosynthetic process | 2/55 | 0.003677091 | 0.019779671 |
| BP | GO:0046457 | prostanoid biosynthetic process | 2/55 | 0.003677091 | 0.019779671 |
| BP | GO:1901797 | negative regulation of signal transduction by p53 class mediator | 2/55 | 0.003677091 | 0.019779671 |
| BP | GO:0071695 | anatomical structure maturation | 4/55 | 0.003734284 | 0.020005663 |
| BP | GO:0002526 | acute inflammatory response | 3/55 | 0.003758736 | 0.020055137 |
| BP | GO:0071156 | regulation of cell cycle arrest | 3/55 | 0.003858217 | 0.020393116 |
| BP | GO:0010165 | response to X-ray | 2/55 | 0.003914925 | 0.020393116 |
| BP | GO:0019359 | nicotinamide nucleotide biosynthetic process | 2/55 | 0.003914925 | 0.020393116 |
| BP | GO:0019363 | pyridine nucleotide biosynthetic process | 2/55 | 0.003914925 | 0.020393116 |
| BP | GO:0045736 | negative regulation of cyclin-dependent protein serine/threonine kinase activity | 2/55 | 0.003914925 | 0.020393116 |
| BP | GO:0051385 | response to mineralocorticoid | 2/55 | 0.003914925 | 0.020393116 |
| BP | GO:1904659 | glucose transmembrane transport | 3/55 | 0.003959294 | 0.020543042 |
| BP | GO:0001933 | negative regulation of protein phosphorylation | 5/55 | 0.004014536 | 0.020747985 |
| BP | GO:2001234 | negative regulation of apoptotic signaling pathway | 4/55 | 0.004112612 | 0.021084992 |
| BP | GO:0003298 | physiological muscle hypertrophy | 2/55 | 0.004159739 | 0.021084992 |
| BP | GO:0003301 | physiological cardiac muscle hypertrophy | 2/55 | 0.004159739 | 0.021084992 |
| BP | GO:0061049 | cell growth involved in cardiac muscle cell development | 2/55 | 0.004159739 | 0.021084992 |
| BP | GO:1904030 | negative regulation of cyclin-dependent protein kinase activity | 2/55 | 0.004159739 | 0.021084992 |
| BP | GO:0008645 | hexose transmembrane transport | 3/55 | 0.004272178 | 0.021571957 |
| BP | GO:0090050 | positive regulation of cell migration involved in sprouting angiogenesis | 2/55 | 0.004411493 | 0.022022285 |
| BP | GO:0140354 | lipid import into cell | 2/55 | 0.004411493 | 0.022022285 |
| BP | GO:2000758 | positive regulation of peptidyl-lysine acetylation | 2/55 | 0.004411493 | 0.022022285 |
| BP | GO:0015749 | monosaccharide transmembrane transport | 3/55 | 0.004488887 | 0.02232408 |
| BP | GO:0098657 | import into cell | 4/55 | 0.004516129 | 0.022375124 |
| BP | GO:0007569 | cell aging | 3/55 | 0.0045997 | 0.022619108 |
| BP | GO:0030218 | erythrocyte differentiation | 3/55 | 0.0045997 | 0.022619108 |
| BP | GO:0019362 | pyridine nucleotide metabolic process | 2/55 | 0.004670146 | 0.022795409 |
| BP | GO:0046496 | nicotinamide nucleotide metabolic process | 2/55 | 0.004670146 | 0.022795409 |
| BP | GO:0034219 | carbohydrate transmembrane transport | 3/55 | 0.004712162 | 0.022831373 |
| BP | GO:1904019 | epithelial cell apoptotic process | 3/55 | 0.004712162 | 0.022831373 |
| BP | GO:0045730 | respiratory burst | 2/55 | 0.004935656 | 0.023770486 |
| BP | GO:0043500 | muscle adaptation | 3/55 | 0.004942059 | 0.023770486 |
| BP | GO:0000096 | sulfur amino acid metabolic process | 2/55 | 0.005207984 | 0.024868025 |
| BP | GO:1902229 | regulation of intrinsic apoptotic signaling pathway in response to DNA damage | 2/55 | 0.005207984 | 0.024868025 |
| BP | GO:0042417 | dopamine metabolic process | 2/55 | 0.005487089 | 0.02601225 |
| BP | GO:1904706 | negative regulation of vascular associated smooth muscle cell proliferation | 2/55 | 0.005487089 | 0.02601225 |
| BP | GO:0046887 | positive regulation of hormone secretion | 3/55 | 0.00567203 | 0.026792611 |
| BP | GO:0016572 | histone phosphorylation | 2/55 | 0.00577293 | 0.027075137 |
| BP | GO:2000279 | negative regulation of DNA biosynthetic process | 2/55 | 0.00577293 | 0.027075137 |
| BP | GO:0030072 | peptide hormone secretion | 4/55 | 0.005802821 | 0.027118819 |
| BP | GO:0030968 | endoplasmic reticulum unfolded protein response | 3/55 | 0.006059997 | 0.028047882 |
| BP | GO:0045776 | negative regulation of blood pressure | 2/55 | 0.006065466 | 0.028047882 |
| BP | GO:0071634 | regulation of transforming growth factor beta production | 2/55 | 0.006065466 | 0.028047882 |
| BP | GO:0002790 | peptide secretion | 5/55 | 0.006164343 | 0.028405439 |
| BP | GO:1901657 | glycosyl compound metabolic process | 3/55 | 0.006327267 | 0.029054604 |
| BP | GO:0033574 | response to testosterone | 2/55 | 0.00636466 | 0.029124831 |
| BP | GO:0019751 | polyol metabolic process | 3/55 | 0.006463507 | 0.029373179 |
| BP | GO:0046683 | response to organophosphorus | 3/55 | 0.006463507 | 0.029373179 |
| BP | GO:0042326 | negative regulation of phosphorylation | 5/55 | 0.006555323 | 0.029688062 |
| BP | GO:0045616 | regulation of keratinocyte differentiation | 2/55 | 0.006670469 | 0.029901278 |
| BP | GO:0071604 | transforming growth factor beta production | 2/55 | 0.006670469 | 0.029901278 |
| BP | GO:0089718 | amino acid import across plasma membrane | 2/55 | 0.006670469 | 0.029901278 |
| BP | GO:0010595 | positive regulation of endothelial cell migration | 3/55 | 0.006741227 | 0.030116027 |
| BP | GO:0001894 | tissue homeostasis | 4/55 | 0.006937656 | 0.030573645 |
| BP | GO:0009069 | serine family amino acid metabolic process | 2/55 | 0.006982855 | 0.030573645 |
| BP | GO:0032309 | icosanoid secretion | 2/55 | 0.006982855 | 0.030573645 |
| BP | GO:0035094 | response to nicotine | 2/55 | 0.006982855 | 0.030573645 |
| BP | GO:0046189 | phenol-containing compound biosynthetic process | 2/55 | 0.006982855 | 0.030573645 |
| BP | GO:0090311 | regulation of protein deacetylation | 2/55 | 0.006982855 | 0.030573645 |
| BP | GO:0042542 | response to hydrogen peroxide | 3/55 | 0.00717099 | 0.031293412 |
| BP | GO:0034754 | cellular hormone metabolic process | 3/55 | 0.007317782 | 0.0318286 |
| BP | GO:1901985 | positive regulation of protein acetylation | 2/55 | 0.007627198 | 0.033065276 |
| BP | GO:0021700 | developmental maturation | 4/55 | 0.00770444 | 0.033290628 |
| BP | GO:0002686 | negative regulation of leukocyte migration | 2/55 | 0.007959077 | 0.033836212 |
| BP | GO:0034198 | cellular response to amino acid starvation | 2/55 | 0.007959077 | 0.033836212 |
| BP | GO:0045981 | positive regulation of nucleotide metabolic process | 2/55 | 0.007959077 | 0.033836212 |
| BP | GO:0048146 | positive regulation of fibroblast proliferation | 2/55 | 0.007959077 | 0.033836212 |
| BP | GO:1900544 | positive regulation of purine nucleotide metabolic process | 2/55 | 0.007959077 | 0.033836212 |
| BP | GO:0031056 | regulation of histone modification | 3/55 | 0.008078497 | 0.034123745 |
| BP | GO:0062013 | positive regulation of small molecule metabolic process | 3/55 | 0.008078497 | 0.034123745 |
| BP | GO:0033044 | regulation of chromosome organization | 4/55 | 0.008211018 | 0.034567566 |
| BP | GO:0010212 | response to ionizing radiation | 3/55 | 0.008236026 | 0.034567566 |
| BP | GO:0044282 | small molecule catabolic process | 5/55 | 0.008294769 | 0.034703595 |
| BP | GO:0042149 | cellular response to glucose starvation | 2/55 | 0.008642055 | 0.035928449 |
| BP | GO:0043090 | amino acid import | 2/55 | 0.008642055 | 0.035928449 |
| BP | GO:0051592 | response to calcium ion | 3/55 | 0.008719472 | 0.03613631 |
| BP | GO:0050708 | regulation of protein secretion | 4/55 | 0.008847285 | 0.036551066 |
| BP | GO:1901990 | regulation of mitotic cell cycle phase transition | 5/55 | 0.008939826 | 0.036693273 |
| BP | GO:0038066 | p38MAPK cascade | 2/55 | 0.008993076 | 0.036693273 |
| BP | GO:0072348 | sulfur compound transport | 2/55 | 0.008993076 | 0.036693273 |
| BP | GO:1990928 | response to amino acid starvation | 2/55 | 0.008993076 | 0.036693273 |
| BP | GO:0000077 | DNA damage checkpoint | 3/55 | 0.009219299 | 0.037500203 |
| BP | GO:1902001 | fatty acid transmembrane transport | 2/55 | 0.0093504 | 0.037612348 |
| BP | GO:1903202 | negative regulation of oxidative stress-induced cell death | 2/55 | 0.0093504 | 0.037612348 |
| BP | GO:0008203 | cholesterol metabolic process | 3/55 | 0.009389568 | 0.037612348 |
| BP | GO:0008643 | carbohydrate transport | 3/55 | 0.009389568 | 0.037612348 |
| BP | GO:0034620 | cellular response to unfolded protein | 3/55 | 0.009389568 | 0.037612348 |
| BP | GO:0015908 | fatty acid transport | 3/55 | 0.009561674 | 0.037955664 |
| BP | GO:0043535 | regulation of blood vessel endothelial cell migration | 3/55 | 0.009561674 | 0.037955664 |
| BP | GO:1903364 | positive regulation of cellular protein catabolic process | 3/55 | 0.009561674 | 0.037955664 |
| BP | GO:0016570 | histone modification | 5/55 | 0.009706294 | 0.03832939 |
| BP | GO:0071715 | icosanoid transport | 2/55 | 0.00971399 | 0.03832939 |
| BP | GO:1905039 | carboxylic acid transmembrane transport | 3/55 | 0.010268548 | 0.040396613 |
| BP | GO:1903825 | organic acid transmembrane transport | 3/55 | 0.010449898 | 0.040663514 |
| BP | GO:0006984 | ER-nucleus signaling pathway | 2/55 | 0.010459812 | 0.040663514 |
| BP | GO:0010332 | response to gamma radiation | 2/55 | 0.010459812 | 0.040663514 |
| BP | GO:0045599 | negative regulation of fat cell differentiation | 2/55 | 0.010459812 | 0.040663514 |
| BP | GO:0031570 | DNA integrity checkpoint | 3/55 | 0.010633107 | 0.041215636 |
| BP | GO:0016569 | covalent chromatin modification | 5/55 | 0.010892014 | 0.04209539 |
| BP | GO:1902652 | secondary alcohol metabolic process | 3/55 | 0.011005121 | 0.042408159 |
| BP | GO:0034614 | cellular response to reactive oxygen species | 3/55 | 0.011193931 | 0.042775351 |
| BP | GO:0051100 | negative regulation of binding | 3/55 | 0.011193931 | 0.042775351 |
| BP | GO:0046164 | alcohol catabolic process | 2/55 | 0.011230238 | 0.042775351 |
| BP | GO:0071385 | cellular response to glucocorticoid stimulus | 2/55 | 0.011230238 | 0.042775351 |
| BP | GO:0048660 | regulation of smooth muscle cell proliferation | 3/55 | 0.011384615 | 0.043114149 |
| BP | GO:2001242 | regulation of intrinsic apoptotic signaling pathway | 3/55 | 0.011384615 | 0.043114149 |
| BP | GO:1901214 | regulation of neuron death | 4/55 | 0.011583608 | 0.043742052 |
| BP | GO:0016049 | cell growth | 5/55 | 0.01176897 | 0.044198721 |
| BP | GO:0048659 | smooth muscle cell proliferation | 3/55 | 0.011771616 | 0.044198721 |
| BP | GO:0007568 | aging | 4/55 | 0.01184452 | 0.044346111 |
| BP | GO:0010043 | response to zinc ion | 2/55 | 0.012024967 | 0.044894167 |
| BP | GO:2001252 | positive regulation of chromosome organization | 3/55 | 0.01216615 | 0.045292951 |
| BP | GO:0023061 | signal release | 5/55 | 0.012276489 | 0.045574987 |
| BP | GO:0016125 | sterol metabolic process | 3/55 | 0.012366248 | 0.045779252 |
| BP | GO:1901987 | regulation of cell cycle phase transition | 5/55 | 0.012588073 | 0.046469904 |
| BP | GO:0051055 | negative regulation of lipid biosynthetic process | 2/55 | 0.012843701 | 0.047149432 |
| BP | GO:1902041 | regulation of extrinsic apoptotic signaling pathway via death domain receptors | 2/55 | 0.012843701 | 0.047149432 |
| BP | GO:0035967 | cellular response to topologically incorrect protein | 3/55 | 0.012977907 | 0.047509765 |
| BP | GO:0016239 | positive regulation of macroautophagy | 2/55 | 0.013261977 | 0.048281464 |
| BP | GO:0032722 | positive regulation of chemokine production | 2/55 | 0.013261977 | 0.048281464 |
| BP | GO:0070059 | intrinsic apoptotic signaling pathway in response to endoplasmic reticulum stress | 2/55 | 0.013686144 | 0.04968842 |
| CC | GO:0043020 | NADPH oxidase complex | 4/55 | 2.75E-08 | 3.21E-06 |
| CC | GO:1990204 | oxidoreductase complex | 4/55 | 0.000270143 | 0.015782058 |
| CC | GO:0045177 | apical part of cell | 6/55 | 0.001057789 | 0.041198109 |
| CC | GO:0097038 | perinuclear endoplasmic reticulum | 2/55 | 0.00143361 | 0.0418765 |
| CC | GO:0005902 | microvillus | 3/55 | 0.002158006 | 0.047239551 |
| CC | GO:0031528 | microvillus membrane | 2/55 | 0.002425815 | 0.047239551 |
| MF | GO:0016651 | oxidoreductase activity, acting on NAD(P)H | 9/55 | 1.83E-11 | 3.14E-09 |
| MF | GO:0016175 | superoxide-generating NAD(P)H oxidase activity | 4/55 | 2.35E-08 | 2.02E-06 |
| MF | GO:0050664 | oxidoreductase activity, acting on NAD(P)H, oxygen as acceptor | 4/55 | 1.28E-07 | 7.34E-06 |
| MF | GO:0016705 | oxidoreductase activity, acting on paired donors, with incorporation or reduction of molecular oxygen | 7/55 | 4.82E-07 | 2.07E-05 |
| MF | GO:0016701 | oxidoreductase activity, acting on single donors with incorporation of molecular oxygen | 4/55 | 8.74E-07 | 3.00E-05 |
| MF | GO:0004033 | aldo-keto reductase (NADP) activity | 4/55 | 1.21E-06 | 3.45E-05 |
| MF | GO:0032052 | bile acid binding | 3/55 | 4.14E-06 | 0.000101525 |
| MF | GO:0004032 | alditol:NADP+ 1-oxidoreductase activity | 3/55 | 5.51E-06 | 0.000118194 |
| MF | GO:0016709 | oxidoreductase activity, acting on paired donors, with incorporation or reduction of molecular oxygen, NAD(P)H as one donor, and incorporation of one atom of oxygen | 4/55 | 1.28E-05 | 0.000243587 |
| MF | GO:0004303 | estradiol 17-beta-dehydrogenase activity | 3/55 | 2.02E-05 | 0.000346266 |
| MF | GO:0016616 | oxidoreductase activity, acting on the CH-OH group of donors, NAD or NADP as acceptor | 5/55 | 2.96E-05 | 0.000461135 |
| MF | GO:0008106 | alcohol dehydrogenase (NADP+) activity | 3/55 | 3.27E-05 | 0.000467326 |
| MF | GO:0016614 | oxidoreductase activity, acting on CH-OH group of donors | 5/55 | 4.34E-05 | 0.000572906 |
| MF | GO:0016702 | oxidoreductase activity, acting on single donors with incorporation of molecular oxygen, incorporation of two atoms of oxygen | 3/55 | 4.94E-05 | 0.000602609 |
| MF | GO:0004860 | protein kinase inhibitor activity | 4/55 | 5.42E-05 | 0.000602609 |
| MF | GO:0033293 | monocarboxylic acid binding | 4/55 | 6.06E-05 | 0.000602609 |
| MF | GO:0020037 | heme binding | 5/55 | 6.18E-05 | 0.000602609 |
| MF | GO:0016628 | oxidoreductase activity, acting on the CH-CH group of donors, NAD or NADP as acceptor | 3/55 | 6.32E-05 | 0.000602609 |
| MF | GO:0019210 | kinase inhibitor activity | 4/55 | 6.76E-05 | 0.000610552 |
| MF | GO:0005506 | iron ion binding | 5/55 | 8.57E-05 | 0.000700473 |
| MF | GO:0046906 | tetrapyrrole binding | 5/55 | 8.57E-05 | 0.000700473 |
| MF | GO:0033764 | steroid dehydrogenase activity, acting on the CH-OH group of donors, NAD or NADP as acceptor | 3/55 | 9.79E-05 | 0.000757391 |
| MF | GO:0050660 | flavin adenine dinucleotide binding | 4/55 | 0.000101528 | 0.000757391 |
| MF | GO:0030291 | protein serine/threonine kinase inhibitor activity | 3/55 | 0.000119074 | 0.000851277 |
| MF | GO:0016229 | steroid dehydrogenase activity | 3/55 | 0.000143049 | 0.00096596 |
| MF | GO:0051213 | dioxygenase activity | 4/55 | 0.000146375 | 0.00096596 |
| MF | GO:0031406 | carboxylic acid binding | 5/55 | 0.000223227 | 0.001418559 |
| MF | GO:0004497 | monooxygenase activity | 4/55 | 0.000238343 | 0.001460523 |
| MF | GO:0016829 | lyase activity | 5/55 | 0.000285142 | 0.001687047 |
| MF | GO:0030283 | testosterone dehydrogenase [NAD(P)] activity | 2/55 | 0.000391416 | 0.002238627 |
| MF | GO:0004861 | cyclin-dependent protein serine/threonine kinase inhibitor activity | 2/55 | 0.000571871 | 0.003118762 |
| MF | GO:0030170 | pyridoxal phosphate binding | 3/55 | 0.000599836 | 0.003118762 |
| MF | GO:0070279 | vitamin B6 binding | 3/55 | 0.000599836 | 0.003118762 |
| MF | GO:0016627 | oxidoreductase activity, acting on the CH-CH group of donors | 3/55 | 0.000737014 | 0.003719294 |
| MF | GO:0016655 | oxidoreductase activity, acting on NAD(P)H, quinone or similar compound as acceptor | 3/55 | 0.000774163 | 0.003795144 |
| MF | GO:0016836 | hydro-lyase activity | 3/55 | 0.000934622 | 0.004454484 |
| MF | GO:0015172 | acidic amino acid transmembrane transporter activity | 2/55 | 0.001031791 | 0.004658781 |
| MF | GO:0035173 | histone kinase activity | 2/55 | 0.001031791 | 0.004658781 |
| MF | GO:0016835 | carbon-oxygen lyase activity | 3/55 | 0.001718541 | 0.007484019 |
| MF | GO:0008483 | transaminase activity | 2/55 | 0.001788359 | 0.007484019 |
| MF | GO:0072349 | modified amino acid transmembrane transporter activity | 2/55 | 0.001788359 | 0.007484019 |
| MF | GO:0016769 | transferase activity, transferring nitrogenous groups | 2/55 | 0.002146284 | 0.008744166 |
| MF | GO:0016209 | antioxidant activity | 3/55 | 0.002191406 | 0.008744166 |
| MF | GO:0019887 | protein kinase regulator activity | 4/55 | 0.002522039 | 0.009834744 |
| MF | GO:0051019 | mitogen-activated protein kinase binding | 2/55 | 0.004135679 | 0.015768786 |
| MF | GO:0019207 | kinase regulator activity | 4/55 | 0.004261928 | 0.015896895 |
| MF | GO:0015175 | neutral amino acid transmembrane transporter activity | 2/55 | 0.004659752 | 0.017010967 |
| MF | GO:0019825 | oxygen binding | 2/55 | 0.006096189 | 0.021791202 |
| MF | GO:0004712 | protein serine/threonine/tyrosine kinase activity | 2/55 | 0.007708607 | 0.026992543 |
| MF | GO:0009055 | electron transfer activity | 3/55 | 0.008224629 | 0.028223465 |
| MF | GO:1901682 | sulfur compound transmembrane transporter activity | 2/55 | 0.009121633 | 0.030687845 |
| MF | GO:0019842 | vitamin binding | 3/55 | 0.009411624 | 0.03105455 |
| MF | GO:0016538 | cyclin-dependent protein serine/threonine kinase regulator activity | 2/55 | 0.009868246 | 0.031946854 |
| MF | GO:0050661 | NADP binding | 2/55 | 0.011037355 | 0.035069958 |
| MF | GO:0015179 | L-amino acid transmembrane transporter activity | 2/55 | 0.013548597 | 0.042266436 |
| MF | GO:0005507 | copper ion binding | 2/55 | 0.013989094 | 0.042861323 |
| MF | GO:0008514 | organic anion transmembrane transporter activity | 3/55 | 0.016070256 | 0.047627091 |
| MF | GO:0046982 | protein heterodimerization activity | 4/55 | 0.01609971 | 0.047627091 |

**Table 3b. KEGG enrichment analysis**.

| ID | Description | GeneRatio | pvalue | qvalue | Count |
| --- | --- | --- | --- | --- | --- |
| hsa00590 | Arachidonic acid metabolism | 4/42 | 0.000263587 | 0.024394262 | 4 |
| hsa01230 | Biosynthesis of amino acids | 4/42 | 0.000582525 | 0.024394262 | 4 |
| hsa05140 | Leishmaniasis | 4/42 | 0.000643737 | 0.024394262 | 4 |
| hsa05208 | Chemical carcinogenesis - reactive oxygen species | 6/42 | 0.000934012 | 0.026545591 | 6 |
| hsa04216 | Ferroptosis | 3/42 | 0.001206723 | 0.027437079 | 3 |
| hsa04913 | Ovarian steroidogenesis | 3/42 | 0.002274106 | 0.036033972 | 3 |
| hsa04066 | HIF-1 signaling pathway | 4/42 | 0.002347657 | 0.036033972 | 4 |
| hsa01200 | Carbon metabolism | 4/42 | 0.002852689 | 0.036033972 | 4 |
| hsa04726 | Serotonergic synapse | 4/42 | 0.002852689 | 0.036033972 | 4 |
| hsa00480 | Glutathione metabolism | 3/42 | 0.00328581 | 0.037354475 | 3 |
| hsa00140 | Steroid hormone biosynthesis | 3/42 | 0.003791467 | 0.03918454 | 3 |
| hsa01210 | 2-Oxocarboxylic acid metabolism | 2/42 | 0.004248548 | 0.040249406 | 2 |
| hsa05418 | Fluid shear stress and atherosclerosis | 4/42 | 0.005618191 | 0.04614813 | 4 |
| hsa00220 | Arginine biosynthesis | 2/42 | 0.005683057 | 0.04614813 | 2 |
| hsa04115 | p53 signaling pathway | 3/42 | 0.006276262 | 0.047567457 | 3 |

# Appendix 4

**945 ferroptosis-related lncRNAs**

**Table 4. 945** ferroptosis-related lncRNAs

| gene | conMean | treatMean | logFC | pValue |
| --- | --- | --- | --- | --- |
| LMO7-AS1 | 3.127761867 | 1.291154485 | -1.276469049 | 9.89E-08 |
| AC005332.1 | 0.127834333 | 0.276861109 | 1.11488705 | 1.28E-05 |
| AC105219.1 | 0.037109717 | 0.352959969 | 3.249635656 | 8.37E-08 |
| MYG1-AS1 | 0.379552421 | 1.292246879 | 1.76751066 | 3.38E-14 |
| LINC02541 | 0.027370393 | 0.25975353 | 3.246455303 | 2.30E-09 |
| LINC00174 | 1.224261916 | 3.104545847 | 1.342469999 | 2.62E-06 |
| LUCAT1 | 0.015730351 | 0.319393324 | 4.343711369 | 2.56E-13 |
| AC145423.1 | 0.840275378 | 1.756528568 | 1.063792925 | 0.003347879 |
| LINC01559 | 0.187711872 | 0.867811468 | 2.208861753 | 0.003938549 |
| FOXD2-AS1 | 0.508657073 | 1.633913469 | 1.683566331 | 1.07E-13 |
| AC093726.2 | 0.973978986 | 3.014236631 | 1.629830129 | 6.15E-09 |
| AP003119.3 | 2.183659943 | 0.914276426 | -1.256045879 | 9.64E-05 |
| LINC02340 | 0.102899053 | 0.212601461 | 1.046921807 | 0.00082768 |
| N4BP2L2-IT2 | 0.196385325 | 0.45612051 | 1.215727913 | 3.21E-05 |
| LINC02061 | 7.538900671 | 0.176369011 | -5.417685164 | 1.14E-19 |
| AC092535.5 | 0.879592076 | 21.90031572 | 4.63797325 | 5.46E-15 |
| AC144548.1 | 0.101422393 | 0.232738145 | 1.198331471 | 0.000100275 |
| AC079848.1 | 1.36369023 | 0.499765315 | -1.448193282 | 7.51E-14 |
| AC005962.1 | 0.366239174 | 0.855219001 | 1.223507794 | 5.08E-05 |
| AC124017.1 | 2.87723592 | 0.027021878 | -6.734411763 | 5.82E-26 |
| AL157935.1 | 0.522519793 | 0.235419388 | -1.150252547 | 6.22E-13 |
| AC079322.1 | 0.184564926 | 0.383110021 | 1.053630345 | 9.80E-05 |
| AC007038.1 | 0.616589604 | 1.492264401 | 1.275120706 | 2.09E-06 |
| AP001505.1 | 2.345672471 | 6.264848996 | 1.417278154 | 5.42E-08 |
| AC021218.1 | 1.378428626 | 3.359183804 | 1.28508617 | 0.00292031 |
| IDH1-AS1 | 0.599547199 | 1.227272055 | 1.033509853 | 5.07E-07 |
| TMEM246-AS1 | 4.34998335 | 0.82236163 | -2.403165021 | 1.05E-12 |
| KLHL6-AS1 | 0.416976745 | 0.20276998 | -1.04012285 | 0.000146251 |
| AC135050.3 | 0.326093661 | 1.174954177 | 1.84924619 | 3.51E-07 |
| AC093278.2 | 2.401569838 | 0.625730642 | -1.940364103 | 2.22E-18 |
| DICER1-AS1 | 1.409680024 | 3.283466799 | 1.219852138 | 3.69E-05 |
| ZFAND2A-DT | 1.591259613 | 3.570574765 | 1.165987098 | 8.37E-10 |
| AL355488.1 | 0.933476884 | 2.259324202 | 1.275205103 | 8.77E-06 |
| TTC21B-AS1 | 0.021901639 | 0.379824851 | 4.116223552 | 0.005210886 |
| AL096799.1 | 0.113767608 | 0.959553396 | 3.076273242 | 0.001021255 |
| AC008870.2 | 0.254629537 | 0.66874507 | 1.393056568 | 5.13E-07 |
| GAS6-DT | 1.727899289 | 0.374840277 | -2.204671245 | 1.34E-16 |
| AC092115.3 | 0.097111821 | 0.224760275 | 1.21066824 | 0.000521331 |
| AC007342.4 | 1.672075379 | 0.471317059 | -1.826870083 | 1.44E-12 |
| AC022306.3 | 0.235713087 | 0.630422427 | 1.419286999 | 4.03E-07 |
| AP001628.1 | 0.190257644 | 0.458788374 | 1.269874417 | 0.003272321 |
| AC010173.1 | 0.315763312 | 0.638839601 | 1.016610192 | 1.44E-06 |
| LRRK2-DT | 3.969189441 | 15.08708062 | 1.926397342 | 2.33E-12 |
| AC048382.2 | 0.315173617 | 0.788194981 | 1.322405791 | 0.001684362 |
| CD44-AS1 | 0.062224811 | 0.237450208 | 1.932063163 | 0.006827109 |
| AL732292.2 | 0.171674386 | 0.383858266 | 1.160898912 | 2.13E-06 |
| AC004923.4 | 0.221304198 | 0.891736263 | 2.010586269 | 6.98E-11 |
| AC087623.1 | 0.285544972 | 1.191523945 | 2.061018057 | 1.26E-05 |
| AP001160.1 | 0.278050902 | 0.56151112 | 1.013965576 | 0.000340654 |
| LINC02471 | 3.060377308 | 30.79614779 | 3.330968463 | 4.91E-13 |
| AC010618.2 | 0.219040438 | 0.438763785 | 1.002247214 | 0.000251571 |
| AL138826.1 | 14.03044089 | 0.606924668 | -4.530899077 | 2.87E-20 |
| AC022144.1 | 1.49829551 | 3.799464168 | 1.342473776 | 1.77E-07 |
| AP001439.1 | 0.108768545 | 0.222333234 | 1.031462219 | 0.00385336 |
| AL133215.1 | 0.106487419 | 0.221889178 | 1.059156316 | 0.022147847 |
| AC141002.1 | 0.268972254 | 0.919040613 | 1.772671257 | 9.23E-09 |
| ITGB2-AS1 | 0.265500196 | 1.178372518 | 2.150010859 | 4.21E-10 |
| AC093582.1 | 0.181649679 | 0.459388766 | 1.338556766 | 0.003789509 |
| AC025171.2 | 0.435956527 | 1.582322782 | 1.859787744 | 1.44E-13 |
| MAP3K5-AS1 | 0.121315245 | 0.349266126 | 1.525565874 | 5.89E-06 |
| LINC02568 | 1.667456642 | 0.205135602 | -3.022999446 | 7.37E-18 |
| AL390728.5 | 2.448778486 | 6.963423528 | 1.507734498 | 2.06E-09 |
| LINC02732 | 1.953087803 | 0.514603263 | -1.924224298 | 8.47E-10 |
| MMP25-AS1 | 0.483214541 | 1.680516528 | 1.798168958 | 2.20E-13 |
| AC138150.2 | 0.294550683 | 1.069436704 | 1.860263296 | 1.34E-10 |
| ZKSCAN2-DT | 0.458817892 | 1.14920177 | 1.324638563 | 1.73E-06 |
| AC092757.3 | 0.290533115 | 0.895064047 | 1.623288307 | 1.91E-07 |
| LINC01780 | 1.058107705 | 0.35534051 | -1.574212412 | 1.42E-12 |
| ADAMTS9-AS1 | 14.51360457 | 33.83460945 | 1.221093867 | 3.58E-06 |
| LINC01117 | 1.400418834 | 0.656720839 | -1.092506229 | 6.15E-10 |
| AP000346.1 | 0.151398335 | 0.429634789 | 1.504761481 | 0.0261272 |
| AC018638.7 | 0.389269439 | 1.18058251 | 1.600657885 | 0.006312318 |
| AC145098.1 | 0.216298799 | 0.633594027 | 1.550533081 | 7.53E-08 |
| ZEB1-AS1 | 1.928888925 | 4.138108831 | 1.10120152 | 7.96E-10 |
| AC124798.1 | 1.340146078 | 3.20062823 | 1.255964845 | 1.37E-09 |
| LINC01802 | 1.835016238 | 0.39840425 | -2.203487887 | 4.58E-37 |
| AL135791.1 | 0.303324342 | 0.651255442 | 1.102362244 | 8.80E-05 |
| LINC01836 | 5.199569545 | 2.327463282 | -1.159633784 | 6.67E-05 |
| LINC01788 | 1.454837552 | 0.110084542 | -3.724174266 | 1.34E-22 |
| FSIP2-AS1 | 0.0983569 | 0.24610921 | 1.323200482 | 0.000512372 |
| PPP4R1-AS1 | 0.166078154 | 0.573452829 | 1.787812501 | 3.28E-06 |
| AC092119.2 | 0.284223231 | 1.180692698 | 2.054537135 | 2.67E-08 |
| AL161669.3 | 1.293503975 | 4.434645084 | 1.777534158 | 7.76E-07 |
| IGBP1-AS1 | 0.13722796 | 0.345056176 | 1.330256802 | 6.89E-05 |
| AC131159.2 | 0.265090691 | 0.593961857 | 1.163884276 | 2.45E-09 |
| LINC02615 | 0.585066003 | 2.451456829 | 2.066968061 | 2.75E-10 |
| USP46-DT | 3.399821331 | 1.169216825 | -1.539916437 | 1.80E-18 |
| AC004830.2 | 0.153819401 | 0.410517523 | 1.416206325 | 0.007115663 |
| LINC01191 | 0.142569339 | 0.456974901 | 1.680451181 | 0.000725405 |
| AL139041.1 | 0.078262197 | 0.276328008 | 1.819994274 | 8.44E-06 |
| AC018809.1 | 0.27049227 | 0.544587792 | 1.009577278 | 0.001662629 |
| LINC01410 | 0.253633654 | 0.517900067 | 1.029927561 | 0.000174817 |
| AC022558.3 | 0.141489324 | 0.361546944 | 1.353489779 | 1.22E-05 |
| RASGRP3-AS1 | 0.274772098 | 0.91736838 | 1.739265669 | 1.43E-06 |
| HMGA1P4 | 0.630249577 | 1.705274544 | 1.436008878 | 1.20E-05 |
| AC005154.4 | 0.11902681 | 0.291550068 | 1.292457095 | 0.000680813 |
| SOCS2-AS1 | 0.979291107 | 0.34718067 | -1.496051157 | 4.96E-15 |
| C3orf35 | 0.097126886 | 0.259841531 | 1.419689424 | 4.20E-07 |
| SEMA3B-AS1 | 7.096221572 | 3.015586309 | -1.23461253 | 1.35E-09 |
| AC106820.3 | 0.668400379 | 1.513390051 | 1.178999409 | 5.72E-05 |
| AC006441.4 | 2.178847103 | 0.112899995 | -4.270447629 | 5.69E-14 |
| AL353152.1 | 3.363700013 | 0.249037303 | -3.755615285 | 7.19E-19 |
| MAFG-DT | 1.536855269 | 3.759641948 | 1.290613965 | 2.07E-08 |
| AC015849.3 | 0.691357039 | 1.496131775 | 1.113734388 | 0.001225148 |
| STAG3L5P-PVRIG2P-PILRB | 0.578068157 | 1.816332041 | 1.651716454 | 9.15E-07 |
| AP006545.2 | 0.151760078 | 0.33607445 | 1.146988544 | 0.003115244 |
| AC004076.2 | 0.130260209 | 0.273904982 | 1.072279057 | 0.000684896 |
| AC068792.1 | 0.249868839 | 0.893362322 | 1.838074413 | 3.04E-11 |
| SNHG22 | 0.237165509 | 0.514514986 | 1.117318884 | 1.00E-06 |
| AC002553.1 | 0.669246434 | 1.577860546 | 1.237360252 | 1.87E-06 |
| AF111167.2 | 1.084142587 | 0.312123966 | -1.796363472 | 1.22E-17 |
| LINC01150 | 0.243546033 | 0.631878241 | 1.375452106 | 3.44E-06 |
| AF106564.1 | 0.270669937 | 0.553694684 | 1.032556015 | 0.020580829 |
| AC005840.2 | 0.265489488 | 0.722914048 | 1.445169384 | 6.65E-07 |
| LINC01588 | 0.158148263 | 0.351369259 | 1.151710263 | 3.40E-08 |
| AC025048.4 | 0.203798303 | 0.658236916 | 1.691464899 | 4.53E-14 |
| LINC00621 | 0.694039964 | 3.142594926 | 2.178865681 | 0.000167937 |
| ZMIZ1-AS1 | 0.13858965 | 0.553614529 | 1.998062288 | 4.19E-12 |
| AC009120.3 | 0.711158824 | 1.6029031 | 1.172443513 | 0.000758661 |
| GK-AS1 | 0.255470685 | 0.614440257 | 1.266114987 | 3.95E-05 |
| AL022069.1 | 0.615183286 | 0.240696967 | -1.353798343 | 3.32E-06 |
| LINC01275 | 0.052848417 | 0.245249498 | 2.214318012 | 2.80E-10 |
| AC084357.2 | 0.188846276 | 0.427114478 | 1.177410468 | 6.87E-06 |
| LINC02522 | 0.011615142 | 0.628733799 | 5.758370679 | 1.41E-07 |
| AC087741.1 | 0.836500565 | 2.43670294 | 1.542491963 | 3.12E-07 |
| LINC02285 | 0.098432928 | 0.303594136 | 1.624931017 | 5.09E-08 |
| AC016737.1 | 0.182017582 | 0.50751639 | 1.479376599 | 4.50E-05 |
| AC009318.3 | 0.644611599 | 1.368593644 | 1.086192098 | 2.71E-10 |
| MIR210HG | 1.66348086 | 5.768056642 | 1.793880065 | 3.75E-09 |
| F11-AS1 | 2.146862157 | 0.067705885 | -4.986804524 | 6.01E-20 |
| AC005899.7 | 0.177716286 | 0.522826431 | 1.556756179 | 2.83E-05 |
| AC005324.4 | 0.093759638 | 0.287537401 | 1.616710718 | 4.10E-05 |
| LINC01569 | 1.620439955 | 4.517241955 | 1.479056627 | 1.05E-09 |
| LINC02683 | 0.041984152 | 0.315761242 | 2.910917336 | 8.89E-09 |
| AC005387.2 | 0.173021197 | 0.349566233 | 1.014617035 | 0.000688742 |
| AC113139.1 | 0.237288451 | 0.515060027 | 1.118098694 | 0.003523129 |
| HOXB-AS4 | 0.598152356 | 1.821721982 | 1.606717894 | 4.42E-05 |
| HOXB-AS2 | 0.977511238 | 0.460700884 | -1.085282923 | 7.56E-07 |
| AC156455.1 | 0.242939785 | 1.53095684 | 2.655762934 | 7.99E-15 |
| LINC00857 | 0.651810147 | 2.801770146 | 2.103814888 | 3.09E-10 |
| AL121782.1 | 0.117246818 | 0.398841174 | 1.766265583 | 1.02E-06 |
| DLG3-AS1 | 0.189071962 | 0.509816883 | 1.431043713 | 2.67E-08 |
| AC004223.4 | 0.161549863 | 0.358372776 | 1.14948152 | 0.000391054 |
| B3GALT5-AS1 | 0.104166913 | 0.360221691 | 1.789987958 | 0.032287917 |
| AL162741.1 | 0.097639524 | 0.346284751 | 1.826421697 | 6.68E-06 |
| AC133528.1 | 0.196413574 | 0.617613632 | 1.652809964 | 2.02E-05 |
| AC136475.3 | 4.277550815 | 47.26356163 | 3.465871452 | 2.17E-07 |
| AL683807.1 | 0.065715229 | 0.282326528 | 2.103065051 | 1.67E-05 |
| COLCA1 | 3.008067121 | 0.934267263 | -1.686929538 | 8.09E-16 |
| LINC02804 | 0.103817978 | 0.358792629 | 1.789093952 | 4.89E-05 |
| LNCTAM34A | 0.360347675 | 1.876156123 | 2.380318442 | 1.03E-15 |
| GATA3-AS1 | 2.514899185 | 0.325203544 | -2.951085683 | 3.98E-19 |
| VPS9D1-AS1 | 0.441269225 | 1.45227711 | 1.718585721 | 5.07E-07 |
| PTPRN2-AS1 | 0.119421822 | 0.931837883 | 2.964012497 | 4.88E-15 |
| AC005670.1 | 0.146260444 | 0.29478178 | 1.011107707 | 0.010197676 |
| AL157394.1 | 0.339571276 | 1.229264997 | 1.856009624 | 8.88E-14 |
| AL391840.1 | 0.572433195 | 0.185433468 | -1.626205683 | 3.09E-10 |
| AC092809.2 | 0.164921605 | 0.330857231 | 1.004428403 | 0.00215247 |
| AL512652.1 | 0.11428064 | 0.3419189 | 1.581073149 | 5.48E-06 |
| RRN3P2 | 0.175199277 | 0.401495649 | 1.196387534 | 3.41E-06 |
| AC010245.2 | 0.470807119 | 0.989534263 | 1.071613528 | 6.58E-07 |
| AL161668.4 | 1.561737387 | 0.121319811 | -3.686264816 | 6.49E-18 |
| AL359881.1 | 0.400880262 | 1.467500032 | 1.872117245 | 5.01E-05 |
| AC005479.2 | 0.210241066 | 0.470149317 | 1.161074526 | 1.24E-05 |
| AC130371.2 | 6.113759174 | 2.901886556 | -1.075068604 | 1.02E-08 |
| ANKRD44-AS1 | 0.271151557 | 0.845683255 | 1.641017959 | 0.005292228 |
| AC012645.3 | 0.092867311 | 0.273427327 | 1.557914667 | 1.09E-07 |
| AL359504.1 | 0.31705585 | 0.83487679 | 1.396826308 | 1.80E-06 |
| AC087477.2 | 0.482953271 | 1.56046534 | 1.692020805 | 1.44E-05 |
| AC007497.1 | 0.07314019 | 0.25061022 | 1.776708972 | 2.71E-07 |
| AC004921.1 | 0.134062352 | 0.640371727 | 2.256005465 | 7.07E-11 |
| AL031714.1 | 0.376359854 | 0.820561299 | 1.124498368 | 1.22E-05 |
| LINC01606 | 2.434996841 | 0.15507203 | -3.972909504 | 6.32E-22 |
| AC011005.4 | 0.297922206 | 1.144492937 | 1.941700994 | 4.99E-07 |
| AL035661.1 | 23.09297819 | 1.350139786 | -4.096273554 | 9.44E-19 |
| LINC02019 | 0.209524195 | 0.594463785 | 1.504472073 | 7.84E-08 |
| AC118755.2 | 0.138956762 | 0.432778118 | 1.638991515 | 0.000129388 |
| DDX11-AS1 | 0.108363195 | 0.456859701 | 2.075876349 | 3.96E-17 |
| MMP2-AS1 | 0.055747402 | 0.801530958 | 3.845781773 | 7.36E-12 |
| COL18A1-AS1 | 1.461716542 | 0.189907733 | -2.944293013 | 1.62E-17 |
| AC124312.3 | 0.931695124 | 0.399247741 | -1.222573698 | 3.04E-10 |
| NR4A1AS | 1.811753006 | 0.509409476 | -1.830488587 | 2.46E-14 |
| LINC01679 | 1.062796562 | 0.187301747 | -2.504429204 | 7.58E-16 |
| AC005696.3 | 0.142295127 | 0.307683541 | 1.112561011 | 0.003405075 |
| AP003717.1 | 0.04775203 | 0.69347582 | 3.860211601 | 7.00E-10 |
| AP000345.2 | 0.545143512 | 1.901687781 | 1.802572421 | 0.012911795 |
| AC092078.2 | 2.452729428 | 0.041586999 | -5.882111698 | 1.69E-37 |
| AC027319.1 | 0.103687802 | 0.212854946 | 1.037624434 | 3.45E-05 |
| LINC01126 | 0.215994571 | 0.452053228 | 1.065497608 | 0.00039096 |
| SLFNL1-AS1 | 0.119152635 | 0.317284982 | 1.412968382 | 4.03E-06 |
| MIR34AHG | 0.136559155 | 0.331762199 | 1.280623481 | 1.86E-06 |
| AC093583.1 | 10.06008078 | 0.164794194 | -5.931832662 | 3.46E-20 |
| AC127024.5 | 1.031947588 | 2.260990554 | 1.131585266 | 5.74E-06 |
| AC018648.1 | 0.111257982 | 0.361156572 | 1.698715575 | 0.000224256 |
| AC019257.1 | 0.549157121 | 2.914057707 | 2.407738559 | 2.98E-09 |
| AC063919.1 | 3.916913912 | 1.277980173 | -1.615851967 | 3.88E-09 |
| AC109347.2 | 0.264618516 | 0.64467127 | 1.284649676 | 3.07E-08 |
| AC092295.2 | 1.788113003 | 0.871333684 | -1.037140691 | 5.82E-15 |
| AC103691.1 | 0.800663155 | 1.739187475 | 1.119146133 | 0.001351329 |
| AC090948.3 | 0.372010717 | 0.835977521 | 1.168119967 | 1.29E-05 |
| CASC19 | 0.037348288 | 0.219434848 | 2.554678653 | 9.59E-05 |
| AC008514.1 | 0.764158489 | 0.310135287 | -1.300974205 | 2.41E-13 |
| LINC02585 | 0.430143601 | 1.547155507 | 1.84672793 | 1.20E-13 |
| AC097641.2 | 0.231338366 | 0.646516637 | 1.482682951 | 2.60E-08 |
| TMEM51-AS1 | 0.616506781 | 1.309125105 | 1.086414305 | 0.002067299 |
| MACORIS | 0.191380662 | 0.80088678 | 2.065153241 | 1.67E-09 |
| AC136475.5 | 0.136179011 | 0.446066313 | 1.711753833 | 1.42E-06 |
| HIF1A-AS3 | 0.253674905 | 0.562126981 | 1.147915257 | 0.001165711 |
| GAPLINC | 0.058663971 | 0.33670787 | 2.520950817 | 2.42E-09 |
| AL139280.1 | 6.452837171 | 0.444416523 | -3.85994926 | 1.13E-22 |
| AC087289.2 | 0.191941182 | 0.546973938 | 1.51080781 | 3.29E-08 |
| AC006157.1 | 0.453334188 | 0.177859362 | -1.34983805 | 8.57E-05 |
| AL138976.2 | 0.905716509 | 2.071604551 | 1.193617174 | 1.93E-07 |
| AL365181.3 | 0.376904907 | 10.01205671 | 4.731393982 | 6.83E-17 |
| AC087762.1 | 1.942907556 | 0.076119491 | -4.673807525 | 2.10E-22 |
| AC090197.1 | 0.100485079 | 0.239194776 | 1.251204595 | 3.41E-06 |
| AL590666.2 | 0.433902131 | 5.686747922 | 3.712162278 | 2.34E-16 |
| AC010761.1 | 0.515721475 | 1.13794545 | 1.141767374 | 5.81E-07 |
| AC005363.2 | 0.109116853 | 0.239940821 | 1.136804686 | 0.004167847 |
| AC244153.1 | 1.361985952 | 0.448345458 | -1.603029135 | 3.45E-17 |
| AC092338.1 | 0.179037439 | 0.50007559 | 1.481884882 | 8.12E-05 |
| YEATS2-AS1 | 0.110881939 | 0.228291015 | 1.041849689 | 0.000137235 |
| AP001189.3 | 2.882483106 | 0.583330559 | -2.304926593 | 1.21E-17 |
| AL928921.1 | 0.673801342 | 0.28615309 | -1.235536119 | 1.58E-21 |
| AC000123.1 | 0.736604727 | 2.18320035 | 1.56748197 | 1.32E-10 |
| AC012186.2 | 0.21806152 | 0.438372981 | 1.00742367 | 6.02E-05 |
| Z82185.1 | 0.625772894 | 0.176676677 | -1.824527568 | 4.09E-33 |
| AC015819.1 | 0.240968812 | 0.505825476 | 1.06979327 | 1.24E-05 |
| AL354863.1 | 0.742019905 | 0.213895468 | -1.794551975 | 1.29E-21 |
| AC009171.2 | 0.62338101 | 1.287885366 | 1.046818074 | 8.38E-08 |
| AL117336.1 | 1.874899555 | 0.766590551 | -1.290285186 | 0.000181952 |
| AC097059.1 | 0.480058222 | 2.480573154 | 2.369392211 | 2.98E-12 |
| AP000696.1 | 4.390311285 | 0.137040063 | -5.001653608 | 2.66E-19 |
| AC009318.4 | 0.322893287 | 0.660254831 | 1.031965508 | 0.014519883 |
| NALT1 | 0.416438753 | 1.442126008 | 1.792020994 | 1.55E-08 |
| AC104984.4 | 3.277198839 | 0.447529095 | -2.872409824 | 8.51E-19 |
| AL049795.1 | 0.139632964 | 0.327150935 | 1.228316824 | 4.48E-05 |
| AC012640.1 | 0.030714886 | 0.373468505 | 3.603976669 | 1.20E-05 |
| SCOC-AS1 | 0.252153071 | 0.697940191 | 1.468803615 | 2.93E-10 |
| AL353801.3 | 0.141102748 | 0.637885554 | 2.176551517 | 2.65E-09 |
| AL080317.1 | 0.211371351 | 1.164813405 | 2.462247107 | 1.30E-15 |
| PTGES2-AS1 | 0.087506163 | 0.259680695 | 1.569282229 | 1.88E-06 |
| VCAN-AS1 | 0.019867613 | 0.252846115 | 3.669769193 | 2.15E-08 |
| PCED1B-AS1 | 0.515660022 | 1.863488193 | 1.853513572 | 1.08E-10 |
| LINC00460 | 0.006752265 | 0.277945182 | 5.363284946 | 3.77E-06 |
| AC116914.2 | 0.368756117 | 1.589397728 | 2.107741299 | 1.04E-10 |
| AC008758.2 | 0.091998069 | 0.258501787 | 1.490498767 | 2.39E-05 |
| RTCA-AS1 | 4.962367011 | 2.054045744 | -1.272560128 | 1.17E-16 |
| HAGLR | 7.078420785 | 3.000089439 | -1.238422016 | 6.22E-13 |
| AC092171.4 | 0.444216926 | 1.777709558 | 2.000683365 | 6.79E-07 |
| AC010997.5 | 0.151590699 | 0.317296377 | 1.065649806 | 0.021083395 |
| LINC00894 | 0.226467424 | 0.703998456 | 1.636268723 | 3.07E-06 |
| Z98200.1 | 0.093768008 | 0.278631939 | 1.571192954 | 9.04E-06 |
| NRAD1 | 1.879483367 | 0.507983323 | -1.887483107 | 4.87E-15 |
| MIR100HG | 0.984992577 | 0.384810336 | -1.355965302 | 4.19E-14 |
| AC005332.3 | 3.602086062 | 9.357643443 | 1.377312607 | 9.74E-11 |
| AC114296.1 | 1.859177605 | 0.913752631 | -1.024789035 | 9.65E-08 |
| DLG1-AS1 | 0.225704728 | 0.452178362 | 1.002455315 | 0.000216727 |
| AP000757.1 | 9.199936184 | 1.36853582 | -2.748990657 | 1.87E-19 |
| AC073130.2 | 0.109936121 | 0.232994391 | 1.083629739 | 0.013413617 |
| PDXDC2P-NPIPB14P | 0.472364681 | 0.962001221 | 1.026137626 | 0.000158751 |
| LHX1-DT | 7.110052574 | 1.314849944 | -2.434962065 | 2.89E-18 |
| CCR5AS | 0.198144133 | 0.735897824 | 1.892955216 | 8.76E-11 |
| AC018816.1 | 0.408535087 | 1.227703708 | 1.587430532 | 6.79E-07 |
| AC069234.4 | 0.119276197 | 0.400105553 | 1.746074488 | 2.33E-06 |
| ZNF710-AS1 | 19.17138884 | 5.23237084 | -1.873418155 | 1.12E-17 |
| PRDM16-DT | 23.63885472 | 0.20540058 | -6.846576076 | 4.42E-20 |
| HOXB-AS3 | 6.437858851 | 1.824811481 | -1.818833518 | 1.87E-14 |
| AL008582.1 | 0.266213689 | 0.81332691 | 1.61125059 | 6.43E-06 |
| AP000695.2 | 0.56585673 | 1.219749553 | 1.108076229 | 1.35E-06 |
| AC068722.2 | 0.101808353 | 0.414321719 | 2.024895513 | 6.59E-07 |
| LINC02154 | 0.01168745 | 1.295822043 | 6.792763612 | 1.03E-06 |
| NARF-IT1 | 0.16668258 | 0.35290727 | 1.082185812 | 0.001283814 |
| AC027307.2 | 5.332815852 | 10.7831869 | 1.015814204 | 7.58E-12 |
| AC107959.3 | 0.327064314 | 1.412008734 | 2.110102753 | 4.98E-13 |
| AL158063.1 | 0.195559027 | 0.531325263 | 1.441991178 | 0.00010842 |
| AC109460.3 | 0.095414744 | 0.265344708 | 1.475583656 | 7.84E-08 |
| LINC01558 | 0.96896086 | 0.423179968 | -1.195167055 | 7.06E-14 |
| AC004034.1 | 0.140860125 | 0.34655596 | 1.298825066 | 4.04E-07 |
| LINC01176 | 0.79394807 | 2.486301252 | 1.646884558 | 3.69E-06 |
| AC099792.1 | 0.03568392 | 1.530579518 | 5.422660081 | 2.32E-11 |
| L3MBTL4-AS1 | 0.049700408 | 0.384967994 | 2.953408914 | 5.70E-14 |
| LINC02343 | 1.577390937 | 0.073425748 | -4.425110387 | 3.77E-30 |
| AL162430.2 | 0.106957323 | 0.224970274 | 1.072699124 | 0.001476926 |
| AL117379.1 | 0.621730025 | 1.618252969 | 1.380076995 | 4.31E-06 |
| AC099850.3 | 0.370365507 | 1.066945981 | 1.526465488 | 2.41E-05 |
| AC092296.4 | 1.097811442 | 0.230222852 | -2.253527334 | 7.49E-10 |
| KLRK1-AS1 | 0.408956418 | 1.03007931 | 1.33273641 | 1.16E-09 |
| Z98884.2 | 0.572338012 | 1.273607395 | 1.153981286 | 1.03E-08 |
| AC073957.3 | 0.687968101 | 1.652541201 | 1.264272664 | 0.000983573 |
| AC005785.1 | 0.256272416 | 0.580776885 | 1.180305832 | 0.000203417 |
| AL358472.3 | 0.857254303 | 2.14290059 | 1.321769779 | 6.84E-10 |
| AC010422.2 | 0.19030083 | 0.414562639 | 1.123308253 | 0.000526556 |
| AC004067.1 | 0.256974801 | 0.821442516 | 1.676532723 | 4.49E-10 |
| AC012065.2 | 0.722302284 | 1.690328901 | 1.226629353 | 0.000858146 |
| AL122125.1 | 0.075366004 | 0.345967129 | 2.19864916 | 2.23E-08 |
| LINC02449 | 0.346825573 | 0.723349101 | 1.060481809 | 2.09E-05 |
| AC096733.2 | 1.992367642 | 0.67957111 | -1.551787457 | 3.98E-16 |
| AC016738.1 | 0.169012916 | 0.441187354 | 1.384257938 | 9.54E-06 |
| AL132712.1 | 0.61705527 | 1.672680754 | 1.438690496 | 0.000833706 |
| AC009283.1 | 3.076581213 | 7.343713009 | 1.255181603 | 5.94E-09 |
| LINC02435 | 0.070724962 | 0.278281455 | 1.976253363 | 0.00026125 |
| APCDD1L-DT | 1.265631491 | 0.101508544 | -3.640184333 | 1.74E-19 |
| AC092375.2 | 0.139218875 | 0.308226991 | 1.146638381 | 0.006009346 |
| AC084036.1 | 2.487542572 | 7.111684009 | 1.515470006 | 1.70E-08 |
| CEP250-AS1 | 0.090789046 | 0.282715877 | 1.638762757 | 2.55E-09 |
| AC083880.1 | 0.746813422 | 1.581107106 | 1.082115339 | 4.20E-07 |
| AC092809.4 | 0.087808479 | 0.376744007 | 2.10115241 | 3.37E-11 |
| AC116407.2 | 0.262450087 | 1.110921567 | 2.081641983 | 3.15E-11 |
| AL031429.2 | 6.50282914 | 0.331106093 | -4.295702053 | 3.02E-26 |
| AL365181.2 | 0.125168382 | 7.730709488 | 5.948658739 | 1.34E-16 |
| AL008635.1 | 0.24892572 | 0.552838011 | 1.151141511 | 0.000149986 |
| EXTL3-AS1 | 0.085884809 | 0.224988996 | 1.389379562 | 0.000312962 |
| NR2F1-AS1 | 1.040844769 | 0.168117276 | -2.63021503 | 3.96E-19 |
| AP001767.2 | 0.254169632 | 0.799297995 | 1.652941801 | 6.29E-05 |
| AL450384.2 | 0.532600176 | 1.356028606 | 1.348262801 | 8.58E-05 |
| U62317.1 | 2.610923444 | 7.269680877 | 1.477331878 | 2.27E-11 |
| LINC01159 | 2.617235698 | 1.067801504 | -1.293400368 | 1.50E-06 |
| AC027796.4 | 0.573092421 | 2.545507583 | 2.151113642 | 2.03E-08 |
| MIR4500HG | 0.084993017 | 0.533749861 | 2.65074757 | 0.011100203 |
| KMT2E-AS1 | 4.306017149 | 9.758249905 | 1.180268365 | 1.41E-05 |
| AP003352.1 | 0.575298101 | 1.377155899 | 1.259310274 | 2.34E-07 |
| AC010655.2 | 0.064385484 | 0.359214404 | 2.480037835 | 2.87E-07 |
| AL023284.4 | 6.607995659 | 18.60695741 | 1.493557522 | 9.15E-08 |
| AL591043.2 | 0.442752398 | 0.221329619 | -1.000303592 | 1.19E-05 |
| AC107081.1 | 0.122302385 | 0.281521829 | 1.202794249 | 0.000147579 |
| AC021491.2 | 0.061648669 | 0.239085581 | 1.955385473 | 4.49E-07 |
| AL135999.3 | 1.688721634 | 0.050004827 | -5.077720354 | 9.33E-23 |
| LINC02724 | 0.100630209 | 0.214740738 | 1.093532445 | 2.41E-05 |
| AC006435.2 | 0.512782481 | 1.304631892 | 1.347223922 | 7.14E-05 |
| LINC00472 | 4.693289242 | 1.302543147 | -1.849268212 | 4.86E-16 |
| AC073316.2 | 0.315827437 | 1.930241185 | 2.611572712 | 2.58E-12 |
| LINC00173 | 0.380962853 | 2.397087179 | 2.653560144 | 6.30E-09 |
| COSMOC | 1.028587383 | 3.030190247 | 1.558744012 | 4.42E-11 |
| AC067852.3 | 0.2409785 | 0.526910442 | 1.128653332 | 0.00249302 |
| LINC02298 | 1.121956196 | 2.356159924 | 1.070421115 | 3.91E-06 |
| LINC02701 | 0.536390694 | 0.17386979 | -1.625276919 | 2.90E-09 |
| AC138150.1 | 0.248683199 | 0.517690639 | 1.057781185 | 0.001504665 |
| AC026333.4 | 0.120153159 | 0.46079743 | 1.939258093 | 6.92E-06 |
| BTG3-AS1 | 0.401002057 | 0.183067239 | -1.131235998 | 1.13E-09 |
| AP001094.2 | 0.103341376 | 0.358767209 | 1.795630039 | 2.12E-07 |
| AC016957.2 | 0.458158778 | 0.934128916 | 1.027774004 | 0.001697541 |
| AL009178.2 | 0.632379362 | 0.278323289 | -1.184028654 | 8.75E-14 |
| KRT7-AS | 0.797050617 | 3.4533189 | 2.115240316 | 0.003413251 |
| HOTAIRM1 | 4.711721816 | 13.66360716 | 1.536012133 | 2.94E-05 |
| AL513218.1 | 0.230292335 | 0.681269016 | 1.5647582 | 2.78E-06 |
| AC107464.2 | 1.211757176 | 5.651972957 | 2.221653937 | 3.66E-09 |
| AC005014.2 | 0.315875639 | 0.914504438 | 1.533633496 | 5.87E-05 |
| AL035665.1 | 0.011531477 | 0.534467867 | 5.534454016 | 1.21E-11 |
| AC046143.2 | 0.585279355 | 1.444650233 | 1.303522945 | 2.15E-07 |
| AC103740.1 | 0.089713446 | 0.2846134 | 1.665607453 | 2.00E-08 |
| AL611929.1 | 0.033265748 | 0.22440754 | 2.754011754 | 4.30E-05 |
| AC093915.1 | 0.049488246 | 0.368156585 | 2.895161698 | 0.000376182 |
| AL121890.5 | 0.118356445 | 0.277063014 | 1.227075866 | 0.006498195 |
| ZNF582-AS1 | 2.406566774 | 0.965659381 | -1.317390155 | 2.65E-15 |
| AC017104.1 | 0.347705605 | 1.291380437 | 1.892975848 | 4.90E-12 |
| AC007384.1 | 0.422880108 | 0.179464861 | -1.236547304 | 1.68E-07 |
| AC104031.1 | 1.08127147 | 0.430610367 | -1.32827382 | 9.13E-13 |
| AC012213.4 | 0.082484474 | 2.686403525 | 5.02540963 | 2.68E-13 |
| LINC01736 | 0.04838963 | 0.866099828 | 4.161763522 | 3.33E-12 |
| AC008875.1 | 0.07206149 | 0.60891714 | 3.07894554 | 1.74E-11 |
| AC024022.1 | 1.156798046 | 0.270455153 | -2.096675729 | 7.87E-17 |
| AL023653.1 | 0.110406069 | 0.376021089 | 1.767994099 | 5.35E-09 |
| C5orf34-AS1 | 0.192237484 | 0.764578055 | 1.991774124 | 8.61E-06 |
| AC002401.4 | 0.418424882 | 5.33153786 | 3.671511182 | 3.58E-11 |
| AC067838.1 | 1.289780393 | 2.626608885 | 1.026075949 | 9.93E-07 |
| AC005699.1 | 0.225924331 | 1.247286364 | 2.464881172 | 2.89E-10 |
| LINC01480 | 0.106730492 | 0.636496158 | 2.576179411 | 9.48E-11 |
| AL731569.1 | 0.223290303 | 0.528458296 | 1.242869021 | 1.16E-06 |
| AC098851.1 | 0.288487645 | 0.823294251 | 1.512898616 | 0.000138312 |
| AC136475.2 | 2.097232779 | 4.248046292 | 1.018312488 | 2.57E-05 |
| PCAT19 | 1.215531172 | 0.344798234 | -1.817762601 | 1.10E-16 |
| AC126118.1 | 0.204141433 | 1.086585356 | 2.412160578 | 1.92E-08 |
| LYPLAL1-DT | 0.963831045 | 0.383734682 | -1.328671111 | 3.75E-09 |
| HOXB-AS1 | 1.024601848 | 2.378253052 | 1.214838831 | 3.73E-06 |
| AC015917.2 | 0.253458781 | 0.548276482 | 1.113152446 | 0.000274279 |
| KIF1C-AS1 | 0.234461331 | 0.548405313 | 1.225892546 | 1.23E-07 |
| RNF32-AS1 | 0.123456622 | 0.302837045 | 1.294537478 | 0.001828272 |
| AC010998.2 | 0.096095384 | 0.221679171 | 1.205934178 | 0.00103459 |
| LINP1 | 0.205285897 | 3.144160316 | 3.936968354 | 6.81E-16 |
| AC004069.1 | 0.20272308 | 0.555228377 | 1.453570956 | 2.15E-06 |
| AL512274.1 | 0.75259669 | 2.16609225 | 1.525145838 | 0.000179066 |
| AC104063.1 | 0.035024295 | 0.419038461 | 3.580654762 | 2.11E-11 |
| AC006449.5 | 0.54795315 | 1.326907653 | 1.275943516 | 9.91E-09 |
| AL121992.3 | 3.035867895 | 0.660336306 | -2.20083614 | 1.05E-10 |
| AC066613.1 | 0.221560359 | 0.498307204 | 1.169335648 | 2.23E-05 |
| PICART1 | 0.369243698 | 0.986732695 | 1.418086013 | 6.74E-06 |
| AL591895.1 | 14.27808367 | 32.89455375 | 1.20404638 | 2.08E-07 |
| AC067930.4 | 0.06461851 | 0.280871866 | 2.119892738 | 8.19E-06 |
| AC107057.1 | 4.0369135 | 0.085464361 | -5.561785934 | 8.42E-24 |
| AC009133.1 | 0.970336951 | 2.721948463 | 1.488082034 | 1.49E-16 |
| ITGB1-DT | 0.068438854 | 0.354603692 | 2.373320049 | 3.34E-08 |
| AC131009.3 | 0.574674247 | 1.209711808 | 1.073847088 | 1.46E-06 |
| AP006623.1 | 0.374988696 | 0.801444719 | 1.095755904 | 2.70E-06 |
| AL731577.2 | 0.63088882 | 0.304230095 | -1.052222913 | 3.76E-10 |
| U47924.3 | 0.203895404 | 1.061305697 | 2.379939108 | 6.98E-11 |
| AL590617.2 | 0.890506519 | 1.789352524 | 1.006739565 | 3.91E-06 |
| GARS1-DT | 0.462760855 | 1.273826619 | 1.460830187 | 1.43E-08 |
| LINC00342 | 1.08991465 | 3.47921971 | 1.674548623 | 8.78E-07 |
| AC009509.4 | 0.735454699 | 2.238729381 | 1.605971759 | 6.54E-09 |
| AL355803.1 | 1.268610221 | 0.275657259 | -2.202301374 | 5.51E-17 |
| AL513320.1 | 0.613792643 | 1.49499644 | 1.284318791 | 0.000548708 |
| TENM3-AS1 | 0.126925601 | 0.715637466 | 2.495245827 | 4.60E-10 |
| AL031705.1 | 0.125169762 | 0.348305993 | 1.476469219 | 4.99E-06 |
| DGCR5 | 0.318694578 | 2.443276221 | 2.938570595 | 4.17E-13 |
| AC108134.2 | 0.324725464 | 1.279194506 | 1.97794322 | 2.05E-12 |
| AC073912.2 | 0.093184854 | 0.256102899 | 1.4585562 | 0.003856358 |
| TCL6 | 1.6011905 | 0.072260177 | -4.469800366 | 1.47E-18 |
| HOXC-AS2 | 1.68377982 | 3.623608478 | 1.105723588 | 2.81E-06 |
| AL645608.8 | 0.160185655 | 0.567188462 | 1.82408323 | 1.66E-10 |
| TDRKH-AS1 | 0.247374403 | 0.533118571 | 1.107760214 | 5.56E-11 |
| AC010973.2 | 0.364378175 | 1.324715393 | 1.862173984 | 6.34E-10 |
| LNCSRLR | 0.153971482 | 0.382500881 | 1.312799905 | 0.00055641 |
| AC025171.5 | 0.180024261 | 0.580347654 | 1.688726049 | 1.08E-06 |
| SOX9-AS1 | 0.352729125 | 0.740135052 | 1.069227837 | 4.87E-05 |
| LINC00900 | 0.071697469 | 0.323022211 | 2.171639262 | 6.89E-13 |
| AC006017.1 | 0.29843733 | 1.491174341 | 2.320949027 | 4.65E-06 |
| AC009549.1 | 0.254052637 | 2.230004205 | 3.133847087 | 2.84E-13 |
| AL162724.2 | 0.232292848 | 1.121815179 | 2.271818369 | 1.58E-11 |
| AC004846.2 | 0.324957953 | 1.051996742 | 1.694805273 | 4.10E-06 |
| TRG-AS1 | 0.126910091 | 0.278594084 | 1.134357833 | 1.11E-05 |
| DARS1-AS1 | 0.107988399 | 0.235532646 | 1.125050704 | 1.65E-06 |
| AC108488.1 | 1.351043685 | 2.955056153 | 1.129111221 | 7.38E-10 |
| CRNDE | 2.387187762 | 12.1247097 | 2.344566255 | 4.07E-12 |
| AC020659.1 | 0.916130855 | 6.550888902 | 2.838065097 | 3.16E-09 |
| AC005306.1 | 0.170593771 | 0.348345029 | 1.029952008 | 0.005757508 |
| SERTAD4-AS1 | 3.724718401 | 11.12135321 | 1.578129078 | 2.45E-06 |
| AC048341.2 | 1.459142365 | 3.272338175 | 1.165201199 | 6.56E-05 |
| NSMCE1-DT | 0.098643456 | 0.270512214 | 1.455398483 | 6.06E-08 |
| AC011472.2 | 0.128079115 | 0.493093445 | 1.944825825 | 4.48E-11 |
| LINC00501 | 0.360844419 | 0.790763652 | 1.131869618 | 1.80E-05 |
| LINC01508 | 1.385692069 | 3.494439102 | 1.33445421 | 2.70E-06 |
| AC008735.2 | 0.951357137 | 2.194742735 | 1.205992907 | 5.82E-05 |
| AL157904.1 | 0.201369332 | 0.709375835 | 1.816706202 | 4.20E-07 |
| AL133383.1 | 0.510715678 | 0.208165128 | -1.294791942 | 2.78E-10 |
| AL158196.1 | 0.123972066 | 0.293998512 | 1.24579377 | 0.006830025 |
| CCND2-AS1 | 0.082802591 | 0.51687115 | 2.642056868 | 1.99E-08 |
| AP000907.2 | 0.106508225 | 0.268212107 | 1.332409518 | 0.005428164 |
| PRKAR1B-AS1 | 0.618491698 | 2.088129498 | 1.755385051 | 2.57E-05 |
| AL110115.2 | 0.105492837 | 0.241869859 | 1.197085951 | 0.012882586 |
| AC087239.1 | 0.26865037 | 1.117995234 | 2.057112311 | 2.72E-07 |
| AF186192.1 | 1.053735308 | 0.265085729 | -1.990981605 | 7.01E-15 |
| DTX2P1-UPK3BP1-PMS2P11 | 0.149914465 | 0.318574701 | 1.087492109 | 2.40E-06 |
| AL139351.1 | 0.09042766 | 1.196783038 | 3.726253697 | 2.57E-09 |
| AC069549.1 | 0.083267803 | 0.25431238 | 1.610771027 | 0.002826375 |
| AL391121.1 | 4.504825718 | 1.78338724 | -1.336851293 | 1.48E-14 |
| AC100803.3 | 0.602215586 | 0.206666342 | -1.542976602 | 1.53E-13 |
| AC008105.1 | 0.136727339 | 0.289821161 | 1.083861193 | 0.001531138 |
| AC012593.2 | 2.269055694 | 11.56531897 | 2.34964113 | 2.50E-06 |
| AC008105.2 | 0.121249161 | 0.609367659 | 2.329338174 | 2.94E-11 |
| AC124312.2 | 2.00572144 | 0.697310482 | -1.524248181 | 1.59E-12 |
| AC127024.6 | 0.312194161 | 0.822208491 | 1.397060715 | 7.08E-05 |
| AC103706.1 | 0.336315886 | 1.058142163 | 1.653644635 | 1.24E-11 |
| CD27-AS1 | 4.167596911 | 10.33430049 | 1.310153087 | 1.81E-14 |
| LINC02313 | 0.197168203 | 1.798116705 | 3.188987849 | 5.26E-11 |
| AC103591.3 | 0.254295317 | 0.729623376 | 1.520647056 | 0.000112126 |
| AC124319.1 | 0.115638517 | 0.367952727 | 1.669898419 | 0.002197667 |
| AC244197.2 | 0.385445189 | 0.859619896 | 1.157173154 | 0.004364472 |
| HOXA10-AS | 0.981672066 | 2.052740395 | 1.064238116 | 1.97E-06 |
| AC013468.1 | 0.251962908 | 0.523595393 | 1.055241037 | 0.001744584 |
| SLC12A9-AS1 | 0.373959723 | 0.758280043 | 1.019847861 | 9.23E-06 |
| MRPL20-DT | 0.655392415 | 1.462997675 | 1.158496596 | 3.12E-07 |
| PGM5-AS1 | 0.758885675 | 0.208936144 | -1.860820474 | 8.73E-14 |
| LINC01116 | 8.501959775 | 3.679392146 | -1.208327988 | 4.13E-11 |
| AL121772.1 | 0.071049107 | 0.349342901 | 2.297755406 | 4.95E-08 |
| U91328.3 | 0.232295938 | 0.682504728 | 1.554875115 | 1.43E-06 |
| AC069209.1 | 0.069643493 | 0.305634896 | 2.133748809 | 5.04E-09 |
| AC079760.2 | 0.122730433 | 1.335308817 | 3.443608492 | 3.25E-08 |
| HOTAIR | 0.221387066 | 0.952890923 | 2.105740144 | 3.62E-07 |
| AC073218.1 | 0.086009372 | 0.49102121 | 2.513219562 | 0.000397087 |
| AL132780.1 | 1.419822312 | 0.403804149 | -1.813982751 | 2.12E-14 |
| AC040934.1 | 0.06593065 | 0.239021319 | 1.858118095 | 3.97E-06 |
| AL033397.2 | 0.067849488 | 0.220984458 | 1.703535076 | 1.30E-05 |
| AC068790.7 | 0.113609129 | 0.286505174 | 1.334482428 | 5.83E-05 |
| AC148477.4 | 6.448800906 | 0.524333344 | -3.620474732 | 4.25E-19 |
| LINC02432 | 1.211087019 | 0.185611255 | -2.705946429 | 2.96E-15 |
| SATB2-AS1 | 0.157323307 | 0.826505373 | 2.393291784 | 7.19E-10 |
| SLC9A3-AS1 | 3.584379939 | 12.02590855 | 1.746350418 | 3.85E-05 |
| LINC00462 | 0.063878556 | 1.396629685 | 4.450474032 | 3.57E-05 |
| AC003102.1 | 0.790467397 | 1.793116342 | 1.181691232 | 1.72E-05 |
| PRKAR1B-AS2 | 5.50825291 | 24.28421869 | 2.140352363 | 2.27E-05 |
| AC005034.4 | 0.682127735 | 0.302594656 | -1.172655413 | 3.24E-09 |
| AP001094.1 | 0.226951884 | 0.546427229 | 1.267642914 | 0.000414009 |
| AC060766.3 | 1.107758988 | 2.522341796 | 1.187119753 | 9.45E-06 |
| PARD3-AS1 | 0.720675049 | 4.081300633 | 2.501608182 | 1.92E-13 |
| AC005062.1 | 0.114750929 | 0.246882703 | 1.105319925 | 0.001805148 |
| VSTM2A-OT1 | 0.006531865 | 0.302940004 | 5.53539324 | 5.19E-06 |
| AC124016.1 | 0.670500789 | 1.357799026 | 1.01795902 | 0.001971837 |
| AL645940.1 | 0.170378129 | 0.448742919 | 1.397149019 | 0.000167895 |
| AL353807.2 | 0.042758567 | 0.22603443 | 2.402257126 | 2.80E-06 |
| AF124730.1 | 0.097964578 | 0.487256821 | 2.314350282 | 8.74E-08 |
| AC005046.1 | 2.027126991 | 6.393137336 | 1.657087615 | 0.000451539 |
| LINC02029 | 0.0153133 | 0.230585181 | 3.912442648 | 2.50E-14 |
| AC107021.1 | 0.145351573 | 0.340898404 | 1.229795165 | 0.02884962 |
| LINC00845 | 0.584076454 | 0.16641035 | -1.811412062 | 3.92E-07 |
| AC027682.4 | 0.087116644 | 0.257467836 | 1.563371936 | 1.13E-05 |
| AC068790.4 | 0.124128063 | 0.258449854 | 1.058055073 | 0.00190528 |
| AL133371.2 | 0.476424992 | 1.045596362 | 1.134005026 | 0.000176194 |
| LINC01752 | 0.492470396 | 0.209838415 | -1.230758186 | 4.88E-08 |
| LINC00893 | 0.434289024 | 0.941974368 | 1.11703231 | 0.000364997 |
| FAM222A-AS1 | 0.548888919 | 0.256460579 | -1.097777131 | 1.86E-09 |
| CYP4F26P | 0.027051056 | 0.258616523 | 3.257057612 | 5.48E-07 |
| LINC00839 | 5.888777853 | 1.225204893 | -2.264945217 | 1.96E-13 |
| AL136366.1 | 0.127258375 | 1.962658606 | 3.94697674 | 1.96E-09 |
| AC027348.1 | 0.163089063 | 0.574109858 | 1.815666797 | 0.003727737 |
| Z98257.1 | 0.01067711 | 0.456992065 | 5.419576057 | 3.39E-14 |
| AC002091.2 | 0.153222904 | 0.399883916 | 1.383949288 | 0.002386418 |
| LINC01055 | 10.52023388 | 0.230895569 | -5.509782484 | 8.03E-17 |
| AC008655.2 | 0.203056411 | 0.478349524 | 1.23618459 | 0.000306456 |
| AC096921.2 | 0.749471348 | 0.362475598 | -1.047989448 | 4.99E-11 |
| AC073842.2 | 0.347370173 | 0.836708397 | 1.268251031 | 7.69E-06 |
| LINC01871 | 0.494364781 | 1.519787372 | 1.620221623 | 3.06E-07 |
| ARHGAP31-AS1 | 0.456346991 | 1.219326152 | 1.417880954 | 1.42E-07 |
| LINC02586 | 1.027359808 | 0.174646398 | -2.556432747 | 8.77E-18 |
| LINC01127 | 0.561225843 | 0.168203124 | -1.738376942 | 3.55E-11 |
| AP000424.2 | 1.211205562 | 0.257500763 | -2.233795124 | 1.68E-13 |
| AC010201.2 | 0.55530342 | 1.247239478 | 1.167390313 | 4.20E-05 |
| AC010624.2 | 0.095116258 | 0.29880434 | 1.651437237 | 0.023466172 |
| AL590560.3 | 0.908919357 | 2.319264768 | 1.351443326 | 0.002025879 |
| FBXL19-AS1 | 0.533058358 | 1.256067591 | 1.236548711 | 5.46E-09 |
| AC018904.1 | 2.534833644 | 8.615131246 | 1.764981706 | 2.71E-10 |
| AC108134.4 | 0.816178965 | 2.156948932 | 1.402034585 | 7.76E-10 |
| AC114956.1 | 0.160136611 | 0.751280178 | 2.230047854 | 5.34E-05 |
| KCNIP2-AS1 | 0.23438996 | 0.538095594 | 1.198951719 | 7.19E-09 |
| AC253576.2 | 0.248427691 | 0.552301391 | 1.15262977 | 0.000132832 |
| AC010487.1 | 0.376452993 | 1.712993267 | 2.185977846 | 1.62E-05 |
| AL356740.1 | 0.967828241 | 0.146242856 | -2.726384886 | 8.62E-19 |
| AL133410.1 | 0.392769703 | 0.944367595 | 1.26566489 | 0.000137235 |
| AC009812.4 | 0.75469088 | 1.744189246 | 1.208598837 | 1.45E-08 |
| AL078587.1 | 0.101554639 | 0.278186746 | 1.453797541 | 0.000166528 |
| AC107982.3 | 0.284572513 | 0.636039348 | 1.160319701 | 2.01E-07 |
| AC005393.1 | 0.290881417 | 0.668495484 | 1.200486683 | 8.65E-05 |
| PVT1 | 0.137610934 | 1.060098693 | 2.945531574 | 6.28E-17 |
| AC004908.1 | 0.623783274 | 1.335480981 | 1.098242657 | 0.000327794 |
| AC006273.1 | 0.950861088 | 0.288092266 | -1.722703661 | 8.58E-13 |
| AC103702.2 | 1.462964777 | 4.193218531 | 1.519162984 | 3.04E-06 |
| AC254629.1 | 0.011219401 | 0.310605395 | 4.791015387 | 9.32E-12 |
| AC073655.2 | 0.214216759 | 0.500361447 | 1.22389928 | 6.61E-05 |
| AC105020.5 | 0.405183035 | 0.823220924 | 1.022705883 | 0.000197053 |
| AL844908.2 | 0.474650894 | 0.958920739 | 1.014544772 | 0.021362783 |
| GUSBP11 | 0.276116335 | 0.711192062 | 1.364962983 | 0.005057233 |
| LINC01230 | 2.344935159 | 0.14085208 | -4.057295262 | 1.62E-28 |
| AC009120.2 | 1.010139334 | 2.971657477 | 1.55671353 | 8.87E-07 |
| TRBV11-2 | 0.143501462 | 0.300679329 | 1.067160254 | 0.024738229 |
| AC092123.1 | 0.313504773 | 0.674571144 | 1.105483198 | 2.04E-05 |
| AP001178.1 | 0.134081824 | 0.307284269 | 1.196460238 | 0.001035497 |
| AC020779.2 | 1.392787791 | 0.546392892 | -1.349964841 | 1.79E-07 |
| AC020558.2 | 0.434450929 | 1.082698354 | 1.317366216 | 0.000259694 |
| SLBP-DT | 0.308624361 | 0.912886826 | 1.564584071 | 7.07E-06 |
| AL132655.2 | 0.069660637 | 0.264181076 | 1.923111565 | 0.001540773 |
| AC123595.1 | 0.690429284 | 0.208822709 | -1.725215048 | 8.06E-13 |
| NFE2L1-DT | 0.194127678 | 0.479322142 | 1.303989759 | 5.29E-07 |
| AC006033.2 | 0.075590105 | 0.236208925 | 1.643794176 | 1.88E-07 |
| AL139123.1 | 0.143475054 | 0.612554385 | 2.094038022 | 5.63E-08 |
| C10orf55 | 1.049289127 | 0.22189014 | -2.241494798 | 2.23E-10 |
| TMEM252-DT | 0.612261647 | 2.654201133 | 2.116057483 | 4.38E-07 |
| AC020913.3 | 0.099706786 | 0.244734532 | 1.295454077 | 0.026173312 |
| AC087477.5 | 0.19973398 | 0.848137951 | 2.086219149 | 2.20E-05 |
| AC026369.3 | 0.024213573 | 0.760280422 | 4.972643778 | 7.04E-12 |
| AC016737.2 | 0.172311019 | 0.366797275 | 1.089967957 | 0.003956536 |
| AL359921.1 | 0.273913933 | 0.614861018 | 1.166537689 | 3.52E-06 |
| LINC02604 | 1.782503066 | 5.617821223 | 1.656106156 | 1.91E-07 |
| AC138932.4 | 0.258095998 | 0.56427922 | 1.128501449 | 0.001673519 |
| AC136475.1 | 0.501254644 | 1.327191108 | 1.404760522 | 8.94E-06 |
| AC104695.2 | 1.298486198 | 2.982969776 | 1.199918681 | 0.004188723 |
| AC002064.2 | 0.345744674 | 1.375498666 | 1.992175806 | 8.75E-05 |
| AC135178.6 | 0.415279172 | 1.530788107 | 1.882121177 | 1.13E-13 |
| CR936218.1 | 0.375723777 | 0.844031651 | 1.167624685 | 0.000700876 |
| IBA57-DT | 0.241035381 | 0.500368521 | 1.053746101 | 0.003198798 |
| AC099791.2 | 0.121307421 | 0.459351237 | 1.920929899 | 1.97E-05 |
| AL451050.2 | 0.16638295 | 0.452235865 | 1.442567807 | 2.44E-05 |
| LINC01315 | 1.486395959 | 5.039043725 | 1.761331491 | 1.65E-11 |
| AC096642.1 | 0.110195997 | 0.233461961 | 1.083115687 | 0.002255664 |
| LINC00654 | 0.276162026 | 0.957323586 | 1.793491703 | 1.16E-06 |
| LINC01426 | 0.304153052 | 1.265638609 | 2.056996132 | 0.000238234 |
| AL158151.4 | 0.046782599 | 0.536111486 | 3.518489133 | 9.41E-12 |
| AC102953.2 | 0.900395236 | 1.842066009 | 1.032694432 | 7.51E-05 |
| AC004596.1 | 0.18236711 | 0.396725899 | 1.121297018 | 1.71E-06 |
| AL157392.4 | 0.250588887 | 0.541192602 | 1.110819684 | 0.0084393 |
| CACNA1C-AS2 | 0.148333793 | 0.335653921 | 1.178127184 | 0.000274084 |
| AC018755.4 | 0.356381241 | 1.12143059 | 1.653847026 | 3.12E-05 |
| AC092954.1 | 0.055617786 | 0.397812699 | 2.838471099 | 4.76E-05 |
| LINC00997 | 1.350496233 | 3.306043266 | 1.291615989 | 8.92E-10 |
| AL161452.1 | 0.102034925 | 0.285166586 | 1.482741896 | 6.72E-07 |
| LINC02188 | 0.235795294 | 1.512397176 | 2.681230231 | 1.96E-08 |
| SEMA6A-AS1 | 0.207099628 | 0.489578878 | 1.241216351 | 0.00051691 |
| AC027601.1 | 0.149833755 | 0.380388211 | 1.344109857 | 3.16E-07 |
| AC011462.4 | 1.184579836 | 2.46098707 | 1.054861643 | 0.000102175 |
| AC137630.3 | 0.355075025 | 0.765589704 | 1.108447538 | 0.02888181 |
| AL022344.1 | 0.28698996 | 0.823989925 | 1.52162643 | 9.72E-06 |
| AC004492.1 | 0.386314312 | 0.787135871 | 1.026837562 | 1.91E-05 |
| AC093010.2 | 23.87997926 | 51.10471472 | 1.097654811 | 4.60E-10 |
| AC084782.3 | 0.449079481 | 1.220511198 | 1.442442822 | 4.97E-06 |
| AL022322.1 | 0.350039584 | 1.268913021 | 1.8580032 | 2.20E-08 |
| SBNO1-AS1 | 0.493146836 | 1.200541562 | 1.283596168 | 1.68E-06 |
| ATP2C2-AS1 | 0.085597176 | 0.22177017 | 1.373430228 | 0.000325276 |
| AL162582.1 | 0.958761248 | 2.21726163 | 1.20953551 | 0.018109906 |
| LAMC1-AS1 | 0.218222275 | 0.614273622 | 1.493083062 | 0.000158415 |
| AC022509.3 | 0.489324253 | 2.008935278 | 2.03756839 | 5.56E-11 |

# Appendix 5

**111 different lncRNAs**

**Table 5a. 111 different lncRNAs.**

| id | TCGA-B9-A5W7 | TCGA-2Z-A9J8 | TCGA-BQ-5879 | TCGA-P4-A5EA | TCGA-MH-A857 |
| --- | --- | --- | --- | --- | --- |
| futime | 1.602739726 | 7.643835616 | 1.909589041 | 0.550684932 | 2.438356164 |
| fustat | 0 | 0 | 1 | 1 | 0 |
| LUCAT1 | 0.132264528 | 1.125024 | 2.453698 | 4.964465491 | 0.013156739 |
| LINC01559 | 0.77732596 | 0.03412068 | 7.859183 | 4.659030418 | 0.00966536 |
| FOXD2-AS1 | 0.848939005 | 1.815829 | 1.781243 | 5.55967941 | 0.083754258 |
| AL157935.1 | 0.062353278 | 0.08920572 | 0.08595888 | 0.360780976 | 1.607126984 |
| KLHL6-AS1 | 0.034709578 | 2.443137 | 0.09066292 | 0.154927815 | 0.037979294 |
| AC092115.3 | 0.17694849 | 0.9113449 | 0.3466479 | 0.219393837 | 0.258156553 |
| AC007342.4 | 1.120780433 | 1.693779 | 0.02061639 | 0.046973292 | 0.379998977 |
| LRRK2-DT | 9.946988652 | 31.20228 | 2.930222 | 2.312795938 | 0.938421715 |
| CD44-AS1 | 0.267683577 | 0.0765923 | 0.1359558 | 3.141929041 | 0.032544438 |
| AC087623.1 | 0.359640627 | 0.1683883 | 0.320249 | 1.507983404 | 0.178872518 |
| LINC02471 | 45.43525577 | 21.12563 | 4.326833 | 6.22021176 | 0.460327347 |
| AC141002.1 | 0.31288383 | 0.590868 | 0.1225899 | 1.76899059 | 0.479301414 |
| ADAMTS9-AS1 | 48.84929304 | 20.16899 | 3.371532 | 0.530813742 | 0.15669943 |
| LINC01117 | 0.299723909 | 3.04448 | 1.565784 | 1.189183645 | 3.197595757 |
| AC124798.1 | 4.650866741 | 3.311286 | 0.138408 | 0.621699579 | 9.005123792 |
| MAFG-DT | 1.225348843 | 2.404176 | 2.226026 | 4.564688481 | 0.786321701 |
| AC113139.1 | 0.219149595 | 0.5643475 | 0.09540471 | 2.173743117 | 0.31972527 |
| AC156455.1 | 0.600716448 | 12.5229 | 1.849293 | 3.404858142 | 0.052167081 |
| LINC00857 | 3.313253848 | 6.702879 | 0.3203375 | 0.765766345 | 0.026398321 |
| LNCTAM34A | 1.134386532 | 1.475834 | 0.1041703 | 1.107615909 | 1.086092137 |
| AC130371.2 | 4.74333935 | 0.6131981 | 0.4664837 | 2.46582873 | 3.75193157 |
| ANKRD44-AS1 | 0.268598734 | 0.8893124 | 0.02505684 | 3.025802644 | 0.041985901 |
| CASC19 | 0.190287932 | 0.8167063 | 0.6523654 | 5.025374243 | 0.052053365 |
| AC008514.1 | 0.043977123 | 0.06291587 | 0.6413584 | 1.112332277 | 0.064159815 |
| TMEM51-AS1 | 0.627045637 | 1.281245 | 0.1316719 | 0.713431676 | 1.592862291 |
| AC136475.5 | 0.314451925 | 1.185311 | 0.09820633 | 0.040683211 | 0 |
| AL365181.3 | 10.68017593 | 30.32662 | 11.77948 | 6.554523646 | 0.017787323 |
| AC090197.1 | 0.116441302 | 0.05934653 | 0.1877174 | 0.249042641 | 0.03185258 |
| AL590666.2 | 6.172809961 | 12.25814 | 13.02526 | 0.411233196 | 0.025202619 |
| AP001189.3 | 0.160911684 | 0.09208332 | 2.39926 | 0.23941226 | 0.293449845 |
| AC012640.1 | 0.050752668 | 0.1524795 | 0 | 0.075512297 | 0.083300516 |
| SCOC-AS1 | 1.115632087 | 1.050925 | 0.09934362 | 0.44191927 | 0.134755578 |
| DLG1-AS1 | 0.278270826 | 0.7608289 | 0.08749186 | 0.322019383 | 0.372147758 |
| ZNF710-AS1 | 3.260762718 | 3.325756 | 1.932882 | 3.213461977 | 33.86245375 |
| AP000695.2 | 0.726219838 | 1.730197 | 3.058296 | 3.682538583 | 0.551376814 |
| LINC02154 | 0.011213178 | 0.02887584 | 2.921608 | 0.784125126 | 0 |
| AC107959.3 | 1.605189939 | 1.411901 | 1.785833 | 0.265364441 | 0.281891718 |
| AC099850.3 | 0.761448125 | 0.7936808 | 4.794755 | 0.485537014 | 0.178538177 |
| KLRK1-AS1 | 0.931553561 | 1.239171 | 0.906429 | 0.413049597 | 0.168759584 |
| AC012065.2 | 0.914704118 | 0.7851729 | 0.1038803 | 0.769227775 | 0.261096832 |
| LINC02435 | 0.119581628 | 1.180448 | 0.0780881 | 0.578238002 | 0 |
| NR2F1-AS1 | 0.029399732 | 0.05047287 | 0.1713083 | 0.487895516 | 0.168269919 |
| AL591043.2 | 0.060307142 | 0.3364858 | 0.01969061 | 0 | 0.131976407 |
| AL513218.1 | 0.714431416 | 0.4216168 | 0.05831643 | 1.926625772 | 0.097716547 |
| AC107464.2 | 4.477252985 | 3.883898 | 0.06187714 | 0.916393469 | 0.025920739 |
| AL035665.1 | 0.198753407 | 0 | 0.04781668 | 0.295715024 | 0.022892253 |
| AL611929.1 | 0.583260058 | 0.4339093 | 0 | 0 | 0 |
| AC024022.1 | 0.157999255 | 0.135625 | 0 | 0 | 1.426285751 |
| AC136475.2 | 4.409267487 | 2.98202 | 1.163353 | 1.656647337 | 0.19493454 |
| ITGB1-DT | 0.17561359 | 0.7286006 | 8.658157 | 4.44187005 | 0.032026127 |
| AC009509.4 | 1.749321815 | 2.119906 | 0.02239855 | 1.684117088 | 0.337784409 |
| DARS1-AS1 | 0.215378059 | 0.2761763 | 0.1771075 | 0.530127148 | 0.058189415 |
| SERTAD4-AS1 | 23.47563639 | 5.627424 | 1.737382 | 4.062020068 | 2.416489326 |
| LINC01508 | 5.170988156 | 16.2722 | 4.547495 | 7.774976867 | 4.354229748 |
| AC040934.1 | 0.034032978 | 0.2921357 | 0.08889562 | 0.151907779 | 0.148955828 |
| AL033397.2 | 0.127670331 | 0 | 0.4376931 | 0.522373917 | 0.174621434 |
| LINC00462 | 0.508159767 | 0.04673564 | 7.54922 | 1.6201391 | 0 |
| AC005034.4 | 0.116522254 | 0.9668749 | 0.06340858 | 0.173367499 | 0.276247613 |
| AP001094.1 | 0.180921427 | 0.1553011 | 0.2953591 | 0 | 0.395929221 |
| AL353807.2 | 0.079358717 | 0.2043622 | 0.1554663 | 0 | 0 |
| LINC00839 | 0 | 0.02565094 | 0.02927051 | 3.801399548 | 2.297007856 |
| AP000424.2 | 0 | 0.3216 | 0.3425152 | 1.226345634 | 0.327958539 |
| AC010624.2 | 0 | 0 | 0 | 2.303978682 | 0.066447252 |
| FBXL19-AS1 | 0.547938699 | 1.168277 | 0.3924367 | 1.262322502 | 0.754279738 |
| AC138932.4 | 0.403136644 | 0.3992867 | 0.2632526 | 1.891697051 | 0.169658836 |
| AC096642.1 | 0.093506063 | 0.2522604 | 0.03925316 | 0.129185537 | 0.124239076 |
| CACNA1C-AS2 | 0.688036167 | 0.04921695 | 0.05616188 | 0 | 0.062737553 |
| AL022344.1 | 1.082164322 | 0.1703018 | 0.03533325 | 0 | 0.335496842 |
| AC074212.1 | 0.483477722 | 0.7954405 | 0.2280173 | 0.759305192 | 0.205730914 |
| AC090152.1 | 0.070116135 | 5.59739 | 0.06540942 | 1.341285555 | 0.076721225 |
| SNHG4 | 0.253816042 | 0.9882104 | 0.2190197 | 0.202307089 | 0.406670291 |
| RAMP2-AS1 | 0.041887998 | 0.2049506 | 0.04376531 | 0.081019912 | 0.105418055 |
| MIR4435-2HG | 1.314553805 | 3.26707 | 3.080915 | 8.138200134 | 2.074424573 |
| AC107021.2 | 1.31671074 | 0.5436652 | 5.888178 | 0.644756073 | 0.082067907 |
| AC127537.1 | 1.7249131 | 0.7203153 | 0.1522145 | 1.664698333 | 0.994712699 |
| DUSP5-DT | 0.027800824 | 1.097742 | 1.143717 | 8.396775063 | 0.030419721 |
| AL355102.4 | 0 | 0.4061398 | 0.7878645 | 0 | 4.115813009 |
| AC023906.5 | 0.357426662 | 0.4248159 | 0.08977057 | 0 | 0.120337543 |
| AC109460.1 | 0.259875636 | 0.3244722 | 0.1311331 | 0.456956689 | 0.232655297 |
| AC010615.2 | 0.044105311 | 0 | 0.01772384 | 0.005047849 | 0.259862212 |
| AC130343.2 | 0.24590025 | 0.9850321 | 0.2408633 | 0.426839464 | 0.134532288 |
| AL391422.4 | 6.487812237 | 5.28682 | 0.3757619 | 0.602129205 | 1.466845346 |
| AC022382.1 | 0.415688517 | 0.1338086 | 0.1017934 | 0.541171463 | 0 |
| AL158166.1 | 0.094118155 | 0.3231603 | 0.09219026 | 2.800671724 | 0 |
| AC008622.2 | 0.894964863 | 1.195023 | 0 | 0.105680351 | 0.264248123 |
| AC026356.2 | 0.126373012 | 0.183577 | 1.364803 | 1.388484467 | 0.138277619 |
| SCGB1B2P | 1.077011158 | 1.411072 | 0.8143473 | 2.361475477 | 1.550615658 |
| LINC02709 | 0.225533132 | 2.135243 | 1.633026 | 0.207257156 | 0.159680421 |
| AC017083.1 | 0.089167098 | 0.8572496 | 0.1280996 | 1.565472507 | 0.253673753 |
| LINC02535 | 0.159791129 | 2.373518 | 10.82016 | 2.832267046 | 0 |
| LINC01637 | 1.327286143 | 0.8325867 | 0.5333737 | 0.417746393 | 2.625346464 |
| AL162171.1 | 0.656533118 | 1.043632 | 0.3175728 | 0.180892968 | 1.037659905 |
| ADORA2A-AS1 | 0.203868108 | 1.169138 | 0.03115764 | 0.019361165 | 0.04271609 |
| LINC01914 | 0.077768008 | 1.535371 | 0.4189627 | 0.144633799 | 0.06382044 |
| MNX1-AS2 | 0.093363196 | 1.202131 | 0.1829015 | 0.138910322 | 0.051079104 |
| LINC02561 | 0.126148249 | 5.933985 | 0.8072865 | 0.037537905 | 0.055212673 |
| LBX2-AS1 | 13.92357066 | 28.66431 | 0.6127143 | 1.956942489 | 0.256670047 |
| CYTOR | 1.515531053 | 3.720622 | 4.159863 | 18.21943927 | 0.591459371 |
| SLC25A5-AS1 | 0.786078514 | 0.743216 | 0.09671205 | 0.220353013 | 2.356004925 |
| AL049555.1 | 0.334185182 | 0.2170842 | 6.717839 | 6.651957563 | 3.795021524 |
| MNX1-AS1 | 0.016771295 | 0.3887004 | 1.270413 | 1.472235912 | 0 |
| AL031710.1 | 2.797903482 | 0.3522482 | 0 | 0.055504765 | 57.88222949 |
| ZFAS1 | 25.07919434 | 25.33617 | 40.97804 | 43.24353265 | 9.530500006 |
| AL353751.1 | 10.28212941 | 5.879115 | 4.596395 | 4.363595989 | 1.415779514 |
| LINC01730 | 0.1035523 | 0.1777765 | 0.01352415 | 0.986049072 | 0.113307147 |
| AC104564.1 | 0.258650633 | 0.499552 | 0.1689017 | 0.481041301 | 0.283016073 |
| MRPS9-AS1 | 0.286681736 | 0.2706932 | 4.399348 | 1.194309436 | 0.439162871 |
| AC007342.5 | 1.092201202 | 0.916545 | 0.01064506 | 0.036381275 | 0.249720064 |
| AL121895.2 | 2.148188982 | 4.827885 | 0.2805581 | 2.208270132 | 1.410331772 |
| AC026401.3 | 6.195738377 | 2.344836 | 2.339633 | 11.8395063 | 1.169607239 |
| B3GALT1-AS1 | 0.833180455 | 0.8289746 | 0.3709608 | 0.873387351 | 0 |
| riskScore | 0.058429716 | 6.923604165 | 1.68537E+11 | 1.38E+28 | 6.18E-08 |
| risk | low | low | high | high | low |

**Table 5b. 8 lncRNAs with risk scores**.

| id | futime | fustat | LNCTAM34A | CASC19 | AC090197.1 | AC099850.3 | AC024022.1 | AL033397.2 | LINC00462 | B3GALT1-AS1 | riskScore | risk |
| --- | --- | --- | --- | --- | --- | --- | --- | --- | --- | --- | --- | --- |
| TCGA-B9-A5W7 | 1.602739726 | 0 | 1.134386532 | 0.190287932 | 0.116441302 | 0.761448125 | 0.157999255 | 0.127670331 | 0.508159767 | 0.833180455 | 1.565115354 | high |
| TCGA-2Z-A9J8 | 7.643835616 | 0 | 1.475834 | 0.8167063 | 0.05934653 | 0.7936808 | 0.135625 | 0 | 0.04673564 | 0.8289746 | 1.965285662 | high |
| TCGA-BQ-5879 | 1.909589041 | 1 | 0.1041703 | 0.6523654 | 0.1877174 | 4.794755 | 0 | 0.4376931 | 7.54922 | 0.3709608 | 50.24414115 | high |
| TCGA-P4-A5EA | 0.550684932 | 1 | 1.107615909 | 5.025374243 | 0.249042641 | 0.485537014 | 0 | 0.522373917 | 1.6201391 | 0.873387351 | 137.0946489 | high |
| TCGA-MH-A857 | 2.438356164 | 0 | 1.086092137 | 0.052053365 | 0.03185258 | 0.178538177 | 1.426285751 | 0.174621434 | 0 | 0 | 0.017019303 | low |
| TCGA-5P-A9KF | 2.315068493 | 0 | 4.858394 | 0.2045421 | 0.1721001 | 0.1169267 | 0.01415289 | 0.4803186 | 1.482611 | 1.119491 | 0.708230909 | low |
| TCGA-Q2-A5QZ | 1.17260274 | 0 | 0.7032658 | 0 | 0.7544818 | 0.1392256 | 0 | 0.4902165 | 0.3019689 | 1.211806 | 4.285497584 | high |
| TCGA-DW-7842 | 0.252054795 | 0 | 0.528251 | 0 | 0.1180155 | 0.667453 | 0.1731193 | 0.3916863 | 0.07954128 | 0.06639368 | 1.41475517 | high |
| TCGA-2Z-A9JM | 2.55890411 | 0 | 1.780848 | 0.6740545 | 0.3093514 | 1.191001 | 0.5427187 | 0.2877922 | 0.771449 | 1.634226 | 1.103260269 | high |
| TCGA-Y8-A898 | 1.301369863 | 0 | 3.421633 | 0.08126905 | 0.1678397 | 0.1858302 | 0.1799441 | 0 | 0.1240156 | 0.3881875 | 0.318313224 | low |
| TCGA-KV-A6GD | 1.534246575 | 0 | 1.652743 | 0.05614984 | 0.1632067 | 0.3209812 | 0.2486515 | 0 | 0.2784728 | 0.1341019 | 0.502862513 | low |
| TCGA-SX-A7SM | 0.994520548 | 1 | 1.339749 | 1.135511 | 0.2798677 | 1.226108 | 0 | 0.1269752 | 10.51699 | 0 | 10.68198855 | high |
| TCGA-J7-8537 | 0.879452055 | 1 | 0.4596466 | 0.2056094 | 0.07077198 | 4.4664 | 0.01422675 | 0.0344875 | 0.3137574 | 0.102303 | 8.310125212 | high |
| TCGA-2Z-A9JT | 1.873972603 | 0 | 4.351063 | 0 | 0.3374487 | 0 | 0.03591249 | 0 | 0.049501 | 1.910996 | 0.585836793 | low |
| TCGA-BQ-5875 | 7.230136986 | 0 | 0.7549318 | 0.2827227 | 0.05406379 | 0.8619666 | 0.06520816 | 0 | 0 | 0.01875619 | 1.61817682 | high |
| TCGA-A4-A7UZ | 0 | 0 | 1.758036 | 0.9436872 | 0.2681076 | 1.233051 | 0.05596852 | 0.04522502 | 7.354566 | 0.3219707 | 5.228794645 | high |
| TCGA-AL-3471 | 4.109589041 | 1 | 2.510536 | 1.684522 | 0.1061113 | 0 | 0.1645514 | 0.5983412 | 0.03780234 | 1.774906 | 4.110602273 | high |
| TCGA-B3-3925 | 2.819178082 | 0 | 2.414274 | 0.06171168 | 0.2029746 | 1.481657 | 0.01708008 | 0.1656175 | 0.7769128 | 0.2210777 | 1.443979638 | high |
| TCGA-A4-8516 | 0.063013699 | 0 | 1.27361 | 0.05893574 | 0.09917615 | 1.01072 | 0.5709122 | 0.1977095 | 0 | 0.3753478 | 0.350867484 | low |
| TCGA-G7-6796 | 5.953424658 | 0 | 2.352444 | 0.2950365 | 0.6364007 | 0.3373158 | 0.3592948 | 0.07917981 | 0.2025999 | 0.7985824 | 0.555165238 | low |
| TCGA-G7-A8LD | 1.408219178 | 1 | 1.388343 | 0.2964028 | 0.1392703 | 7.697371 | 0.01171944 | 0.369323 | 0.03230764 | 0.185401 | 27.39578345 | high |
| TCGA-G7-6793 | 0.920547945 | 1 | 0.1291559 | 0.2696124 | 1.063543 | 6.076899 | 0 | 0.3617834 | 0.01469376 | 0 | 35.88682868 | high |
| TCGA-B1-A654 | 1.687671233 | 0 | 1.979981 | 0 | 0.1838829 | 0.5063036 | 0.07003813 | 0.04244541 | 0.02413476 | 1.460546 | 1.35193094 | high |
| TCGA-Y8-A8S1 | 1.106849315 | 0 | 1.036418405 | 0 | 0.093411372 | 0.155135952 | 0.938888394 | 0 | 0.207062954 | 0.189040392 | 0.071004968 | low |
| TCGA-HE-A5NK | 6.104109589 | 0 | 0.719606911 | 0 | 0.213389063 | 0.122674422 | 0.267274814 | 0.215969777 | 0.204669903 | 0 | 0.715588202 | low |
| TCGA-B9-A69E | 1.238356164 | 0 | 0.6640179 | 0.0468993 | 0.3372097 | 0.7506809 | 0.01298042 | 0.03146626 | 1.037732 | 0.03733632 | 2.048901034 | high |
| TCGA-A4-8630 | 1.194520548 | 0 | 2.709497 | 0.05231714 | 0.1000437 | 0.4187001 | 0.2461589 | 0.2457088 | 0.3193412 | 0.3956697 | 0.451481016 | low |
| TCGA-BQ-7062 | 0.317808219 | 0 | 3.029741 | 0.03305526 | 0.1011362 | 0.4157137 | 0.2104219 | 0.1330671 | 0.4539773 | 0.09210301 | 0.354725353 | low |
| TCGA-P4-A5E7 | 7.257534247 | 0 | 2.644394 | 0.2102919 | 0.2814923 | 0.8414953 | 0.4219716 | 0.2116375 | 0.1805079 | 0.1046328 | 0.330608871 | low |
| TCGA-2Z-A9J2 | 4.917808219 | 0 | 0.7691894 | 0 | 0.2368648 | 0.5245078 | 0 | 0 | 0 | 0.5478319 | 1.887637655 | high |
| TCGA-2Z-A9JD | 2.904109589 | 0 | 1.393183 | 0 | 0.05535716 | 0.1379043 | 0.05007611 | 0.04046369 | 0 | 0 | 0.833595378 | low |
| TCGA-GL-8500 | 2.304109589 | 0 | 0.3773362 | 0 | 0.1793922 | 0.3047026 | 1.017927 | 0.1430487 | 0 | 0.02121682 | 0.082983638 | low |
| TCGA-DZ-6132 | 6.739726027 | 0 | 1.25734 | 0.04065788 | 0.133727 | 1.487496 | 0.09002379 | 0.1091148 | 1.209847 | 0.04855133 | 1.647619882 | high |
| TCGA-5P-A9JY | 4.131506849 | 1 | 3.130786 | 0.1377507 | 0.08429262 | 0.07874533 | 0 | 0.5545283 | 5.885763 | 0.9321329 | 2.001116479 | high |
| TCGA-2Z-A9J1 | 6.295890411 | 0 | 1.209824 | 0.05903774 | 0.1490217 | 0.3374899 | 0.2451001 | 0 | 0.3378407 | 0.5874959 | 0.736930002 | low |
| TCGA-BQ-7045 | 4.021917808 | 1 | 1.95275533 | 0.1164677 | 0.08908638 | 0.26631554 | 0.09670508 | 0.15628389 | 0.13329619 | 0.0927194 | 0.772107519 | low |
| TCGA-BQ-7049 | 3.736986301 | 1 | 1.003399 | 0.09128968 | 0.3037503 | 1.356832 | 0.1389655 | 0.153123 | 0.03482673 | 0.03633765 | 1.55976136 | high |
| TCGA-B1-A657 | 1.756164384 | 0 | 1.593645 | 0.08819016 | 0.1214223 | 0.302484 | 0.1952687 | 0.2070937 | 0.4037313 | 0.3685909 | 0.798272661 | low |
| TCGA-5P-A9K0 | 1.854794521 | 0 | 2.08974 | 0.1475975 | 0.1053711 | 0.3937465 | 0.05446784 | 0.3300931 | 0.694465 | 0.3916722 | 1.22932402 | high |
| TCGA-2Z-A9J6 | 4.742465753 | 0 | 2.411633 | 0.1849325 | 0.1508856 | 0.1409558 | 0.9042541 | 0.3722317 | 0.6114436 | 0.981493 | 0.102197403 | low |
| TCGA-5P-A9K4 | 5.983561644 | 0 | 3.456747 | 0.5834997 | 0.2544025 | 0.8005398 | 0 | 0.3523399 | 9.772285 | 0.4412954 | 3.393103279 | high |
| TCGA-UZ-A9PN | 3.112328767 | 1 | 1.308396 | 0.04877271 | 0.2238381 | 1.672859 | 0.3644712 | 0.5562943 | 2.028124 | 0.6600715 | 1.573650652 | high |
| TCGA-2Z-A9JI | 4.293150685 | 0 | 1.137695 | 0.1110359 | 0.403425 | 2.158109 | 0.01536583 | 0.1489952 | 0.275339 | 0.9502481 | 4.496994269 | high |
| TCGA-UZ-A9PS | 5.936986301 | 0 | 1.560295761 | 0.294547806 | 0.073831581 | 0.964326597 | 0.049677722 | 0.199995821 | 0.26241725 | 0.095753314 | 1.607266838 | high |
| TCGA-DW-7840 | 0.312328767 | 0 | 1.0541 | 0.1886083 | 0.187547 | 0.6469089 | 0.3132093 | 0.04218114 | 0.04796899 | 0.4004006 | 0.749405896 | low |
| TCGA-MH-A561 | 2.304109589 | 0 | 2.254211 | 0 | 0.2575828 | 0.1957678 | 0.189567 | 0 | 0.1306475 | 0.4998236 | 0.513645623 | low |
| TCGA-2Z-A9JS | 1.347945205 | 1 | 2.909035 | 0.169731 | 0.08438796 | 0.9702694 | 0.2348842 | 0.2562256 | 0.4208871 | 0.4391469 | 0.591257891 | low |
| TCGA-2Z-A9JR | 0.44109589 | 0 | 2.501846 | 0.1882644 | 0.187205 | 0.3587385 | 0.1563191 | 0.5894592 | 0.02394076 | 0.7743616 | 1.094890953 | high |
| TCGA-Y8-A8RY | 2.106849315 | 0 | 2.228947 | 0.0942098 | 0.2450083 | 0.4308411 | 0.2216346 | 0.6320841 | 0.2515852 | 0.225 | 0.837099663 | low |
| TCGA-IZ-8196 | 1.778082192 | 0 | 0.898461 | 0.1269158 | 0.160179 | 0.6529642 | 0.07025356 | 0.1277279 | 0.024209 | 0.2020742 | 1.56048244 | high |
| TCGA-BQ-7058 | 0.939726027 | 1 | 1.233871 | 0 | 0.2841136 | 0.5628101 | 0 | 0.03002527 | 0 | 0 | 1.328881582 | high |
| TCGA-DW-7838 | 2.038356164 | 0 | 1.334817 | 0.04841281 | 0.070359 | 0.6642056 | 0.3081844 | 0.06496344 | 0.110816 | 0.1348943 | 0.537706397 | low |
| TCGA-IA-A83W | 5.597260274 | 1 | 2.778661 | 0.8860651 | 0.2993409 | 0.9286201 | 0.04087304 | 0.3467857 | 0.1690155 | 0 | 1.826370587 | high |
| TCGA-A4-7996 | 1.38630137 | 0 | 4.114477 | 0.03067484 | 0.2275935 | 1.017048 | 0.2037587 | 0.2675497 | 0.163833 | 0.9157539 | 0.463446417 | low |
| TCGA-UZ-A9PO | 5.235616438 | 0 | 0.74027296 | 0.06208863 | 0.13297681 | 0.56788852 | 0.08592205 | 0.20828635 | 0 | 0.34599933 | 1.638295521 | high |
| TCGA-DZ-6133 | 4.265753425 | 1 | 0.3984957 | 0.1572841 | 0.1130885 | 0.5394698 | 0.01741275 | 0.0422108 | 0.01200068 | 0.4257248 | 2.198722174 | high |
| TCGA-B1-A47O | 2.306849315 | 0 | 3.078473237 | 0.490141619 | 0.840870302 | 1.440977097 | 0.096898284 | 1.879153558 | 0.854799997 | 1.08698435 | 9.202769145 | high |
| TCGA-A4-7585 | 2.931506849 | 1 | 0.6336013 | 0.6613202 | 0.2456962 | 1.512178 | 0 | 0.06338587 | 0 | 0.733303 | 5.328518086 | high |
| TCGA-B9-A5W8 | 1.435616438 | 0 | 0.9756722 | 0 | 0.2336828 | 0.4158174 | 0.4429114 | 0.09760686 | 2.636248 | 0.02895388 | 0.487299824 | low |
| TCGA-SX-A7SO | 3.443835616 | 0 | 3.081484 | 0.04864981 | 0.0446548 | 0.4449714 | 0 | 0.359048 | 0.4825535 | 0.2130144 | 0.832005337 | low |
| TCGA-Y8-A897 | 1.504109589 | 0 | 2.370292296 | 0 | 0.084050211 | 1.057938448 | 0.416174316 | 0.349221227 | 0.088253181 | 0.598532693 | 0.432975186 | low |
| TCGA-F9-A8NY | 0.098630137 | 0 | 0.08790255 | 0.5308284 | 0.496261 | 1.011496 | 0.11427 | 0.2770055 | 15.34571 | 0.2347723 | 15.9365745 | high |
| TCGA-P4-A5ED | 7.715068493 | 0 | 5.050512 | 0 | 0.09036672 | 0.2132708 | 0.7400143 | 0 | 0 | 0 | 0.023227024 | low |
| TCGA-A4-8518 | 1.643835616 | 0 | 1.297402 | 0.3224193 | 0.2515516 | 0.1474491 | 0.07138938 | 0.04326431 | 0.1476024 | 0.4106825 | 1.355139832 | high |
| TCGA-BQ-5881 | 3.983561644 | 0 | 2.123983 | 0.04872315 | 0.09317109 | 0.2785264 | 0.2562191 | 0.06537987 | 0 | 0.4460652 | 0.448640015 | low |
| TCGA-B9-4117 | 0.038356164 | 0 | 2.582312 | 0 | 0.2356146 | 0.6603264 | 0 | 0.8718843 | 1.175134 | 0.05747418 | 1.778283953 | high |
| TCGA-BQ-5884 | 2.169863014 | 0 | 3.174314 | 0 | 0.2956016 | 0.4351424 | 0.226886 | 0 | 0.04467639 | 0.01165366 | 0.284705483 | low |
| TCGA-A4-A57E | 0.706849315 | 1 | 0.08276962 | 0.07404912 | 0.3624981 | 4.317686 | 0.04098948 | 0.6458653 | 0.7627367 | 6.749794 | 220.5028566 | high |
| TCGA-HE-A5NI | 0.98630137 | 0 | 1.869985 | 0.3273194 | 0.1335292 | 0.4989667 | 0.1811859 | 0.2196092 | 0.7492281 | 0.3474367 | 0.992075202 | low |
| TCGA-2Z-A9J3 | 4.852054795 | 1 | 1.55671 | 0.2198995 | 0.07400868 | 1.759881 | 0.1582414 | 0.1475377 | 0 | 0.4901707 | 1.606621053 | high |
| TCGA-B9-4113 | 4.208219178 | 1 | 2.739234 | 0.3430885 | 0.1799514 | 0.4482901 | 0.1085227 | 0.3617258 | 0.05609451 | 0.05852811 | 0.825252753 | low |
| TCGA-G7-A8LC | 1.923287671 | 0 | 0.4775651 | 0 | 0.2402009 | 0.2930851 | 2.146252 | 0.128995 | 0 | 0.1020394 | 0.002721868 | low |
| TCGA-BQ-7059 | 0.635616438 | 0 | 1.63821 | 0.06513824 | 0.1345258 | 0.7074906 | 0.2163415 | 0.08740671 | 0.9443014 | 0.1166766 | 0.724993303 | low |
| TCGA-A4-7915 | 0.019178082 | 0 | 0.065946732 | 1.283220984 | 0.426461491 | 7.537906655 | 0.012246895 | 0.653137882 | 3.848835007 | 0.017613213 | 175.8858867 | high |
| TCGA-5P-A9K8 | 3.58630137 | 0 | 0.2642433 | 0.08343632 | 0.0414834 | 0.5246611 | 1.905161 | 0.08397019 | 0.01591535 | 0.03321164 | 0.00613128 | low |
| TCGA-BQ-5886 | 2.169863014 | 0 | 1.04796 | 0.2163572 | 0.05295747 | 0.6926127 | 0.1556924 | 0.02903221 | 0.03301584 | 0.0344482 | 1.008845584 | low |
| TCGA-BQ-5882 | 1.802739726 | 1 | 1.516253 | 0.03989714 | 0.02746567 | 3.101785 | 0 | 0.08030485 | 0.5479429 | 0.3652622 | 3.495949807 | high |
| TCGA-BQ-5885 | 2.523287671 | 0 | 3.592265 | 0.3382936 | 0.6986559 | 0.1289239 | 0.8582775 | 0.6809163 | 1.161521 | 1.054813 | 0.144476624 | low |
| TCGA-SX-A71V | 2.4 | 0 | 1.712027 | 0 | 0.1640187 | 0.5837129 | 0.5369631 | 0.1370178 | 0.1168639 | 0.5487028 | 0.282298544 | low |
| TCGA-P4-AAVL | 1.334246575 | 1 | 1.002497435 | 0.185560457 | 0.198709802 | 1.37898589 | 0.077036977 | 0.124498515 | 0.424744415 | 0.036930959 | 1.965499752 | high |
| TCGA-A4-A4ZT | 1.41369863 | 0 | 2.537074765 | 0.153018317 | 0.169713834 | 0.174946175 | 0.296458564 | 0.05133247 | 0 | 0.761357407 | 0.404985024 | low |
| TCGA-PJ-A5Z8 | 1.643835616 | 0 | 0.4096526 | 0 | 0.1401653 | 0.9311361 | 0.1352465 | 0.2185702 | 0.06214027 | 0.01620904 | 1.570278144 | high |
| TCGA-Y8-A8RZ | 0.561643836 | 0 | 1.468085 | 0.2626819 | 0.07233334 | 0.5405847 | 0.08724369 | 0.07049673 | 0.02004247 | 0.04182398 | 1.06997804 | high |
| TCGA-A4-7288 | 5.087671233 | 0 | 3.853822 | 0.04556548 | 0.09758875 | 0.6772364 | 0 | 0.09171406 | 0 | 0.1269606 | 0.499120237 | low |
| TCGA-DW-7836 | 2.994520548 | 0 | 2.436487 | 0.1569443 | 0.09203605 | 0.7177391 | 0.0579171 | 0.03509967 | 0.3991584 | 0.3540042 | 0.892479865 | low |
| TCGA-J7-A8I2 | 0.84109589 | 0 | 2.967803 | 0.1234939 | 0.07084557 | 1.976672 | 0 | 0.4971364 | 0.04711255 | 0.6144559 | 2.068220106 | high |
| TCGA-AT-A5NU | 0.183561644 | 0 | 3.662901 | 0.2286266 | 0.04809117 | 0.3267367 | 0.2610197 | 0.2876119 | 0.06541526 | 0.1251309 | 0.285099995 | low |
| TCGA-DW-7839 | 1.147945205 | 0 | 1.241455 | 0 | 0.09267749 | 0.1484201 | 1.36533 | 0.1161313 | 0 | 0.2755913 | 0.019997007 | low |
| TCGA-DZ-6135 | 6.216438356 | 0 | 1.917538 | 0 | 0.2256977 | 0.8629916 | 0.1139532 | 0.1841584 | 3.481733 | 0.05462834 | 1.236371452 | high |
| TCGA-BQ-5889 | 0.901369863 | 1 | 0.97765279 | 0.46034131 | 0.20774845 | 0.684202 | 0.28030134 | 0.12354315 | 0.96590327 | 0.18323781 | 1.139719594 | high |
| TCGA-SX-A7SL | 2.254794521 | 0 | 3.572423 | 0.07609613 | 0.0698473 | 0.1740017 | 0.04212259 | 0 | 0.8999435 | 0.5149281 | 0.487491693 | low |
| TCGA-SX-A71R | 3.991780822 | 0 | 3.095181 | 0.1993735 | 0.3558362 | 0.1519628 | 0.6253853 | 0.5796533 | 0.07606038 | 1.163949 | 0.243489279 | low |
| TCGA-SX-A7SN | 1.583561644 | 0 | 1.582964 | 0.3034681 | 0.2205172 | 0.4336947 | 0.2099789 | 0.2545082 | 0.5788607 | 0.5435766 | 1.118656683 | high |
| TCGA-IZ-A6M8 | 1.361643836 | 0 | 3.628054 | 0.1064199 | 0.3256032 | 0.1825051 | 0.1472704 | 0 | 0.02029943 | 0.8472039 | 0.420263115 | low |
| TCGA-AL-3466 | 0.802739726 | 1 | 0.99597797 | 0.80193866 | 0.40893635 | 7.64048517 | 0 | 0.98641876 | 1.32572743 | 2.979292 | 310.1910534 | high |
| TCGA-F9-A97G | 0.035616438 | 0 | 1.143398 | 0.9055454 | 0.05387305 | 0.9202774 | 0.0696194 | 0.10126 | 0 | 0.02002502 | 2.400098273 | high |
| TCGA-B3-A6W5 | 0.846575342 | 0 | 1.679691625 | 0 | 0.17785989 | 0.813820094 | 0.499093891 | 0.445741316 | 0.561214397 | 0.207780098 | 0.412068756 | low |
| TCGA-IA-A40U | 1.4 | 0 | 2.517205867 | 0 | 0.712300377 | 0.208760443 | 0.101074061 | 0.245016827 | 0.325076033 | 0.60567699 | 0.967048608 | low |
| TCGA-HE-7130 | 4.849315068 | 0 | 6.623002932 | 0 | 0.051573383 | 6.587429815 | 0 | 0.164499978 | 0 | 0 | 1.334014504 | high |
| TCGA-A4-A5XZ | 1.312328767 | 0 | 1.016672 | 0 | 0.1123858 | 0.2399765 | 0.1936462 | 0.1408271 | 0.2402259 | 0.6405445 | 0.964042465 | low |
| TCGA-DZ-6134 | 5.084931507 | 0 | 1.73144 | 0 | 0.1892677 | 0.6209983 | 0.1920911 | 0 | 0.05755961 | 0.1441363 | 0.615954425 | low |
| TCGA-Y8-A895 | 1.326027397 | 0 | 3.387745 | 0.1122525 | 0.1287932 | 0.4491847 | 0.04660255 | 0.4518828 | 1.370367 | 0.6702275 | 0.97860072 | low |
| TCGA-2Z-A9J7 | 0.380821918 | 1 | 1.473016 | 0 | 0.2194848 | 0.2187098 | 0.01764853 | 0.1711295 | 2.238025 | 0.6599249 | 1.762010817 | high |
| TCGA-IA-A83S | 7.147945205 | 1 | 1.136718217 | 1.627127984 | 0.171131609 | 1.0851744 | 0.112585967 | 0.045487201 | 0.387965157 | 0.431783059 | 4.711878292 | high |
| TCGA-DW-7841 | 1.219178082 | 0 | 0.9603006 | 0.04686134 | 0.4193787 | 0.8036501 | 0.1426691 | 0.1886447 | 1.465951 | 0.7088159 | 2.012144388 | high |
| TCGA-UZ-A9PL | 8.635616438 | 0 | 1.948945 | 0.2696299 | 0.4908524 | 0.3699222 | 0.5671582 | 0.1809035 | 0.3291615 | 0.9659303 | 0.382217441 | low |
| TCGA-BQ-7044 | 10.30136986 | 0 | 0.313222 | 0.19106 | 0.05553402 | 1.179572 | 0.0528801 | 0.1281883 | 1.049598 | 0.2889937 | 2.887514751 | high |
| TCGA-EV-5902 | 1.583561644 | 0 | 3.713774 | 0.1124153 | 0.154776 | 0.6426231 | 0.05185571 | 0.05028203 | 0.600405 | 0.1193244 | 0.489382231 | low |
| TCGA-UZ-A9PM | 10.44109589 | 0 | 0.9130437 | 0 | 0.09372114 | 0.4669512 | 0.3202806 | 0.1826829 | 3.090275 | 0.7315738 | 1.030323018 | low |
| TCGA-BQ-5883 | 3.402739726 | 0 | 1.43525 | 0.03852102 | 0.06482258 | 0.3523294 | 7.1539 | 0.05169 | 0 | 0 | 4.11E-10 | low |
| TCGA-A4-8517 | 1.635616438 | 0 | 1.70526 | 0.05581447 | 0.11527 | 0.5743153 | 0.03089579 | 0.07489547 | 0.02129305 | 0.1333009 | 1.062177233 | high |
| TCGA-O9-A75Z | 1.290410959 | 0 | 2.746317 | 0.05302809 | 0.1541329 | 0.5456442 | 0.04403011 | 0.07115653 | 0.0404601 | 0.02110771 | 0.639026173 | low |
| TCGA-G7-A4TM | 2.104109589 | 0 | 1.183747 | 0.1512898 | 0.3008769 | 0.8648498 | 0.1395761 | 0.1353403 | 1.539109 | 0.1003676 | 1.463782802 | high |
| TCGA-GL-A59R | 1.035616438 | 0 | 2.489089 | 0 | 0.1411959 | 0.6029887 | 0.09731492 | 0.1179521 | 0.0335342 | 0.03498904 | 0.616214491 | low |
| TCGA-SX-A71W | 2.947945205 | 0 | 2.46877 | 0.04388074 | 0.05370314 | 0.4515205 | 0 | 0.117764 | 1.573594 | 0.1571996 | 0.948870388 | low |
| TCGA-UZ-A9PV | 3.956164384 | 0 | 6.368614 | 0 | 0.1581578 | 0.893063 | 0.01271729 | 0.5549111 | 4.17196 | 0.6218508 | 0.484499715 | low |
| TCGA-MH-A855 | 1.010958904 | 0 | 3.388273 | 0.04639727 | 0.06033193 | 0.3713227 | 0.06420739 | 0.09338829 | 6.053542 | 0.2585566 | 0.805404428 | low |
| TCGA-B3-8121 | 1.038356164 | 0 | 2.58488921 | 0.42913271 | 0.16959298 | 1.96251343 | 0.01979534 | 0.52785153 | 5.37523658 | 0.22775373 | 4.254273922 | high |
| TCGA-5P-A9KA | 1.082191781 | 0 | 1.064969 | 0.1329439 | 0.2593072 | 0.3039902 | 0.1471808 | 0.1783928 | 3.651674 | 0.1852131 | 1.542079912 | high |
| TCGA-B1-7332 | 3.126027397 | 0 | 0.8811888 | 0.05631056 | 0.07752971 | 0.2575199 | 0.0311704 | 0.1133417 | 0.3437168 | 0.3586286 | 1.51742368 | high |
| TCGA-B9-A8YH | 1.457534247 | 0 | 1.185197 | 0.1255649 | 0.1856867 | 1.148469 | 0.2432701 | 0.2808185 | 2.123682 | 0.1999233 | 1.36868783 | high |
| TCGA-IA-A40Y | 0.169863014 | 0 | 1.640516 | 0.2786722 | 0.3282617 | 5.225147 | 0 | 0.1495762 | 3.316952 | 0.7764739 | 15.90484847 | high |
| TCGA-A4-7583 | 0.008219178 | 0 | 0.3764071 | 0.09621409 | 0.1324698 | 1.155019 | 0.8121964 | 0.225936 | 4.936873 | 0.0957445 | 0.386158312 | low |
| TCGA-A4-A48D | 1.038356164 | 0 | 0.954817 | 0.1220312 | 0.07467355 | 0.4185557 | 0.05066229 | 0 | 0 | 0 | 1.178150805 | high |
| TCGA-HE-A5NF | 7.073972603 | 0 | 2.310957213 | 0 | 0.240862103 | 0.545481154 | 0.343332256 | 0.128043474 | 0 | 0.379825272 | 0.371911118 | low |
| TCGA-GL-A9DC | 1.073972603 | 0 | 3.991463 | 0.2434723 | 0.1536418 | 0.4175436 | 0.539091 | 0.2858685 | 0.02322098 | 0.5087964 | 0.136457654 | low |
| TCGA-A4-8310 | 2.136986301 | 0 | 2.006756 | 0.5026914 | 0.2526778 | 1.313664 | 0.01987586 | 0.09636341 | 0.1917752 | 1.257741 | 3.063008845 | high |
| TCGA-SX-A71U | 3.717808219 | 0 | 3.744185 | 0.4480494 | 0.4504239 | 0.4390764 | 0.1062923 | 0.4723884 | 0.3174404 | 0.8917251 | 1.051005044 | low |
| TCGA-B9-4617 | 0.095890411 | 0 | 1.188128 | 0 | 0.2032628 | 0 | 0.3105387 | 0.3565832 | 0.9687218 | 0.5406331 | 0.738922105 | low |
| TCGA-BQ-7051 | 4.2 | 0 | 1.601963 | 0.2433705 | 0.1634024 | 0.7110731 | 0.02993698 | 0.1995707 | 0.03094836 | 0.6458204 | 1.871595756 | high |
| TCGA-SX-A7SS | 4.424657534 | 0 | 2.439263 | 0.3519783 | 0.08076868 | 0.08048352 | 0.3312206 | 0.5667687 | 0.295413 | 0.1120833 | 0.495177725 | low |
| TCGA-G7-6795 | 3.271232877 | 0 | 1.040988 | 0.1470491 | 0.5024025 | 0.1681216 | 0.5969204 | 0 | 0 | 0.7804342 | 0.33473452 | low |
| TCGA-SX-A71S | 4.120547945 | 0 | 1.510427 | 0.2105907 | 0.1409461 | 0.5417298 | 0.02914283 | 0.2472612 | 0.02008492 | 0.2305191 | 1.555403541 | high |
| TCGA-2Z-A9JN | 2.02739726 | 0 | 1.827602 | 0.04584247 | 0.0175325 | 0.2096472 | 2.613712 | 0.06151439 | 0 | 0.01824749 | 0.000315071 | low |
| TCGA-PJ-A5Z9 | 1.479452055 | 0 | 2.342370503 | 0 | 0.237143496 | 1.181531146 | 0.548217977 | 0 | 0 | 1.508310367 | 0.352164387 | low |
| TCGA-UZ-A9PK | 9.783561644 | 0 | 1.50195453 | 0.051026979 | 0.097576602 | 0.291696284 | 0.734388291 | 0.068471303 | 0.116799801 | 0.426534631 | 0.137779841 | low |
| TCGA-G7-7502 | 2.153424658 | 0 | 1.692649541 | 0.080406057 | 0.384392233 | 0.919284205 | 0.233668734 | 0.107894051 | 0.13803597 | 0.896152542 | 1.063101718 | high |
| TCGA-BQ-7061 | 0.597260274 | 0 | 1.5286 | 0.03230429 | 0.2075608 | 2.105213 | 0 | 0.021674 | 0.1725358 | 0.0771519 | 2.133262816 | high |
| TCGA-BQ-7050 | 5.106849315 | 0 | 3.2554 | 0.051095 | 0.0234496 | 0.5257532 | 0.3111169 | 0.3770942 | 0.2923888 | 0.4677797 | 0.340882277 | low |
| TCGA-UZ-A9PR | 6.002739726 | 0 | 1.855897 | 0.04836005 | 0.2441387 | 0.8293523 | 0.06692361 | 0.09733898 | 0.1106953 | 0.2309953 | 1.106386093 | high |
| TCGA-IA-A40X | 1.665753425 | 0 | 3.234984 | 0 | 0.1099541 | 0.7888741 | 0.1750575 | 0.1157352 | 0.06580784 | 0.09155046 | 0.387695041 | low |
| TCGA-UZ-A9PQ | 7.189041096 | 1 | 0.6262145 | 0.04802034 | 0.1065195 | 0.93333 | 0.2259419 | 0.9665521 | 5.569162 | 1.242435 | 5.637714042 | high |
| TCGA-BQ-5877 | 0.739726027 | 1 | 0.2596137 | 0.1161306 | 0.1658132 | 3.098021 | 0.01071391 | 0 | 36.96385 | 0 | 145.7339612 | high |
| TCGA-BQ-7048 | 7.778082192 | 0 | 3.170195 | 0 | 0.2723296 | 0.7589109 | 0.03340332 | 0.08097404 | 4.972581 | 0.1200998 | 1.003421375 | low |
| TCGA-P4-A5EB | 6.78630137 | 0 | 1.027386572 | 0.237709239 | 0.098185113 | 0.163064095 | 0.06579132 | 0.127589456 | 0 | 1.324674698 | 2.076041988 | high |
| TCGA-P4-AAVM | 4.216438356 | 0 | 1.032599 | 0.3959168 | 0.007570939 | 0.2263263 | 0.05478939 | 0.199225 | 0.03776025 | 0 | 1.478043089 | high |
| TCGA-5P-A9KE | 2.257534247 | 0 | 2.893025 | 0.09298993 | 0.1458128 | 0.2126312 | 0.1801592 | 0.2183648 | 0.03547537 | 0.1295505 | 0.426920786 | low |
| TCGA-5P-A9JW | 8.879452055 | 0 | 1.868424 | 0 | 0.1150728 | 0.3185182 | 0.0925288 | 0.2990693 | 2.019379 | 0.1552517 | 1.051343895 | low |
| TCGA-AL-3472 | 5.55890411 | 0 | 2.712169 | 0.1465622 | 0.2466325 | 0 | 0.7842439 | 0.3605554 | 0.03727534 | 0.1750162 | 0.08504154 | low |
| TCGA-B9-4115 | 2.2 | 0 | 2.808803 | 0 | 0.1543982 | 1.224545 | 0.1710228 | 0.1658327 | 0.2200186 | 0.7378838 | 0.767032998 | low |
| TCGA-B9-A44B | 0.989041096 | 0 | 2.870009604 | 0.431361661 | 0.282813785 | 0.704538183 | 0.017055562 | 0.289414497 | 0.86983368 | 1.079273026 | 1.939845184 | high |
| TCGA-2K-A9WE | 0.58630137 | 0 | 2.362033078 | 0 | 0.141611153 | 1.026263097 | 0.186329442 | 0.677530641 | 0.535067655 | 0.08932496 | 0.985765552 | low |
| TCGA-A4-A6HP | 1.038356164 | 0 | 2.528626 | 0 | 0.3097219 | 0 | 0.3630431 | 0.07652732 | 0.1522989 | 0.3405134 | 0.258862153 | low |
| TCGA-2Z-A9J5 | 8.356164384 | 0 | 1.982277 | 0.171622 | 0.07876439 | 0.09810788 | 0.3562515 | 0.1151467 | 1.080308 | 0.1366275 | 0.36338089 | low |
| TCGA-SX-A7SU | 3.646575342 | 0 | 3.1051 | 0 | 0.3374777 | 0.3202726 | 0.1550641 | 0.5168567 | 1.255705 | 0.3345146 | 0.69545406 | low |
| TCGA-DW-7963 | 3.397260274 | 0 | 2.527696303 | 0.128002718 | 0.04895477 | 0.585383144 | 0.141710399 | 0.085881165 | 1.855640227 | 0.509511902 | 0.774365492 | low |
| TCGA-A4-8312 | 1.939726027 | 0 | 0.8253933 | 0 | 0.09743271 | 0.4432311 | 6.36124 | 0.03715779 | 0 | 0 | 5.88E-09 | low |
| TCGA-B9-A8YI | 1.024657534 | 0 | 0.5735718 | 0.1231538 | 0.1978213 | 0.4928072 | 0.06817113 | 0.2065697 | 0 | 1.127485 | 2.653488268 | high |
| TCGA-WN-A9G9 | 2.167123288 | 0 | 1.161475 | 0.06776761 | 0.4302353 | 1.936971 | 0 | 0.1818699 | 5.635982 | 0 | 4.836362259 | high |
| TCGA-HE-A5NH | 2.167123288 | 0 | 2.009913 | 0 | 0.1404673 | 0.1312232 | 0.7624008 | 0.1540133 | 0.04378653 | 0.4111755 | 0.100827786 | low |
| TCGA-Y8-A896 | 1.553424658 | 0 | 3.323301 | 0.05868084 | 0.3635693 | 1.811428 | 0.2598596 | 0.1181126 | 4.365379 | 0.0934311 | 0.732018292 | low |
| TCGA-UZ-A9PJ | 2.183561644 | 1 | 1.712434 | 0.05344236 | 0.200303 | 0.5499069 | 0.07395681 | 1.003974 | 0.4689261 | 0.2552713 | 2.260874866 | high |
| TCGA-BQ-7060 | 0.58630137 | 0 | 1.000839 | 0 | 0.1917684 | 0.6142222 | 0.1685174 | 0.0720897 | 0.04099071 | 0.07128175 | 0.917499188 | low |
| TCGA-BQ-7053 | 4.153424658 | 0 | 1.658492 | 0.09146436 | 0.1305944 | 1.673142 | 0.06750612 | 0.1840992 | 0.2675158 | 0.1699002 | 1.702184975 | high |
| TCGA-5P-A9KH | 5.619178082 | 0 | 0.2634965 | 0.1248008 | 0.09546039 | 0.3804934 | 0.0460552 | 0.2511987 | 0 | 0 | 1.94872932 | high |
| TCGA-F9-A4JJ | 0.890410959 | 1 | 0.09245689 | 0.1240736 | 4.223235 | 3.262634 | 0 | 0.7075825 | 3.881363 | 0.2469361 | 100.9092353 | high |
| TCGA-A4-7584 | 1.780821918 | 0 | 2.298502 | 0.3136782 | 0.2039432 | 0.6574865 | 0.02893914 | 0.2104569 | 0.2792236 | 0.1872884 | 1.273915549 | high |
| TCGA-IA-A83T | 7.353424658 | 1 | 1.727042 | 0 | 0.1614474 | 0.2365844 | 0.4581815 | 0.05553462 | 0.4105059 | 0.7248412 | 0.323012383 | low |
| TCGA-BQ-7055 | 2.189041096 | 0 | 0.1150617 | 0.06176336 | 0.08976155 | 0.5649135 | 0.01709438 | 0.08287808 | 0.02356253 | 0 | 2.006741365 | high |
| TCGA-A4-8515 | 1.942465753 | 0 | 1.330951018 | 0.051031014 | 0.109294435 | 0.466750958 | 0.169487622 | 0.136953435 | 0.408831628 | 0.690634484 | 1.061450522 | high |
| TCGA-G7-6792 | 7.575342466 | 0 | 0.6928818 | 0.2267856 | 0.1873463 | 0.8297103 | 0.3012862 | 0 | 0.0692144 | 0.1985973 | 0.883744454 | low |
| TCGA-A4-A5DU | 1.35890411 | 0 | 1.157894 | 0.07579756 | 0.278293 | 0.3466381 | 0.02097866 | 0.2034201 | 0.7807464 | 0.9353024 | 2.267403812 | high |
| TCGA-P4-AAVO | 6.575342466 | 0 | 1.037197 | 0.08979867 | 0.06868719 | 0.308001 | 0.447368 | 0 | 0 | 0.3931857 | 0.374968883 | low |
| TCGA-MH-A560 | 2.531506849 | 0 | 1.079397 | 0.06437818 | 0.0640159 | 0.07360371 | 0.1425447 | 0 | 0 | 0.05125117 | 0.72712453 | low |
| TCGA-GL-A9DD | 0.934246575 | 0 | 2.61659 | 0.05618182 | 0.1590023 | 0.5138623 | 0.4820366 | 0.2638594 | 0.3643642 | 0.51435 | 0.261574846 | low |
| TCGA-J7-6720 | 1.063013699 | 0 | 2.009369 | 0 | 0.1128568 | 0.9888583 | 0.1267328 | 0.1024057 | 0.8152004 | 0.202516 | 0.885282409 | low |
| TCGA-G7-A8LE | 6.720547945 | 0 | 0.9024853 | 0.04749415 | 0.2869942 | 0.5430017 | 0.2760463 | 0.4779805 | 0.2899019 | 0.01890494 | 1.018879594 | low |
| TCGA-F9-A7VF | 0.2 | 0 | 4.131317 | 0 | 0.1434344 | 0.2796418 | 0.2030883 | 0 | 0.8086938 | 2.076992 | 0.449624742 | low |
| TCGA-IZ-A6M9 | 1.030136986 | 0 | 5.999756 | 0 | 0.1393544 | 0 | 0.1301265 | 0 | 0.5380906 | 0.2573244 | 0.11015809 | low |
| TCGA-MH-A856 | 2.594520548 | 0 | 2.685913 | 0.08685283 | 0.4384638 | 0.9929903 | 0.4086537 | 0.5244514 | 1.126559 | 0.2765726 | 0.53085292 | low |
| TCGA-WN-AB4C | 3.254794521 | 0 | 2.764533 | 0 | 0.3289162 | 0.385594 | 0.6067436 | 0 | 0 | 0.6041113 | 0.134195452 | low |
| TCGA-AL-A5DJ | 4.104109589 | 1 | 0 | 0.4725595 | 0.1032748 | 1.929566 | 0.0934224 | 1.268222 | 0 | 1.262966 | 16.64911793 | high |
| TCGA-V9-A7HT | 2.643835616 | 0 | 2.523106816 | 0.68287323 | 0.178463136 | 0.780730453 | 0.330750627 | 0.305441164 | 3.842581613 | 0.498329579 | 1.07631991 | high |
| TCGA-MH-A55W | 2.857534247 | 0 | 1.932281 | 0 | 0.1897192 | 0.2577946 | 0.436851 | 0.2269252 | 0.1720417 | 0.4487638 | 0.326306836 | low |
| TCGA-P4-A5E6 | 8.315068493 | 0 | 6.0108 | 0.05680468 | 0.09993503 | 0.1298898 | 1.839469 | 0.07622419 | 0.5417702 | 0.8366059 | 0.000891362 | low |
| TCGA-UZ-A9Q0 | 9.22739726 | 0 | 2.214241 | 0.2377141 | 0.1636452 | 0.4529651 | 0.2412398 | 0.4253069 | 1.995118 | 0.9462157 | 1.133860172 | high |
| TCGA-5P-A9K2 | 6.553424658 | 0 | 7.688123 | 0 | 0.09326463 | 0 | 0.07787742 | 0.2517135 | 0.8229749 | 0.5226741 | 0.08909922 | low |
| TCGA-A4-7828 | 0.652054795 | 0 | 2.913798 | 0 | 0.4354435 | 0.8546635 | 0.1750677 | 0.07716129 | 1.908539 | 0.2060006 | 0.629515197 | low |
| TCGA-2Z-A9JL | 2.756164384 | 0 | 1.814008912 | 0.20008198 | 0.188753176 | 0.762513881 | 0.406099022 | 0.223735792 | 0.661532313 | 0.053094784 | 0.466295781 | low |
| TCGA-4A-A93Y | 0.964383562 | 0 | 0.573352336 | 0 | 0.058852861 | 1.817997257 | 0.042590652 | 0.034415116 | 0.293530172 | 0.061252934 | 2.315665993 | high |
| TCGA-BQ-5876 | 1.605479452 | 0 | 1.49595 | 0.6691695 | 0.0454977 | 2.040168 | 0 | 0.1995411 | 0 | 0 | 3.47897504 | high |
| TCGA-MH-A854 | 1.468493151 | 0 | 1.057698 | 0.2365651 | 0.8444294 | 0.09015515 | 0.3710231 | 0 | 0.03008296 | 0.3452688 | 0.671537194 | low |
| TCGA-Y8-A8S0 | 0.501369863 | 0 | 3.560849 | 0.1111284 | 0.144504 | 0.6987937 | 0.1076504 | 0.07455967 | 0.2331734 | 0.7077508 | 0.558432208 | low |
| TCGA-Y8-A894 | 1.553424658 | 0 | 1.461891 | 0.05770006 | 0.3133578 | 0.2638744 | 0.6707306 | 0.6581183 | 0 | 0.4593476 | 0.30441453 | low |
| TCGA-A4-7997 | 2.539726027 | 0 | 1.437325 | 0.3413867 | 0.6110381 | 1.795418 | 0.01889727 | 0.2061427 | 2.839188 | 0.5979081 | 5.163501838 | high |
| TCGA-DW-5560 | 4.071232877 | 0 | 2.421012 | 0.07062839 | 0.2863262 | 0.4844976 | 0.2736717 | 0.4738688 | 0.3233341 | 1.349447 | 0.93668354 | low |
| TCGA-BQ-5894 | 0.230136986 | 0 | 1.30038 | 7.334314 | 0.06577279 | 7.170909 | 0 | 0.271494 | 1.871778 | 0.4228107 | 5158.91637 | high |
| TCGA-P4-AAVK | 4.356164384 | 0 | 1.368739 | 0.04535298 | 0.1283552 | 0.4666694 | 0.1004196 | 0 | 2.266565 | 0.0722106 | 1.067843654 | high |
| TCGA-UN-AAZ9 | 1.501369863 | 0 | 0.3420662 | 0.04173089 | 0.1212962 | 1.097353 | 0.03464986 | 0.0279986 | 0 | 0 | 2.007122547 | high |
| TCGA-AL-7173 | 5.643835616 | 0 | 1.771273 | 0.3127607 | 0.1770312 | 0.429096 | 1.229201 | 0.7554286 | 0.02386345 | 0.5228735 | 0.066390468 | low |
| TCGA-B9-A5W9 | 0.01369863 | 0 | 1.811546 | 0 | 0.5885711 | 1.015084 | 0.02340315 | 0.05673231 | 0.03225839 | 1.11071 | 2.119167272 | high |
| TCGA-HE-A5NL | 2.101369863 | 0 | 2.216108 | 0 | 0.1447579 | 0.5151673 | 0.1745974 | 0.3023194 | 0.2750415 | 0.1076152 | 0.648816129 | low |
| TCGA-HE-7129 | 6.82739726 | 0 | 1.356332 | 0.1323742 | 0.005062667 | 0.9458985 | 0.8426629 | 0.06661063 | 0.1515011 | 0.0790369 | 0.118749223 | low |
| TCGA-5P-A9KC | 1.032876712 | 0 | 0.7818267 | 0.2468663 | 0.08182568 | 1.364173 | 0 | 0.4692866 | 0 | 0 | 3.38597649 | high |
| TCGA-4A-A93W | 0.010958904 | 0 | 1.775677 | 0.2803401 | 0.2487419 | 0.6410268 | 0.1241446 | 0.3009428 | 0.04277955 | 0.3570839 | 1.321709006 | high |
| TCGA-DW-5561 | 1.134246575 | 0 | 0.9297759 | 0.1919575 | 0.2973281 | 0.7132626 | 0.2523604 | 0.2897786 | 1.428008 | 0.2101227 | 1.321120276 | high |
| TCGA-BQ-5891 | 0.079452055 | 0 | 1.019437 | 0.1800061 | 0.275374 | 4.116027 | 0 | 0 | 0.3021557 | 1.232397 | 9.985373256 | high |
| TCGA-BQ-7056 | 1.676712329 | 0 | 3.662437 | 0.1064589 | 0.138432 | 2.028578 | 0.00982162 | 0.1904712 | 3.709387 | 0.2542543 | 1.470482291 | high |
| TCGA-A4-A5Y0 | 0.690410959 | 0 | 2.446856 | 0 | 0.07009178 | 0.291018 | 0.01409001 | 0.1707802 | 0 | 3.667773 | 3.137444864 | high |
| TCGA-B9-5155 | 1.821917808 | 0 | 1.37982 | 0.04256704 | 0.05860731 | 0.3893359 | 0.2591903 | 0.1713576 | 0.06495677 | 0.1524934 | 0.600879376 | low |
| TCGA-BQ-5887 | 2.112328767 | 0 | 1.178652 | 0 | 0.3288312 | 0.6027895 | 0.1459242 | 0.02721072 | 0.06188883 | 0.177578 | 0.974508106 | low |
| TCGA-A4-7732 | 1.594520548 | 0 | 2.063499 | 0.490733 | 0.2734785 | 1.041961 | 0.0970152 | 0.2351776 | 0.9628103 | 0.9487708 | 2.281768938 | high |
| TCGA-G7-A8LB | 1.498630137 | 0 | 1.401736 | 0.198008 | 0.08582549 | 0.150922 | 0.1826771 | 0.04428333 | 0 | 0.2627222 | 0.730883758 | low |
| TCGA-KV-A6GE | 2.424657534 | 0 | 2.513468 | 0.2044229 | 0.1993634 | 0 | 0.3394715 | 0.1028655 | 0.3509401 | 1.485004 | 0.511284221 | low |
| TCGA-P4-A5E8 | 3.578082192 | 1 | 0 | 2.400336 | 1.580291 | 1.470166 | 0 | 0.1725496 | 3.172319 | 0.03412316 | 49.51834426 | high |
| TCGA-AL-3467 | 5.849315068 | 0 | 0.3646856 | 0 | 0.4403983 | 6.450999 | 2.852433 | 0.2317767 | 0 | 0.8479626 | 0.005135032 | low |
| TCGA-HE-A5NJ | 4.728767123 | 0 | 0.9757164 | 0 | 0.2083209 | 1.077847 | 0.05798375 | 0.3514006 | 0.03996177 | 0.08339095 | 1.960054792 | high |
| TCGA-A4-A5Y1 | 1.082191781 | 1 | 1.394271 | 0.05627245 | 0.1312809 | 3.956689 | 0.01557465 | 0.15102 | 17.00247 | 1.131156 | 33.25665966 | high |
| TCGA-5P-A9K3 | 1.290410959 | 1 | 0.102893 | 0.05523137 | 0.02957261 | 2.588992 | 0 | 0.07411302 | 0 | 0.08793888 | 4.429416504 | high |
| TCGA-5P-A9K6 | 5.243835616 | 0 | 2.272421 | 0.10081 | 0.1465085 | 0.4033969 | 0.1255563 | 0.06763668 | 0.07691738 | 0.541717 | 0.732925573 | low |
| TCGA-EV-5903 | 2.073972603 | 0 | 1.4499 | 0.09156287 | 0.1645861 | 0.4710781 | 0.1140393 | 0.06143249 | 0.2619821 | 0.3462407 | 1.02061433 | low |
| TCGA-F9-A7Q0 | 1.090410959 | 0 | 0.8987609 | 0 | 0.1047219 | 0.745373 | 0.1623969 | 0.1749651 | 0 | 0.02595062 | 1.047786095 | low |
| TCGA-UZ-A9PZ | 1.8 | 0 | 2.515916 | 0.08135574 | 0.08712084 | 0.465071 | 0 | 0.4366736 | 0.5276284 | 0.4209851 | 1.285029252 | high |
| TCGA-SX-A7SP | 1.35890411 | 1 | 3.781593 | 0.09575008 | 0.1830986 | 0.6568276 | 0.01325048 | 0.2890882 | 0.9314731 | 0.8575447 | 0.910422793 | low |
| TCGA-KV-A74V | 0.950684932 | 0 | 3.383594 | 0.2018068 | 0.185235 | 0.05768155 | 0.6981817 | 0.03384969 | 0.05774151 | 1.526246 | 0.114237371 | low |
| TCGA-AL-3473 | 5.221917808 | 0 | 2.598291 | 0.03962281 | 0.5546285 | 1.08722 | 0.01096649 | 0.07975267 | 11.41256 | 0.06308707 | 3.155521044 | high |
| TCGA-GL-7773 | 1.076712329 | 0 | 1.191501 | 0 | 0.3304353 | 0.3848594 | 0.4347808 | 0.07528329 | 0.0214033 | 0.7369515 | 0.484714164 | low |
| TCGA-G7-6797 | 2.095890411 | 0 | 0.8622519 | 0 | 0.05900501 | 0.7215956 | 0.3299604 | 0 | 1.578459 | 0.02791422 | 0.621929275 | low |
| TCGA-SX-A7SR | 2.205479452 | 0 | 2.728594 | 0 | 0.195057 | 0.07475705 | 0.3800432 | 0.2193511 | 0.02494492 | 0.4945154 | 0.261839215 | low |
| TCGA-DW-7837 | 3.128767123 | 0 | 1.498089 | 0.0681484 | 0.2189328 | 0.5453995 | 0.2829238 | 0.04572297 | 0.2339856 | 0.1085053 | 0.555204016 | low |
| TCGA-5P-A9JU | 1.021917808 | 0 | 3.475192 | 0.3657706 | 1.580749 | 0.9409191 | 0 | 0.06135183 | 0.03488508 | 0.2547897 | 1.627992451 | high |
| TCGA-B3-4104 | 2.873972603 | 0 | 6.323115 | 0 | 0.1468435 | 7.813759 | 0 | 1.700206 | 0.2343635 | 0.03056639 | 9.127074439 | high |
| TCGA-A4-8098 | 2.454794521 | 0 | 0.2492327 | 0.1672304 | 0.2515659 | 3.377776 | 0.03085649 | 0.1122003 | 5.167628 | 0.3106398 | 11.04139575 | high |
| TCGA-B3-3926 | 2.419178082 | 0 | 0.4999862 | 0 | 0.1294208 | 1.600931 | 0.2745186 | 0.0782905 | 0 | 0 | 1.109275888 | high |
| TCGA-B9-4116 | 1.717808219 | 0 | 2.022563 | 0.6657478 | 0.2742018 | 0.5269507 | 0.1559127 | 0.9964216 | 0.03907396 | 0.285384 | 2.421713759 | high |
| TCGA-5P-A9JZ | 7.517808219 | 0 | 2.018142 | 0.04231671 | 0.1229989 | 0.8708542 | 0.05856046 | 0.1987416 | 0 | 0.9095798 | 1.438741292 | high |
| TCGA-BQ-5893 | 0.660273973 | 1 | 0.7804703 | 0.3054804 | 0.5975086 | 4.88959 | 0.04831343 | 0.6734291 | 5.777046 | 0.8859121 | 40.25039341 | high |
| TCGA-2Z-A9JE | 4.734246575 | 0 | 1.33965 | 0 | 0.1036968 | 0.7412851 | 0.09788256 | 0.0395467 | 0 | 1.407725 | 1.654896782 | high |
| TCGA-A4-7734 | 0.073972603 | 0 | 1.58703 | 0.2592719 | 0.2804777 | 1.143358 | 0.06150793 | 0.07455166 | 0.0282604 | 0.2506348 | 1.642806293 | high |
| TCGA-B3-4103 | 2.547945205 | 0 | 1.799338 | 0.0596208 | 0.08208733 | 1.499621 | 0.08250692 | 0.4800183 | 0.2729418 | 0.308515 | 1.862496359 | high |
| TCGA-GL-A9DE | 1.35890411 | 0 | 1.144035 | 0.05293972 | 0.6357511 | 0.3026305 | 0 | 0.2486328 | 0 | 0.1053627 | 1.908816128 | high |
| TCGA-B1-A47N | 3.04109589 | 0 | 2.086633 | 0 | 0.1285119 | 0 | 0.2325038 | 0.09393663 | 0.2670652 | 0.1950561 | 0.410909961 | low |
| TCGA-IZ-8195 | 1.854794521 | 0 | 2.661075 | 0 | 0.115011 | 0.7162811 | 0 | 0.04203404 | 0.6453231 | 0.1995021 | 0.843242322 | low |
| TCGA-2Z-A9JK | 3.249315068 | 0 | 2.106795475 | 0.133570354 | 0.551708478 | 0.916267662 | 0.036968567 | 0.298722242 | 24.66302998 | 0 | 14.16417049 | high |
| TCGA-2Z-A9JJ | 3.323287671 | 0 | 1.627561186 | 0.252014426 | 0.141361989 | 1.392621773 | 0.348752995 | 0.25362695 | 0.016023786 | 0.11703273 | 0.760633837 | low |
| TCGA-2Z-A9JO | 2.6 | 0 | 2.983801 | 0 | 0.02355985 | 0.07043 | 0.1534482 | 0.1239928 | 0.02350107 | 0.9317843 | 0.477442264 | low |
| TCGA-BQ-5890 | 0.638356164 | 0 | 1.54612 | 0.07410121 | 0.660324 | 1.990922 | 0.02050916 | 0.1242923 | 7.180423 | 0.2654624 | 5.391349544 | high |
| TCGA-BQ-7046 | 8.076712329 | 0 | 1.762121 | 0.1126047 | 0.1205842 | 0.4505942 | 0.1246634 | 0.1511003 | 1.267271 | 0.2913433 | 0.993299443 | low |
| TCGA-G7-6790 | 4.315068493 | 0 | 1.065677 | 0 | 0.1093885 | 0.613138 | 0.1088482 | 0.04797496 | 0.3137074 | 0.3415484 | 1.155801636 | high |
| TCGA-BQ-5878 | 5.309589041 | 0 | 2.221425 | 0.03570142 | 0.05461617 | 0.8571676 | 0.05928697 | 0.1676726 | 0.04085994 | 0.2700065 | 0.95895142 | low |
| TCGA-DW-7834 | 10.82191781 | 0 | 1.69037 | 0.1019511 | 0.1169739 | 0.6410851 | 0.1551948 | 0.0684023 | 0.07778806 | 0.3043605 | 0.833349476 | low |
| TCGA-B1-5398 | 3.42739726 | 0 | 1.266574 | 0.1401809 | 0.06969603 | 1.041749 | 0.01939909 | 0.02351298 | 0.02673929 | 0 | 1.480233702 | high |
| TCGA-G7-7501 | 1.709589041 | 1 | 0.8319734 | 0.04294141 | 0.07226114 | 4.860406 | 0.04753996 | 0.3169186 | 0.1146741 | 0.1025564 | 8.204347381 | high |
| TCGA-4A-A93X | 1.068493151 | 0 | 0.5524535 | 1.019386 | 0.07088457 | 0.9535631 | 0 | 0.03108813 | 0 | 0.3319889 | 4.482419659 | high |
| TCGA-MH-A562 | 1.687671233 | 0 | 3.350411 | 0 | 0.2220518 | 0.1619033 | 0.705488 | 0 | 0.9994413 | 1.014617 | 0.088882163 | low |
| TCGA-2Z-A9JG | 4.553424658 | 0 | 0.9436848 | 0.04605051 | 0.3628085 | 0.4211972 | 0.127455 | 0.2162774 | 1.177064 | 0.4582575 | 1.618847459 | high |
| TCGA-GL-A59T | 1.495890411 | 0 | 4.392595 | 0 | 0.1421273 | 0.8058 | 0.1596021 | 0 | 0.1466614 | 1.173185 | 0.365141755 | low |
| TCGA-GL-7966 | 0.306849315 | 0 | 0.147993 | 0.6454532 | 0.4215511 | 6.017115 | 0.06870905 | 0.732863 | 0.7576566 | 0.118579 | 42.83016461 | high |
| TCGA-GL-6846 | 16.23287671 | 0 | 0.7434752 | 0.04072307 | 0.1152519 | 2.048586 | 0 | 0.05464486 | 0.01553573 | 0.1945167 | 2.955889541 | high |
| TCGA-B1-A47M | 1.805479452 | 0 | 1.550793 | 0 | 0.141497 | 0.2643702 | 0.2079971 | 0.07757115 | 1.279118 | 0.8974107 | 0.877498395 | low |
| TCGA-B9-7268 | 1.838356164 | 0 | 1.572152 | 0.04057247 | 0.06206794 | 0.5102526 | 0.02245867 | 0.08166416 | 0.3869568 | 0.8882379 | 1.529857219 | high |
| TCGA-HE-7128 | NA | 0 | 1.090895 | 0.05228356 | 0.1039787 | 1.01619 | 0.08682383 | 0.2104724 | 0.1396221 | 0.1456795 | 1.529609751 | high |
| TCGA-UZ-A9PX | 5.42739726 | 0 | 2.246846 | 0.1482876 | 0.2986872 | 0.5086127 | 0.2325708 | 0.6301095 | 0.6977123 | 0.4525292 | 1.005217219 | low |
| TCGA-GL-A4EM | 0.087671233 | 0 | 1.335921 | 1.308995 | 0.174131 | 0.5856172 | 0.157519 | 0 | 0 | 0.2718483 | 2.176048828 | high |
| TCGA-A4-7286 | 1.517808219 | 0 | 1.084424 | 0 | 0.2357213 | 0.5872226 | 0 | 0 | 3.722942 | 0.6133355 | 2.451948459 | high |
| TCGA-2Z-A9JQ | 2.131506849 | 0 | 3.711185 | 0.1276992 | 0.2148902 | 0 | 0.4948103 | 0 | 0 | 1.575742 | 0.171456991 | low |
| TCGA-UZ-A9PU | 2.556164384 | 1 | 2.826208 | 0.1548026 | 0.1894541 | 0.1769861 | 0.04284506 | 1.107862 | 0.5511959 | 0.4107921 | 1.722441877 | high |
| TCGA-2Z-A9J9 | 6.284931507 | 0 | 3.355758 | 0 | 0.9136433 | 0.4955902 | 0.4532321 | 0.03231454 | 0.2572398 | 0 | 0.187078745 | low |
| TCGA-2Z-A9JP | 2.446575342 | 0 | 2.256746 | 0.05823976 | 0.2227386 | 0.1997569 | 0.0322383 | 0.07814988 | 0 | 0.788195 | 1.007643742 | low |
| TCGA-EV-5901 | 2.849315068 | 0 | 1.930105 | 0.4430482 | 0.2476493 | 1.675472 | 0.02829772 | 0.114329 | 0.03900496 | 0.02713143 | 2.04728136 | high |
| TCGA-A4-7287 | 1.635616438 | 1 | 1.96503 | 0.5859991 | 0.0647446 | 9.379636 | 0 | 0.524221 | 0.2235568 | 0.02591729 | 53.95335845 | high |
| TCGA-A4-8311 | 1.997260274 | 0 | 2.734077 | 0.231728 | 0.06499138 | 0.5298703 | 0 | 0.6218953 | 1.50286 | 0.4919403 | 1.747839394 | high |
| TCGA-BQ-5888 | 1.104109589 | 0 | 0.8902075 | 0 | 0.2105645 | 0.6532167 | 0.6469022 | 0.1393934 | 0.3764854 | 0.1447227 | 0.258756743 | low |
| TCGA-UZ-A9PP | 8.246575342 | 0 | 1.777651 | 0.04259894 | 0.1042688 | 0.4383311 | 0.1061118 | 0.228648 | 0.09750817 | 0.3899974 | 0.976501003 | low |
| TCGA-BQ-5892 | 0.997260274 | 0 | 1.229692 | 0.04521094 | 0.4115251 | 1.447313 | 0 | 0.09100045 | 13.10835 | 0 | 7.062276706 | high |
| TCGA-B9-5156 | 3.665753425 | 0 | 1.054755 | 0.2535119 | 0.08402842 | 2.318725 | 0 | 0.08504466 | 0.5158079 | 0.1850013 | 3.546465551 | high |
| TCGA-IA-A83V | 8.057534247 | 1 | 3.244810399 | 0 | 0.154639571 | 0.088900151 | 0.215210774 | 0.104339849 | 0.682276318 | 0.526169334 | 0.337152147 | low |
| TCGA-MH-A55Z | 2.315068493 | 0 | 3.012136 | 0.5434853 | 0.4936592 | 0.077671 | 0.3572514 | 0.04558024 | 0.6997656 | 1.703623 | 0.64056832 | low |
| TCGA-UZ-A9Q1 | 2 | 0 | 1.045732 | 0.04677775 | 0.2289945 | 0.534811 | 0.2848292 | 0.06276941 | 0.01784557 | 0.1303384 | 0.656450592 | low |
| TCGA-B1-A656 | 1.693150685 | 0 | 0.9531731 | 0 | 0.1992384 | 0.05317902 | 0 | 0.06241486 | 0.301661 | 0.0185146 | 1.267318954 | high |
| TCGA-SX-A7SQ | 3.468493151 | 0 | 2.203297 | 0.1046634 | 0.1280916 | 0.957295 | 0.1303556 | 0.1404441 | 1.497328 | 0.2291356 | 0.965968024 | low |
| TCGA-G7-6789 | 0.334246575 | 1 | 0.7387804 | 0.1239269 | 1.52931 | 1.464087 | 0 | 0.02771552 | 28.03569 | 0.03288588 | 57.5415861 | high |
| TCGA-AL-3468 | 6.38630137 | 1 | 0.6682811 | 0.2869785 | 0.1920716 | 0.8202578 | 0.1389984 | 0.5776294 | 0.1368517 | 0.8567334 | 3.229838018 | high |
| TCGA-B1-A655 | 1.397260274 | 0 | 0.4039884 | 0 | 0.5550355 | 1.430369 | 0.03462657 | 0.08393937 | 0.01590951 | 0.03319945 | 2.767310262 | high |
| TCGA-5P-A9JV | 5.597260274 | 0 | 2.698482129 | 0 | 0.490060864 | 1.337601314 | 0.061677802 | 0.149515112 | 18.7883922 | 0.044351825 | 5.559717971 | high |
| TCGA-5P-A9K9 | 1.61369863 | 1 | 1.313264 | 1.036677 | 0.42291 | 4.345861 | 0.01912822 | 0.278216 | 1.081003 | 3.081096 | 52.14580031 | high |
| TCGA-A4-A772 | 0 | 0 | 1.719341 | 0.08874194 | 0.08824256 | 0.5072943 | 0.2333324 | 0.1190797 | 0.6093859 | 0.2296029 | 0.642849065 | low |
| TCGA-BQ-5880 | 3.6 | 1 | 0.487032 | 0.4815841 | 1.331375 | 0.9438791 | 0.09520651 | 0 | 0.02624612 | 0.3286173 | 3.96092249 | high |

# Appendix 6

**gene set enrichment analyses (GSEA)**

**Table 6a. GSEA** of high rish.

| NAME | SIZE | ES | NES | NOM p-val | FDR q-val |
| --- | --- | --- | --- | --- | --- |
| KEGG_ECM_RECEPTOR_INTERACTION | 84 | 0.6350506 | 1.9481883 | 0.007968128 | 0.19975616 |
| KEGG_CELL_CYCLE | 125 | 0.6064889 | 1.9121977 | 0.009881423 | 0.15662064 |
| KEGG_MATURITY_ONSET_DIABETES_OF_THE_YOUNG | 25 | 0.579834 | 1.7955704 | 0 | 0.32934836 |
| KEGG_CYTOKINE_CYTOKINE_RECEPTOR_INTERACTION | 264 | 0.4991102 | 1.7905463 | 0.00814664 | 0.2581876 |
| KEGG_DNA_REPLICATION | 36 | 0.6930627 | 1.7698328 | 0.019607844 | 0.26072094 |
| KEGG_HEMATOPOIETIC_CELL_LINEAGE | 85 | 0.5703835 | 1.7679955 | 0.014403292 | 0.21927755 |
| KEGG_P53_SIGNALING_PATHWAY | 68 | 0.5277478 | 1.764059 | 0.025948104 | 0.19482152 |
| KEGG_PROGESTERONE_MEDIATED_OOCYTE_MATURATION | 85 | 0.5003824 | 1.7592667 | 0.007736944 | 0.17766927 |
| KEGG_CHEMOKINE_SIGNALING_PATHWAY | 188 | 0.5222193 | 1.7468793 | 0.028397566 | 0.1729043 |
| KEGG_PATHOGENIC_ESCHERICHIA_COLI_INFECTION | 56 | 0.53929913 | 1.7339888 | 0.022 | 0.17158702 |
| KEGG_NATURAL_KILLER_CELL_MEDIATED_CYTOTOXICITY | 132 | 0.5222277 | 1.690277 | 0.014644352 | 0.22533338 |
| KEGG_REGULATION_OF_ACTIN_CYTOSKELETON | 213 | 0.45684168 | 1.6862986 | 0.012048192 | 0.21235943 |
| KEGG_SMALL_CELL_LUNG_CANCER | 84 | 0.5155415 | 1.6798103 | 0.025540275 | 0.20611957 |
| KEGG_PRIMARY_IMMUNODEFICIENCY | 35 | 0.6675987 | 1.6783099 | 0.031120332 | 0.19293354 |
| KEGG_CELL_ADHESION_MOLECULES_CAMS | 131 | 0.5109711 | 1.6724298 | 0.043912176 | 0.18752038 |
| KEGG_FC_GAMMA_R_MEDIATED_PHAGOCYTOSIS | 96 | 0.492863 | 1.6705801 | 0.027722772 | 0.17802949 |
| KEGG_OOCYTE_MEIOSIS | 113 | 0.4725066 | 1.6629518 | 0.028901733 | 0.1771297 |
| KEGG_NOD_LIKE_RECEPTOR_SIGNALING_PATHWAY | 62 | 0.52476937 | 1.628585 | 0.05 | 0.2098455 |
| KEGG_HOMOLOGOUS_RECOMBINATION | 28 | 0.6004974 | 1.6239578 | 0.042168673 | 0.20566446 |
| KEGG_CYTOSOLIC_DNA_SENSING_PATHWAY | 55 | 0.49848208 | 1.6165303 | 0.030303031 | 0.20612346 |
| KEGG_NEUROACTIVE_LIGAND_RECEPTOR_INTERACTION | 270 | 0.395095 | 1.5873286 | 0.01183432 | 0.23827425 |
| KEGG_MISMATCH_REPAIR | 23 | 0.6271522 | 1.5827314 | 0.060784314 | 0.23391642 |
| KEGG_FOCAL_ADHESION | 199 | 0.4614525 | 1.5760583 | 0.05645161 | 0.23295976 |
| KEGG_PATHWAYS_IN_CANCER | 325 | 0.4182549 | 1.5583463 | 0.053784862 | 0.2521694 |
| KEGG_PANCREATIC_CANCER | 70 | 0.48699772 | 1.5448018 | 0.07042254 | 0.2643852 |
| KEGG_JAK_STAT_SIGNALING_PATHWAY | 155 | 0.4254083 | 1.5416272 | 0.03846154 | 0.25919008 |
| KEGG_T_CELL_RECEPTOR_SIGNALING_PATHWAY | 108 | 0.47600168 | 1.5270414 | 0.091649696 | 0.27102333 |
| KEGG_NON_SMALL_CELL_LUNG_CANCER | 54 | 0.45931154 | 1.525343 | 0.070881225 | 0.26388898 |
| KEGG_BLADDER_CANCER | 42 | 0.46921286 | 1.4996709 | 0.0662768 | 0.29494244 |
| KEGG_PRION_DISEASES | 35 | 0.47422987 | 1.4893994 | 0.06225681 | 0.29993868 |
| KEGG_INTESTINAL_IMMUNE_NETWORK_FOR_IGA_PRODUCTION | 46 | 0.5618506 | 1.4820112 | 0.114107884 | 0.3022052 |
| KEGG_ABC_TRANSPORTERS | 44 | 0.43016574 | 1.4681667 | 0.06407767 | 0.31230438 |
| KEGG_PROSTATE_CANCER | 89 | 0.44012326 | 1.4600335 | 0.1015625 | 0.31445095 |
| KEGG_TOLL_LIKE_RECEPTOR_SIGNALING_PATHWAY | 102 | 0.44132376 | 1.4543095 | 0.07444668 | 0.31452712 |
| KEGG_MAPK_SIGNALING_PATHWAY | 267 | 0.3696626 | 1.443646 | 0.046184737 | 0.3205885 |
| KEGG_LONG_TERM_DEPRESSION | 70 | 0.40286198 | 1.4398769 | 0.06560636 | 0.31771284 |
| KEGG_MELANOMA | 71 | 0.40501207 | 1.4292448 | 0.06882591 | 0.32481202 |
| KEGG_LEUKOCYTE_TRANSENDOTHELIAL_MIGRATION | 116 | 0.40948707 | 1.4195328 | 0.082474224 | 0.33044267 |
| KEGG_BASAL_TRANSCRIPTION_FACTORS | 35 | 0.48116967 | 1.4160656 | 0.11306043 | 0.32719412 |
| KEGG_LEISHMANIA_INFECTION | 70 | 0.52667886 | 1.4133627 | 0.17012449 | 0.322644 |
| KEGG_AXON_GUIDANCE | 129 | 0.4008033 | 1.4051335 | 0.10778443 | 0.32654884 |
| KEGG_GAP_JUNCTION | 90 | 0.3966258 | 1.3986498 | 0.098425195 | 0.32851103 |
| KEGG_ANTIGEN_PROCESSING_AND_PRESENTATION | 81 | 0.48021176 | 1.3951766 | 0.1530815 | 0.32624608 |
| KEGG_ARRHYTHMOGENIC_RIGHT_VENTRICULAR_CARDIOMYOPATHY_ARVC | 74 | 0.41542795 | 1.388575 | 0.119373776 | 0.32914373 |
| KEGG_GLIOMA | 65 | 0.42001376 | 1.387907 | 0.10843374 | 0.32290292 |
| KEGG_MELANOGENESIS | 101 | 0.38515013 | 1.3876063 | 0.10576923 | 0.3161987 |
| KEGG_VASCULAR_SMOOTH_MUSCLE_CONTRACTION | 115 | 0.38769728 | 1.3866826 | 0.11706349 | 0.31027707 |
| KEGG_N_GLYCAN_BIOSYNTHESIS | 46 | 0.45841998 | 1.3847407 | 0.14478764 | 0.30624327 |
| KEGG_GLYCOSAMINOGLYCAN_BIOSYNTHESIS_CHONDROITIN_SULFATE | 22 | 0.49131438 | 1.3721334 | 0.1523046 | 0.3183949 |
| KEGG_BASE_EXCISION_REPAIR | 35 | 0.46448472 | 1.3708931 | 0.16472869 | 0.31336427 |
| KEGG_BASAL_CELL_CARCINOMA | 55 | 0.40963164 | 1.3667789 | 0.12185687 | 0.31273282 |
| KEGG_SYSTEMIC_LUPUS_ERYTHEMATOSUS | 135 | 0.40523705 | 1.3612336 | 0.15541922 | 0.31337053 |
| KEGG_CALCIUM_SIGNALING_PATHWAY | 178 | 0.35589138 | 1.3594975 | 0.09311741 | 0.30964553 |
| KEGG_ACUTE_MYELOID_LEUKEMIA | 57 | 0.42234305 | 1.3514103 | 0.15686275 | 0.3143376 |
| KEGG_COLORECTAL_CANCER | 62 | 0.4312545 | 1.3511295 | 0.16330644 | 0.3087993 |
| KEGG_AUTOIMMUNE_THYROID_DISEASE | 50 | 0.49899828 | 1.340961 | 0.20040485 | 0.31544992 |
| KEGG_ASTHMA | 28 | 0.5422644 | 1.3403034 | 0.21314742 | 0.31086293 |
| KEGG_GRAFT_VERSUS_HOST_DISEASE | 37 | 0.5974532 | 1.3360924 | 0.23640168 | 0.31060335 |
| KEGG_NOTCH_SIGNALING_PATHWAY | 47 | 0.42137018 | 1.3258299 | 0.15506959 | 0.31843626 |
| KEGG_APOPTOSIS | 87 | 0.38375717 | 1.3210187 | 0.16860466 | 0.31926286 |
| KEGG_HYPERTROPHIC_CARDIOMYOPATHY_HCM | 83 | 0.3801523 | 1.3184363 | 0.1403162 | 0.3172558 |
| KEGG_B_CELL_RECEPTOR_SIGNALING_PATHWAY | 75 | 0.4073386 | 1.316415 | 0.18253969 | 0.3146218 |
| KEGG_ERBB_SIGNALING_PATHWAY | 87 | 0.38926023 | 1.3123038 | 0.171875 | 0.31417173 |
| KEGG_CHRONIC_MYELOID_LEUKEMIA | 73 | 0.41120157 | 1.3017542 | 0.18897638 | 0.3223564 |
| KEGG_ALLOGRAFT_REJECTION | 35 | 0.58649325 | 1.2973398 | 0.26694044 | 0.32257095 |
| KEGG_ADHERENS_JUNCTION | 73 | 0.418484 | 1.2947649 | 0.23061225 | 0.32108206 |
| KEGG_THYROID_CANCER | 29 | 0.42412338 | 1.2836252 | 0.19066148 | 0.33028895 |
| KEGG_TGF_BETA_SIGNALING_PATHWAY | 86 | 0.39678797 | 1.2820654 | 0.21272366 | 0.32743192 |
| KEGG_VIRAL_MYOCARDITIS | 68 | 0.42351252 | 1.2702105 | 0.23076923 | 0.3374746 |
| KEGG_FC_EPSILON_RI_SIGNALING_PATHWAY | 79 | 0.3516723 | 1.2701744 | 0.16901408 | 0.33266774 |
| KEGG_NUCLEOTIDE_EXCISION_REPAIR | 44 | 0.43859223 | 1.2662205 | 0.22330096 | 0.33282524 |
| KEGG_WNT_SIGNALING_PATHWAY | 151 | 0.34958127 | 1.2658015 | 0.19493178 | 0.32874882 |
| KEGG_RIG_I_LIKE_RECEPTOR_SIGNALING_PATHWAY | 71 | 0.3540985 | 1.2539523 | 0.2 | 0.33923385 |
| KEGG_MTOR_SIGNALING_PATHWAY | 52 | 0.35837713 | 1.2382987 | 0.20676692 | 0.35338014 |
| KEGG_NEUROTROPHIN_SIGNALING_PATHWAY | 126 | 0.36043835 | 1.2365556 | 0.21442495 | 0.3509036 |
| KEGG_AMINO_SUGAR_AND_NUCLEOTIDE_SUGAR_METABOLISM | 44 | 0.39737117 | 1.2357852 | 0.22289157 | 0.34695372 |
| KEGG_TYPE_I_DIABETES_MELLITUS | 41 | 0.50289935 | 1.231867 | 0.31440163 | 0.34705564 |
| KEGG_HEDGEHOG_SIGNALING_PATHWAY | 56 | 0.36493695 | 1.2257366 | 0.21346153 | 0.35056213 |
| KEGG_DILATED_CARDIOMYOPATHY | 90 | 0.353731 | 1.215113 | 0.20517929 | 0.35953906 |
| KEGG_STEROID_BIOSYNTHESIS | 17 | 0.46402398 | 1.2066244 | 0.24710424 | 0.36566636 |
| KEGG_ENDOMETRIAL_CANCER | 52 | 0.38824615 | 1.2008873 | 0.2626459 | 0.36857376 |
| KEGG_RENAL_CELL_CARCINOMA | 70 | 0.37331426 | 1.196673 | 0.25 | 0.3694536 |
| KEGG_PHOSPHATIDYLINOSITOL_SIGNALING_SYSTEM | 76 | 0.36663106 | 1.1955342 | 0.26693228 | 0.36621684 |
| KEGG_UBIQUITIN_MEDIATED_PROTEOLYSIS | 135 | 0.35834804 | 1.195445 | 0.2944664 | 0.36195916 |
| KEGG_DORSO_VENTRAL_AXIS_FORMATION | 24 | 0.41796407 | 1.1878082 | 0.2966805 | 0.36705774 |
| KEGG_TYPE_II_DIABETES_MELLITUS | 47 | 0.36304983 | 1.1715912 | 0.23613963 | 0.38304695 |
| KEGG_PYRIMIDINE_METABOLISM | 98 | 0.3405534 | 1.1707062 | 0.27058825 | 0.38005194 |
| KEGG_GNRH_SIGNALING_PATHWAY | 101 | 0.3140828 | 1.1601273 | 0.275154 | 0.3891004 |
| KEGG_NITROGEN_METABOLISM | 23 | 0.3623677 | 1.1534523 | 0.26403326 | 0.39339554 |
| KEGG_GLYCOSPHINGOLIPID_BIOSYNTHESIS_GANGLIO_SERIES | 15 | 0.43971354 | 1.147137 | 0.29622266 | 0.39663658 |
| KEGG_STEROID_HORMONE_BIOSYNTHESIS | 55 | 0.33968356 | 1.128262 | 0.27944112 | 0.41659266 |
| KEGG_TASTE_TRANSDUCTION | 51 | 0.33475435 | 1.1205332 | 0.2934363 | 0.42226794 |
| KEGG_PURINE_METABOLISM | 159 | 0.2927696 | 1.1199157 | 0.308 | 0.41863328 |
| KEGG_EPITHELIAL_CELL_SIGNALING_IN_HELICOBACTER_PYLORI_INFECTION | 68 | 0.33441237 | 1.1063426 | 0.3233533 | 0.43088606 |
| KEGG_INSULIN_SIGNALING_PATHWAY | 137 | 0.29590127 | 1.0967119 | 0.3504762 | 0.438988 |
| KEGG_GLYCOSAMINOGLYCAN_BIOSYNTHESIS_KERATAN_SULFATE | 15 | 0.41591293 | 1.0854985 | 0.36561266 | 0.44948682 |
| KEGG_LONG_TERM_POTENTIATION | 70 | 0.31262138 | 1.0566427 | 0.37525356 | 0.48487574 |
| KEGG_ADIPOCYTOKINE_SIGNALING_PATHWAY | 67 | 0.305643 | 1.0539336 | 0.3964497 | 0.48397318 |
| KEGG_INOSITOL_PHOSPHATE_METABOLISM | 54 | 0.32741114 | 1.0342128 | 0.42524272 | 0.50750774 |
| KEGG_OLFACTORY_TRANSDUCTION | 386 | 0.27532697 | 1.0175565 | 0.43639922 | 0.5262828 |
| KEGG_OTHER_GLYCAN_DEGRADATION | 16 | 0.4177695 | 1.016066 | 0.46107784 | 0.5229609 |
| KEGG_DRUG_METABOLISM_OTHER_ENZYMES | 51 | 0.28834903 | 0.99945223 | 0.42322835 | 0.5410469 |
| KEGG_VEGF_SIGNALING_PATHWAY | 76 | 0.2686352 | 0.9866887 | 0.45167652 | 0.55505514 |
| KEGG_ENDOCYTOSIS | 181 | 0.27033362 | 0.9833469 | 0.47686118 | 0.554294 |
| KEGG_STARCH_AND_SUCROSE_METABOLISM | 52 | 0.2756888 | 0.95902884 | 0.528827 | 0.5846599 |
| KEGG_LINOLEIC_ACID_METABOLISM | 29 | 0.30678797 | 0.9550766 | 0.5137255 | 0.5850361 |
| KEGG_COMPLEMENT_AND_COAGULATION_CASCADES | 69 | 0.3066265 | 0.95389014 | 0.5069307 | 0.5813898 |
| KEGG_GLYCEROPHOSPHOLIPID_METABOLISM | 77 | 0.24590448 | 0.9523727 | 0.5323887 | 0.57825303 |
| KEGG_PROTEIN_EXPORT | 24 | 0.35821387 | 0.94441676 | 0.51937985 | 0.5845025 |
| KEGG_ARACHIDONIC_ACID_METABOLISM | 58 | 0.2636029 | 0.94250345 | 0.52286285 | 0.5820304 |
| KEGG_O_GLYCAN_BIOSYNTHESIS | 30 | 0.30639446 | 0.9340918 | 0.54268295 | 0.58928454 |
| KEGG_NICOTINATE_AND_NICOTINAMIDE_METABOLISM | 24 | 0.28934965 | 0.9160494 | 0.5737374 | 0.61202383 |
| KEGG_GLYCOSPHINGOLIPID_BIOSYNTHESIS_LACTO_AND_NEOLACTO_SERIES | 26 | 0.2880131 | 0.9076522 | 0.55852157 | 0.6197325 |
| KEGG_GLUTATHIONE_METABOLISM | 49 | 0.27540466 | 0.905479 | 0.5647773 | 0.61768824 |
| KEGG_BIOSYNTHESIS_OF_UNSATURATED_FATTY_ACIDS | 22 | 0.3155005 | 0.90193564 | 0.5743381 | 0.6175407 |
| KEGG_RNA_DEGRADATION | 59 | 0.2784789 | 0.86100566 | 0.63241106 | 0.67484 |
| KEGG_TIGHT_JUNCTION | 132 | 0.23396872 | 0.8601693 | 0.61100197 | 0.67040515 |
| KEGG_ALANINE_ASPARTATE_AND_GLUTAMATE_METABOLISM | 32 | 0.29898274 | 0.8561269 | 0.6 | 0.67074656 |
| KEGG_CYSTEINE_AND_METHIONINE_METABOLISM | 34 | 0.27411413 | 0.84666854 | 0.6425781 | 0.6793706 |
| KEGG_PENTOSE_PHOSPHATE_PATHWAY | 27 | 0.2856239 | 0.84319013 | 0.6264591 | 0.67951715 |
| KEGG_SPLICEOSOME | 127 | 0.28068334 | 0.83768445 | 0.6146245 | 0.68183506 |
| KEGG_SNARE_INTERACTIONS_IN_VESICULAR_TRANSPORT | 38 | 0.2641218 | 0.82714486 | 0.6745098 | 0.6924281 |
| KEGG_VIBRIO_CHOLERAE_INFECTION | 54 | 0.26069286 | 0.81419873 | 0.6858847 | 0.706157 |
| KEGG_GALACTOSE_METABOLISM | 26 | 0.26025012 | 0.8125401 | 0.72319686 | 0.702997 |
| KEGG_RETINOL_METABOLISM | 64 | 0.2326401 | 0.7957541 | 0.7659575 | 0.72228736 |
| KEGG_GLYCOSYLPHOSPHATIDYLINOSITOL_GPI_ANCHOR_BIOSYNTHESIS | 25 | 0.2823819 | 0.7956789 | 0.6891892 | 0.7166494 |
| KEGG_ONE_CARBON_POOL_BY_FOLATE | 17 | 0.2913929 | 0.7736211 | 0.704 | 0.7445511 |
| KEGG_AMYOTROPHIC_LATERAL_SCLEROSIS_ALS | 53 | 0.21686848 | 0.769832 | 0.84479374 | 0.7445245 |
| KEGG_ALPHA_LINOLENIC_ACID_METABOLISM | 19 | 0.27887446 | 0.7572793 | 0.7687366 | 0.75713825 |
| KEGG_RNA_POLYMERASE | 29 | 0.2524023 | 0.7334156 | 0.7779886 | 0.7868788 |
| KEGG_PENTOSE_AND_GLUCURONATE_INTERCONVERSIONS | 28 | 0.25916424 | 0.73334634 | 0.7936842 | 0.78102964 |
| KEGG_ALDOSTERONE_REGULATED_SODIUM_REABSORPTION | 42 | 0.2415794 | 0.70590174 | 0.8427419 | 0.8140115 |
| KEGG_ASCORBATE_AND_ALDARATE_METABOLISM | 25 | 0.25248286 | 0.68439674 | 0.82186234 | 0.8364016 |
| KEGG_LYSOSOME | 121 | 0.22004794 | 0.68438923 | 0.7897839 | 0.83018064 |
| KEGG_GLYCEROLIPID_METABOLISM | 49 | 0.19314307 | 0.6835444 | 0.9329389 | 0.8250762 |
| KEGG_GLYCOSAMINOGLYCAN_DEGRADATION | 21 | 0.22821651 | 0.6538695 | 0.9050388 | 0.85487825 |
| KEGG_ETHER_LIPID_METABOLISM | 33 | 0.13067183 | 0.42815334 | 1 | 0.9890834 |

**Table 6b. GSEA of low rish.**

| NAME | SIZE | ES | NES | NOM p-val | FDR q-val |
| --- | --- | --- | --- | --- | --- |
| KEGG_HUNTINGTONS_DISEASE | 182 | -0.583765 | -2.020493 | 0.00390625 | 0.027066298 |
| KEGG_OXIDATIVE_PHOSPHORYLATION | 132 | -0.7714413 | -2.0131025 | 0.003875969 | 0.01511863 |
| KEGG_PARKINSONS_DISEASE | 130 | -0.71407384 | -1.979419 | 0.003913894 | 0.014085387 |
| KEGG_ALZHEIMERS_DISEASE | 166 | -0.5594885 | -1.887964 | 0.003868472 | 0.027649669 |
| KEGG_CARDIAC_MUSCLE_CONTRACTION | 79 | -0.51520324 | -1.7584441 | 0.015748031 | 0.0831413 |
| KEGG_CITRATE_CYCLE_TCA_CYCLE | 31 | -0.7674336 | -1.6918622 | 0.033009708 | 0.11904526 |
| KEGG_FATTY_ACID_METABOLISM | 42 | -0.6384664 | -1.6393366 | 0.057803467 | 0.14947033 |
| KEGG_PROXIMAL_TUBULE_BICARBONATE_RECLAMATION | 23 | -0.6288686 | -1.6159911 | 0.031496063 | 0.1548862 |
| KEGG_VALINE_LEUCINE_AND_ISOLEUCINE_DEGRADATION | 44 | -0.6775985 | -1.536997 | 0.10958904 | 0.22621053 |
| KEGG_PROPANOATE_METABOLISM | 33 | -0.6560805 | -1.5292882 | 0.091816366 | 0.21223734 |
| KEGG_PYRUVATE_METABOLISM | 40 | -0.5485985 | -1.5129263 | 0.1017964 | 0.21180427 |
| KEGG_BUTANOATE_METABOLISM | 34 | -0.59496963 | -1.5115331 | 0.1021611 | 0.19544703 |
| KEGG_ARGININE_AND_PROLINE_METABOLISM | 54 | -0.461703 | -1.4869592 | 0.068897635 | 0.20540513 |
| KEGG_PEROXISOME | 78 | -0.46756575 | -1.4430531 | 0.115913555 | 0.2359625 |
| KEGG_GLYCINE_SERINE_AND_THREONINE_METABOLISM | 31 | -0.5197637 | -1.3913288 | 0.13867188 | 0.27845943 |
| KEGG_TYROSINE_METABOLISM | 42 | -0.41870907 | -1.3673837 | 0.08416834 | 0.29151756 |
| KEGG_TERPENOID_BACKBONE_BIOSYNTHESIS | 15 | -0.5186846 | -1.3124286 | 0.18426502 | 0.3454152 |
| KEGG_HISTIDINE_METABOLISM | 29 | -0.44899052 | -1.2958661 | 0.18019801 | 0.34955582 |
| KEGG_RENIN_ANGIOTENSIN_SYSTEM | 17 | -0.44461396 | -1.2708209 | 0.17296223 | 0.36539757 |
| KEGG_TRYPTOPHAN_METABOLISM | 40 | -0.40670854 | -1.1790707 | 0.27539062 | 0.4787554 |
| KEGG_RIBOFLAVIN_METABOLISM | 16 | -0.40010884 | -1.1604397 | 0.2829787 | 0.48514423 |
| KEGG_GLYCOLYSIS_GLUCONEOGENESIS | 62 | -0.37114462 | -1.1408279 | 0.29740518 | 0.4943913 |
| KEGG_RIBOSOME | 88 | -0.54288334 | -1.0908462 | 0.45995894 | 0.5544652 |
| KEGG_GLYOXYLATE_AND_DICARBOXYLATE_METABOLISM | 16 | -0.41038185 | -1.0483048 | 0.38814533 | 0.6052204 |
| KEGG_FRUCTOSE_AND_MANNOSE_METABOLISM | 34 | -0.33263627 | -1.0352657 | 0.41020408 | 0.60281193 |
| KEGG_GLYCOSAMINOGLYCAN_BIOSYNTHESIS_HEPARAN_SULFATE | 26 | -0.3385442 | -1.0057067 | 0.45714286 | 0.63039905 |
| KEGG_LYSINE_DEGRADATION | 44 | -0.33439648 | -0.9981058 | 0.46734694 | 0.61957806 |
| KEGG_AMINOACYL_TRNA_BIOSYNTHESIS | 41 | -0.37278908 | -0.99490154 | 0.4796748 | 0.6029711 |
| KEGG_PHENYLALANINE_METABOLISM | 18 | -0.34612867 | -0.98292446 | 0.47316104 | 0.600626 |
| KEGG_PRIMARY_BILE_ACID_BIOSYNTHESIS | 16 | -0.3613452 | -0.9740329 | 0.48906562 | 0.59458727 |
| KEGG_REGULATION_OF_AUTOPHAGY | 35 | -0.2841978 | -0.9314352 | 0.52665246 | 0.63920116 |
| KEGG_SELENOAMINO_ACID_METABOLISM | 26 | -0.30806032 | -0.9219956 | 0.54325956 | 0.6345546 |
| KEGG_VASOPRESSIN_REGULATED_WATER_REABSORPTION | 44 | -0.27898854 | -0.87718725 | 0.608871 | 0.68444514 |
| KEGG_DRUG_METABOLISM_CYTOCHROME_P450 | 71 | -0.24738698 | -0.8505797 | 0.7020408 | 0.70856625 |
| KEGG_BETA_ALANINE_METABOLISM | 22 | -0.32995632 | -0.8412594 | 0.62741315 | 0.70276 |
| KEGG_METABOLISM_OF_XENOBIOTICS_BY_CYTOCHROME_P450 | 69 | -0.24800365 | -0.84117603 | 0.6812749 | 0.683338 |
| KEGG_PORPHYRIN_AND_CHLOROPHYLL_METABOLISM | 41 | -0.26275223 | -0.8173544 | 0.6933868 | 0.7015163 |
| KEGG_SPHINGOLIPID_METABOLISM | 39 | -0.24403013 | -0.7821981 | 0.7505071 | 0.7365701 |
| KEGG_PANTOTHENATE_AND_COA_BIOSYNTHESIS | 16 | -0.2725378 | -0.74909526 | 0.77160496 | 0.76636297 |
| KEGG_PPAR_SIGNALING_PATHWAY | 69 | -0.23009433 | -0.7124924 | 0.8247012 | 0.7969876 |
| KEGG_PROTEASOME | 46 | -0.2056966 | -0.5231288 | 0.9034205 | 0.96061766 |
